# Wheldone Revisited: Structure Revision Via DFT-GIAO Chemical Shift Calculations, 1,1-HD-ADEQUATE NMR Spectroscopy, and X-ray Crystallography Studies

**DOI:** 10.1021/acs.jnatprod.4c00649

**Published:** 2024-07-23

**Authors:** Manuel Rangel-Grimaldo, Cody E. Earp, Huzefa A. Raja, Jared S. Wood, Lina Mardiana, Kin Lok Ho, Alexandra Longcake, R. Thomas Williamson, Lukáš Palatinus, Michael J. Hall, Michael R. Probert, Nicholas H. Oberlies

**Affiliations:** † Department of Chemistry and Biochemistry, University of North Carolina at Greensboro, Greensboro, North Carolina 27402, United States; ‡ Department of Chemistry and Biochemistry, University of North Carolina Wilmington, Wilmington, North Carolina 28409, United States; § Indicatrix Crystallography Ltd, Newcastle University, Newcastle NE1 7RU, U.K.; ∥ Chemistry − School of Natural and Environmental Sciences, Newcastle University, Newcastle NE1 7RU, U.K.; ⊥ Department of Chemistry, Universitas Indonesia, Depok, Jawa Barat 16424, Indonesia; # Department of Structure Analysis, Institute of Physics of the Czech Academy of Sciences, Na Slovance 2, Prague 18221, Czech Republic

## Abstract

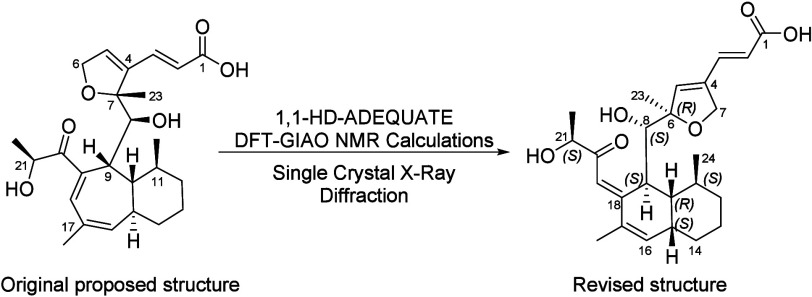

Wheldone is a fungal metabolite isolated from the coculture of *Aspergillus fischeri* and *Xylaria flabelliformis*, displaying cytotoxic activity against breast, melanoma, and ovarian cancer cell lines. Initially, its structure was characterized as an unusual 5-methyl-bicyclo[5.4.0]undeca-3,5-diene scaffold with a 2-hydroxy-1-propanone side chain and a 3-(2-(1-hydroxyethyl)-2-methyl-2,5-dihydrofuran-3-yl)acrylic acid moiety. Upon further examination, minor inconsistencies in the data suggested the need for the structure to be revisited. Thus, the structure of wheldone has been revised using an orthogonal experimental-computational approach, which combines 1,1-HD-ADEQUATE NMR experiments, DFT-GIAO chemical shift calculations, and single-crystal X-ray diffraction (SCXRD) analysis of a semisynthetic *p-*bromobenzylamide derivative, formed via a Steglich-type reaction. The summation of these data now permits the unequivocal assignment of both the structure and absolute configuration of the natural product.

Fungi are a proven source of bioactive compounds, some of which are problematic for our food supply (i.e., mycotoxins),^1−3^ while others have been harnessed for the benefit of humanity.^4,5^ It is now possible to analyze the genetic architecture of fungi,^6^ where the annotation of biosynthetic gene clusters^6,7^ leads to predictions of their ability to biosynthesize far more secondary metabolites than are observed under standard laboratory conditions.^8^ To explain this disconnect, a common hypothesis is that fungi must survive hostile environments in Nature, typically competing for resources with other microorganisms and causing them to generate defensive chemicals.^9^ As such, forcing fungi to “fight” for limited stores of nutrients via fungal–fungal coculturing is a way to trigger the biosynthesis of molecules that may not otherwise be generated in classical laboratory environments.^10−15^

Recently, coculture studies of *Aspergillus fischeri* (strain NRRL181) and *Xylaria flabelliformis* (strain G536)^16^ were shown to stimulate the biosynthesis of a suite of metabolites,^17^ including one cytotoxic compound (i.e., wheldone), which combined an interesting molecular structure with notable activity against breast, melanoma, and ovarian cancer cell lines.^18^ Wheldone was characterized as consisting of an unusual 5-methyl-bicyclo[5.4.0]undeca-3,5-diene scaffold with a 2-hydroxy-1-propanone side chain and a 3-(2-(1-hydroxyethyl)-2-methyl-2,5-dihydrofuran-3-yl)acrylic acid moiety, which were established based on 1D- and 2D-NMR spectroscopy and mass spectrometry data. At that time, the proposed structure was not verified via orthogonal studies such as single-crystal X-ray diffraction (SCXRD). However, upon further optimization of the coculture conditions, it was possible to generate a larger stock of wheldone, which was needed for additional pharmacological studies that will be reported in the future. During these experiments, re-examination of the spectral data highlighted several anomalies in the original structure assignment, and therefore, a more in-depth analysis of the structure elucidation was embarked upon. Thus, the goal of the current study was to re-evaluate the structure assignment of wheldone by an orthogonal experimental-computational approach, through (a) examination of the chemical shift assignments by DFT-calculations, (b) performance of additional NMR studies, particularly 1,1-HD-ADEQUATE,^19^ which provides visualization of carbon–carbon connectivity through the structural backbone, and (c) generation of crystals suitable for SCXRD studies.^20^ Analysis of the data from this combination of molecular characterization techniques led to the structural revision of wheldone. Two of the authors (NHO and HAR) of this manuscript were coauthors of the originally published structure of wheldone and apologize to the scientific community for this error, which has been rectified, *vide infra*.

## Results and Discussion

**Figure 1:**
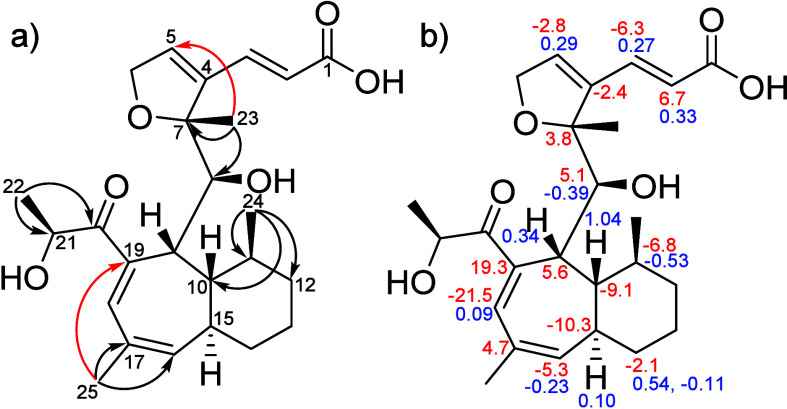
Identification of NMR spectral discrepancies in the originally proposed structure of wheldone. (a) Key HMBC correlations noted in the original publication with ^4^*J*_CH_ in red. (b) Selected Δδ_ppm_^13^C NMR (red) and Δδ_ppm_^1^H NMR (blue) of the experimental vs predicted chemical shifts.

Our re-examination of the wheldone structural assignment began with a comparison of the experimental NMR spectra with those calculated. Initially, an exhaustive conformational search was conducted by MMFF94 force field in Spartan’10 (Wave function). All conformers with relative energies within 5 kcal/mol were then optimized in Gaussian 16 at the M062*X*/6-31+G(d,p) level of theory using the integral equation formalism polarizable continuum model (IEFPCM) in CH_3_OH. Next, the optimized conformers were used to calculate both the ^1^H and ^13^C NMR chemical shifts at the B3LYP/6-311+G(2d,p) level of theory with the IEFPCM, also in CH_3_OH. At first glance, the calculated chemical shifts suggested that the published structure of wheldone indeed displayed structural assignment issues, with mean absolute errors for proton (^1^H-MAE) of 0.24 and 5.9 ppm for carbon (^13^C-MAE) (Table S9). Discrepancies between the calculated and experimental NMR chemical shifts were most evident in the seven-membered ring and the 3-(2,5-dihydrofuran-3-yl)acrylic acid moiety (Figure 1). Specifically, significant errors between calculated and experimental chemical shift values of approximately 20 ppm were noted for both C-18 and C-19, as well as deviations of about 10 ppm for the chemical shifts of C-10 and C-15. Likewise, the chemical shifts from C-2 to C-5 showed deviations that averaged around 5.0 ppm. A similar pattern was observed in the ^1^H NMR data, where major differences (ranging between 0.09 to 1.04 ppm) were observed in both H-3 and H-5, suggesting issues with original assignment of the 3-(2,5-dihydrofuran-3-yl)acrylic acid moiety, and in H-9, H-10, H-11, H-15, and H-18, indicating that revisions might be needed in the bicyclo[5.4.0]undeca-3,5-diene framework.

In reviewing the previous structure elucidation data,^18^ key concerns were also raised with the unusual four-bond HMBC (^4^*J*_CH_) couplings proposed from H_3_-25 to C-19 and from H_3_-23 to C-5 (Figure 1). It is well-established that the intensity of the HMBC cross-peaks depends on the magnitude of ^2^*J*_CH_, ^3^*J*_CH_, and ^4^*J*_CH_ coupling constants as well as the geometry of the coupled atoms. The conventional settings of an HMBC experiment are optimized in the range of 6 to10 Hz, which typically correspond to the magnitude of ^2^*J*_CH_ and ^3^*J*_CH_ correlations and, to a lesser extent, the much smaller ^4^*J*_CH_ couplings.^21^ For example, a weak ^4^*J*_CH_ coupling was observed from H-18 to C-8, as expected.^18^ However, the ^4^*J*_CH_ couplings observed from H_3_-25 to C-19 and from H_3_-23 to C-5 appear to be of the same intensity as the other ^2^*J*_CH_ and ^3^*J*_CH_ couplings observed from H_3_-25 to C-17/C-16 and H_3_-23 to C-7/C-8, respectively. Despite the fact that ^4^*J*_CH_ HMBC correlations can be observed in conjugated systems (as noted with termicalcicolanone B)^22^ and in some rigid ring systems (such as yardenones A and B^23^ and alternarilactone A),^24^ they are typically less intense than ^2^*J*_CH_ and ^3^*J*_CH_ couplings present in those molecules. Thus, we hypothesized that some of the ^4^*J*_CH_ HMBC correlations proposed for wheldone^18^ likely corresponded to ^3^*J*_CH_ correlations. Further, reanalysis of the HMBC correlations of the 2,5-dihydrofuran ring suggested that the acrylic acid moiety was more likely to be at position C-5 than at position C-4. In a similar fashion, δ_C_ 157.3 was most likely to be in position C-18 rather than C-19, leading to potential improved assignment of ^3^*J*_CH_ HMBC correlations from H_3_-23 to δ_C_ 142.8 and from H_3_-25 to δ_C_ 157.3, respectively, instead of the ^4^*J*_CH_ HMBC correlations described previously. In summary, the evidence obtained from DFT calculations, coupled with the improbable ^4^*J*_CH_ HMBC correlations (Figure 1), suggested that the previously published structure was incorrect, pointing toward a different fused ring system and an alternate substitution pattern around the 2,5-dihydrofuran ring.

**Figure 2:**
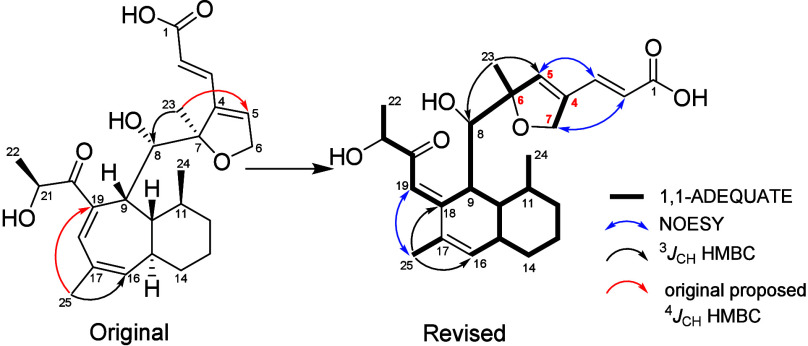
1,1-HD-ADEQUATE connectivities and key HMBC and NOESY correlations that supported the revised structure of wheldone. Note, the original proposed structure of wheldone was redrawn (left), so that some of the positions would align with the newly proposed structure (right). In addition, it was necessary to renumber some of the positions (noted in red) in the revised structure to fit the IUPAC rules.

**Figure 3:**
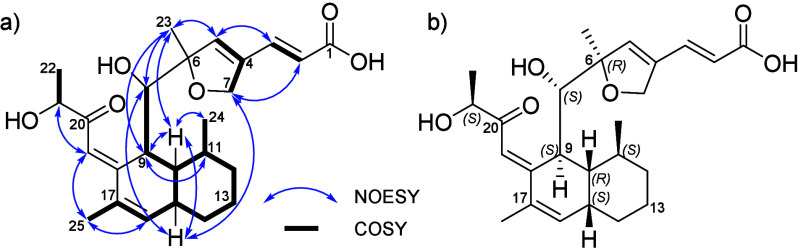
(a) Key COSY and additional NOESY correlations that supported the structure revision of wheldone. (b) Most probable configuration of wheldone based on DFT-GIAO calculations.

To elucidate the backbone of wheldone, particularly the fused ring system, a new set of 1D- and 2D-NMR experiments, including the 1,1-HD-ADEQUATE experiment,^19^ were carried out. Note, for the following discussion, the positions in the structure of wheldone have been renumbered for clarity (see Figure 2, Table S7). Analysis of 1,1-HD-ADEQUATE connectivity (^1^*J*_CC_) from δ_C_ 122.0 (C-19) to δ_C_ 208.7 (C-20) and δ_C_ 157.3 (C-18) suggested a bicyclo[4.4.0]dec-4-ene ring arrangement with an exocyclic 4-hydroxypent-1-en-3-one moiety instead of the bicyclo[5.4.0]undeca-3,5-diene proposed previously (Figure 2). This decalin-like ring system was supported by the observed ^3^*J*_CH_ HMBC correlations from δ_H_ 1.91 (H_3_-25) to δ_C_ 143.0 (C-16) and δ_C_ 157.3 (C-18) and the NOESY correlation observed between δ_H_ 1.91 (H_3_-25) and δ_H_ 6.57 (H-19), which indicates an *E-*configuration of the exocyclic double bond. A long-range 1,n-HD-ADEQUATE^19^ correlation was observed between δ_C_ 122.0 (C-19) and δ_C_ 74.2 (C-21), which provided additional evidence for the 4-hydroxypent-1-en-3-one moiety (Figure 2). Moreover, 1,1-HD-ADEQUATE connectivities showed attachments of δ_C_ 142.8 (C-5) to δ_C_ 95.2 (C-6) and of δ_C_ 74.8 (C-7) to δ_C_ 137.1 (C-4); these findings suggested that the acrylic acid moiety was positioned between the oxymethylene at δ_C_ 74.8 (C-7) and the vinylic carbon at δ_C_ 142.8 (C-5); this conclusion was supported by both the ^3^*J*_CH_ HMBC correlations from δ_H_ 1.42 (H_3_-23) to δ_C_ 76.1 (C-8) and δ_C_ 142.8 (C-5) and the NOESY correlations between δ_H_ 5.71 (H-2) and δ_H_ 4.76/4.82 (H_2_-7) and between δ_H_ 7.40 (H-3) and δ_H_ 6.45 (H-5) (Figure 3). This also indicated a *trans-*configuration of the acrylic acid moiety, as supported by the *J* value (16.0 Hz) between H-2 and H-3 (i.e., δ_H_ 7.40/δ_H_ 5.71). The bicyclic system was connected to the 2,5-dihydrofuran ring via HMBC correlations from δ_H_ 3.54 (H-8) to δ_C_ 95.2 (C-6) and δ_C_ 43.0 (C-9) and 1,1-HD-ADEQUATE connectivities between δ_C_ 95.2 (C-6), δ_C_ 76.1 (C-8), and δ_C_ 43.0 (C-9) (Figure 2); these assignments were supported by COSY correlations between δ_H_ 3.54 (H-8) and δ_H_ 3.85 (H-9) (Figure 3).

From the previous study,^18^ the configuration of position 21 was determined as *S* via Mosher’s ester analysis (confirmed via SCXRD analysis, *vide infra*). Using that information as a reference point, we could derive additional support for the revised structure of wheldone from the NOESY data. Of particular importance, the NOESY cross peaks observed between H-10 and H-15 indicated a *cis*-decalin-like fused ring. Also, NOESY correlations observed between H-9/H-10/H_3_-24 supported the *cis*-decalin conformation, and the equatorial orientation of H_3_-24 suggested either a 9*S*,10*R*,11*S*,15*S* or a 9*R*,10*S*,11*R*,15*R* configuration (Figure S12). Thus, the NOESY correlations observed between H-8 and H-5/H_2_-7/H-10/H-15/H_3_-23 and between H_3_-23 and H-5/H-8/H-9/H-10 were possible regardless of the configuration of positions 6 and 8, due to the free rotation of the C-6/C-8/C-9 bonds. Thus, analysis of the NOESY correlations, while inconclusive, permitted a reduction of the assignment of the absolute configuration of wheldone to eight possibilities (Table S15).

To explore these structural hypotheses, an exhaustive conformer search was performed by combining the results from five different conformer generators: Schrödinger MacroModel Monte Carlo Multiple Minima (version 2021-1), Schrödinger Confgenx (version 2021-1), OpenEye Omega Classic (version 3.1.2.2), OpenEye Omega Macrocycle (version 3.1.2.2), and MOE Low Mode MD (version 2020.09). Each conformer was minimized using the OPLS4^25^ force field implemented in MacroModel, and conformers less than a 10.0 kcal/mol cutoff were eliminated as were any redundant conformers based on atomic RMSD. This resulted in final ensembles of between 100 to 500 conformers for each test structure derived from the 1,1-HD-ADEQUATE and NOESY correlations. Conformers were optimized by using DFT calculations at the B3LYP/6-31+G(d,p) level of theory in the gas phase. Based on DFT methodologies reported by Pierens,^26^^1^H and ^13^C NMR chemical shifts were calculated at the WP04/aug-cc-pVZD and mPW1PW91/6-311+G(2d,p) levels of theory, respectively, with the IEFPCM in CH_3_OH. Comparisons between the calculated and experimental chemical shifts showed that the newly proposed decalin-like part of the structure of wheldone was a much better fit than prior structure proposals. Specifically, the experimental and calculated ^13^C NMR chemical shift values were in closer agreement in the decalin-like part of the structure compared to the originally proposed structure (see Table S12). Among the eight structure possibilities, DFT NMR chemical shift calculations for configuration 6*R,*8*S*,9*S*,10*R*,11*S*,15*S,*21*S* most closely matched the experimental data with a MAE of 0.09 and 2.4 ppm for the ^1^H and ^13^C NMR data, respectively (Table S15). In short, these data provided a possible, but not unequivocal, assignment of the configuration of the asymmetric centers in wheldone.

To unambiguously determine the structure and configuration of wheldone, we aimed to generate crystals suitable for SCXRD analysis. Initially, high-throughput crystallization techniques using ENaCt^20,27^ (encapsulated nanodroplet crystallization) were attempted, whereby 480 nanoscale parallel crystallization experiments were carried out using approximately 7 mg of isolated natural product. Unfortunately, only microcrystalline materials were observed and from only a limited number of ENaCt experiments (see Supporting Information). The introduction of brominated aromatic moieties into natural products is known to increase both crystallinity, through introduced intramolecular π-stacking interactions, and anomalous X-ray scattering, to assist in determination of absolute configuration.^28−31^ Thus, taking advantage of the carboxylic acid moiety in wheldone, a Steglich-type^32−34^ reaction was carried out using *p*-bromobenzylamine in dichloromethane with DCC and DMAP, leading to the formation of the wheldone *p*-bromobenzylamide derivative.^35,36^ HRESIMS analysis helped to establish the molecular formula of this derivative as C_32_H_40_O_5_NBr (*m*/*z* 598.2150, [M + H]^+^), which corresponded to an index of hydrogen deficiency of 13 and was consistent with the addition of a phenyl. Two doublets at δ_H_ 7.22 (*J* = 8.10 Hz, 2H) and δ_H_ 7.47 (*J* = 8.10 Hz, 2H), characteristic of a 1,4-disubstituted benzene, a singlet signal of a methylene at δ_H_/δ_C_ 4.41/43.6 ppm, and an amide carbonyl at δ_C_ 168.1, as well as HMBC correlations from δ_H_ 7.33/5.87/4.41 to δ_C_ 168.1 ppm, confirmed the position of the *p*-bromobenzylamide moiety (Table S8, Figures S18–S23). This compound was subjected to a series of classical crystallization experiments utilizing slow evaporation from nine different solvents. Microcrystalline solids were observed from CHCl_3_, THF, 2-MeTHF, EtOAc, and nitromethane but were unsuitable for SCXRD analysis. Based on those initial results, follow-up crystallizations by layered diffusion^37^ were carried out, with a 2-MeTHF/cycloheptane solvent system ultimately providing suitable single crystals for SCXRD analysis.

**Figure 4:**
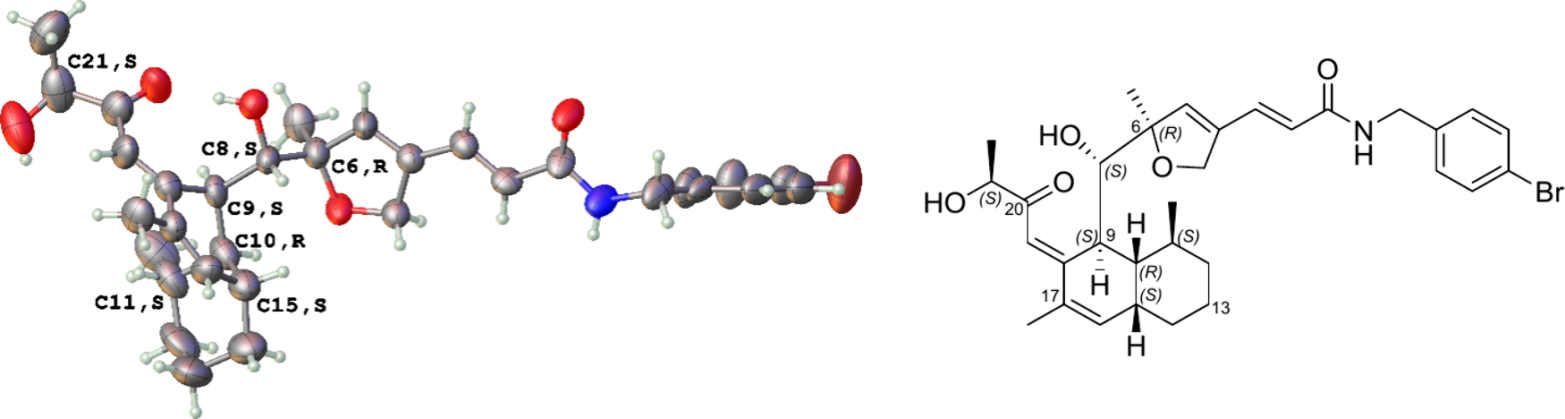
Structure of wheldone *p*-bromobenzylamide with the chiral centers labeled and assigned. The crystallographically independent, nondisordered molecule is shown, and anisotropic displacement parameters are displayed at 50%. Key: brown–bromine, red–oxygen, blue–nitrogen, gray–carbon, green–hydrogen. An additional perspective of the molecule is displayed in Figure S3.

Wheldone *p*-bromobenzylamide crystallized in the *P*1 space group as a 2-methyl tetrahydrofuran and cycloheptane solvate. Further analysis of the single-crystal structure solution (Figure 4; Table S5) illustrated agreement with the backbone proposed from the 1,1-HD-ADEQUATE experiment, showing unambiguously a *cis*-decalin-like ring system and the *E*-orientation of the exocyclic double bond in the 4-hydroxypent-1-en-3-one moiety. Moreover, based on the Flack parameter (−0.02(4), Parsons’ method), the absolute configuration was assigned as 6*R*,8*S*,9*S*,10*R*,11*S*,15*S*,21*S*; importantly, this was in agreement with the configuration of C-21 that was assigned previously by Mosher’s ester analysis^18^ and with the observed NOESY correlations (Figure 3). Additionally, this configuration agreed with the structure that yielded the highest accuracy for the ^1^H and ^13^C NMR chemical shift predictions. The structure also exhibited an S(8) intramolecular hydrogen bond between the hydroxy at C-8 and the ketone at C-20 in the solid state. An additional C(4) hydrogen bonding network was also observed between N-1 and O-1.^38^

Notably, the diffraction pattern of wheldone *p*-bromobenzylamide revealed a second set of much weaker reflections, indicative of incommensurate modulation within the structure; the modulation vector was able to be refined to q = (0,–0.004(8),0.2414(8)). Analysis of the modulation using the program Superflip^39,40^ revealed that the modulation is localized mainly in the solvent region and did not affect the main molecule. It would be very difficult to model the modulation of the solvent, and the impact on the structure description of the molecule would be minimal. Therefore, we proceeded with the structure analysis using only the main reflections and, consequently, the average structure, as detailed in the Supporting Information.

In summary, the structure of wheldone, a product of the competition of two fungal strains in coculture, has been revised, including the unequivocal assignment of the absolute configuration as 6*R*,8*S*,9*S*,10*R*,11*S*,15*S*,21*S*. To do so, a combination of orthogonal techniques was used, including working through computational data, NMR spectroscopy using the 1,1-HD-ADEQUATE experiment, appending a *p*-bromobenzyl moiety via semisynthesis to enhance crystallization properties, and finally, analyzing X-ray crystallographic data.

## Experimental Section

### General Experimental Procedures

Optical rotation data were obtained using a Rudolph Research Autopol III polarimeter, and UV spectra were measured with a Varian Cary 100 Bio UV–vis spectrophotometer. NMR data were collected using an Agilent 700 MHz NMR spectrometer equipped with a cryoprobe, operating at 700 MHz for ^1^H and 175 MHz for ^13^C, a Bruker AVANCE III 600 NMR spectrometer with a BCU-05 cooling unit with liquid N_2_ dewar, operating at 600 MHz for ^1^H and 150 MHz for ^13^C, and a Bruker Neo NMR spectrometer equipped with a H/F C/N TCI 5 mm Prodigy CryoProbe operating at a ^1^H observation frequency of 500 MHz for ^1^H and 125 MHz for ^13^C. In all cases, the NMR data were referenced to the residual solvent peaks for methanol-*d*_4_, specifically δ_H_/δ_C_ 3.31/49.0. HRMS data were acquired using a Thermo LTQ Orbitrap XL linear ion trap mass spectrometer equipped with a heated electrospray ionization source coupled to a Waters Acquity UPLC system by using an ACQUITY UPLC BEH C_18_ column (1.7 μm; 50 × 2.1 mm) set to 40 °C and a flow rate of 0.3 mL/min. The elution method consisted of a linear gradient of CH_3_CN-H_2_O (both acidified with 0.1% formic acid), starting at 15% CH_3_CN and holding it for 1 min, and then increasing linearly to 100% CH_3_CN over 8 min with a 1.5 min hold before returning to the starting conditions. Analytical and preparative HPLC analyses were performed with a Varian Prostar HPLC system equipped with two Prostar 210 pumps, a Prostar 701 fraction collector, a Prostar 335 photodiode array detector (PDA; Varian Inc.), and a SEDEX75 evaporative light scattering detector (ELSD; SEDERE Inc.) using either Phenomenex Synergi Max-RP C_12_ 80 Å analytical (4 μm; 250 × 4.6 mm) and preparative (4 μm; 250 × 21.2 mm) or Phenomenex Gemini–NX C_18_ 110 Å analytical (5 μm; 250 × 4.6 mm) and preparative (5 μm; 250 × 21.2 mm) columns. Data collection and analysis were carried out using Galaxie Chromatography Workstation software (version 1.9.3.2, Varian Inc.). Flash chromatography was performed on a Teledyne ISCO CombiFlash Rf 200 using various sizes of RediSep Rf GOLD silica gel columns and monitored by both ELSD and PDA detectors. Single-crystal X-ray diffraction analysis was performed using a Rigaku XtaLAB Synergy diffractometer equipped with a microfocus sealed Cu Kα X-ray tube (λ = 1.54184 Å) and a HyPix Arc-100 detector.

### Fungal Strains, Fermentation, and Isolation Procedures

*Aspergillus fischeri* (strain NRRL 181) was obtained from the ARS Culture Collection (NRRL), as noted previously.^41^*Xylaria flabelliformis* (strain G536)^17,18^ was isolated as an endophyte from surface sterilized twigs of *Asimina triloba* and identified using molecular methods, as detailed previously.^16^ These cultures were grown, first individually and then in coculture on Quaker breakfast oatmeal, essentially as described previously.^18^ To generate enough wheldone for the NMR spectroscopy and X-ray crystallography studies, the cocultures were grown in batches of 25 flasks each (i.e., each 250 mL Erlenmeyer flask containing 10 g of autoclaved oatmeal). The isolation and purification procedures were modified from the reported procedure,^18^ as will be detailed in a forthcoming manuscript, so as to yield ∼1 mg of wheldone per flask.

### Crystallization Experiments

Crystallization of wheldone was attempted using the encapsulated nanodroplet crystallization (ENaCt) methods.^20^ This proceeded through a series of experiments, as detailed in the Supporting Information. Additionally, a classical slow evaporation method was also attempted (see Supporting Information). Eventually, it was necessary to generate an analogue, wheldone, *p*-bromobenzylamide, as detailed below. The crystallization of this derivative was attempted by both classical slow evaporation and layered diffusion methods (see Supporting Information), the latter of which was successful. See “**X-ray Crystal Structure Analysis of the Wheldone*p*-bromobenzylamide Derivative**” in the Supporting Information for further details.

### Preparation of the Wheldone *p*-Bromobenzylamide Derivative

To a solution of wheldone (12.51 mg, 29.08 μmol, MW: 430.23 g/mol) and *p*-bromobenzylamine (29.92 mg, 161.75 μmol, MW: 184.98 g/mol) in CH_2_Cl_2_ (700 μL) at 0 °C was added a solution of dicyclohexylcarbodiimide (DCC, 20.18 mg, 97.88 μmol, MW: 206.18 g/mol) and 4-*N*,*N*-dimethylaminopyridine (DMAP, 13.23 mg, 108.29 μmol, MW: 122.17 g/mol) in cooled CH_2_Cl_2_ (750 μL). The mixture was stirred using a magnetic bar and kept under a nitrogen atmosphere at 0 °C in a water-ice bath for 8 h and then kept at room temperature for another 17 h. Next, the reaction mixture was dried under nitrogen, reconstituted in dioxane-CH_3_OH (1:1), and immediately fractionated using a Synergi Max-RP C_12_ column using a solvent system that started at 40:60 CH_3_CN-H_2_O (0.1% formic acid in both solvents) over 3 min, then 40:60 to 60:40 over 27 min, then 60:40 to 100:0 over 25 min, and finally 100:0 over 7 min at a flow rate of 21.2 mL/min and a Phenomenex Gemini–NX C_18_ column with a gradient system of 60:80 to 80:20 CH_3_OH-H_2_O (0.1% formic acid) over 30 min, then 80:20 to 100:0 over 10 min, and finally 100:0 over 7 min at a flow rate of 17.0 mL/min.

#### Wheldone (**1**)

White, amorphous solid. [α]_D_^20^ = +200° (*c* 0.05, CH_3_OH). UV (CH_3_OH) λ_Max_ (log ε) 236 (3.81), 300 (3.68) nm. ^1^H NMR (CD_3_OD 700 MHz) and ^13^C NMR (CD_3_OD, 175 MHz) (see Table S7); HRESIMS *m*/*z* 431.2415 [M + H]^+^ (calcd. for C_25_H_35_O_6_, 431.2428).

#### Wheldone *p*-Bromobenzylamide (**2**)

White solid. [α]_D_^20^ = +216° (*c* 0.05, CH_3_OH). UV (CH_3_OH) λ_Max_ (log ε): 225 (3.97), 273 (3.65), 307 (3.79) nm. ^1^H NMR (CD_3_OD, 700 MHz) and ^13^C NMR (CD_3_OD, 175 MHz) (see Table S8); HRESIMS *m*/*z* 598.2144 [M + H]^+^ (calcd. for C_32_H_41_NO_5_Br, 598.2163).

### Computational Details

3D models of the originally proposed wheldone structure were built using Spartan’10 software. Conformational analysis was performed using the MMFF94 molecular mechanics force field with the Monte Carlo search protocol. The resulting conformers under the 5 kcal/mol cutoff were checked for duplicates, and then geometry and frequency were optimized using the density-functional theory (DFT) method at the M06-2*X*/6-31+G(d,p) theory level in the gas phase. For the isotropic shielding tensor calculations, the gauge invariant atomic orbital (GIAO) method was used at B3LYP/6-311+G(2d,p) level of theory with the integral equation formalism-polarizable continuum model (IEFPCM) model in CH_3_OH. Upon acquisition of 1,1-HD-ADEQUATE and NOESY NMR data, conformer searches were carried out for possible structures of wheldone using the following programs: Schrödinger MacroModel Monte Carlo Multiple Minima (version 2021-1), Schrödinger Confgenx (version 2021-1), OpenEye Omega Classic (version 3.1.2.2), OpenEye Omega Macrocycle (version 3.1.2.2), and MOE Low Mode MD (version 2020.09). Conformers were minimized using the OPLS4^25^ force field in MacroModel, and higher energy conformers (>10.0 kcal/mol) were eliminated. Redundant conformers sharing equivalent energies were also removed. Conformer optimizations were carried out at the B3LYP/6-31+G(d,p) level of theory in the gas phase. ^1^H and ^13^C NMR chemical shifts were calculated at the WP04/aug-cc-pVZD and mPW1PW91/6-311+G(2d,p) levels of theory, respectively, with the IEFPCM in CH_3_OH, following methods reported by Pierens.^26^ The final ^1^H and ^13^C chemical shifts were calculated for each structure in accordance with the Boltzmann distribution and their relative energies. All calculations were performed employing the Gaussian’16 program software package.^42^

## Data Availability

The NMR data for wheldone have been deposited in the Natural Products Magnetic Resonance Database (NP-MRD; www.np_mrd.org) and can be found at NP0021048. Also, CCDC 2330603 contains the supplementary crystallographic data for this paper, and these data can be obtained free of charge via www.ccdc.cam.ac.uk/data_request/cif, or by emailing data_request@ccdc.cam.ac.uk, or by contacting The Cambridge Crystallographic Data Centre,12 Union Road, Cambridge CB2 1EZ, UK; fax:+441223336033.

## Supporting Information Available

Experimental procedures, characterization data, spectroscopic and crystallographic data (PDF)
Illustrated crystallographic information (CIF)
Illustrated crystallographic information (CIF)

## Supplementary Material

np4c00649_si_001.pdf

np4c00649_si_002.cif

np4c00649_si_003.cif
