# Supplementary material for: Wheldone Revisited: Structure Revision Via DFT-GIAO Chemical Shift Calculations, 1,1-HD-ADEQUATE NMR Spectroscopy, and X-ray Crystallography Studies
Source: J Nat Prod. 2024 Jul 23;87(8):2095–100. doi: 10.1021/acs.jnatprod.4c00649 (PMC11348420; doi:10.1021/acs.jnatprod.4c00649)
Supplement: Supplementary file 1 — np4c00649_si_001.pdf [file np4c00649_si_001.pdf]

SUPPORTING INFORMATION

Wheldone Revisited: Structure Revision via DFT-GIAO chemical shift calculations, 1,1-HD-ADEQUATE NMR Spectroscopy, and X-ray Crystallography Studies.

Manuel Rangel-Grimaldo,[1] Cody E. Earp,[1] Huzefa A. Raja,[1] Jared S. Wood,[2] Lina Mardiana,[3,4,5] Kin Lok Ho,[4] Alexandra Longcake,[4] R. Thomas Williamson,[2] Lukáš Palatinus,[6] Michael J. Hall,[4] Michael R. Probert,[4] Nicholas H. Oberlies[1]

[1]Department of Chemistry and Biochemistry, University of North Carolina at Greensboro, Greensboro, NC, 27402, USA.

[2]Department of Chemistry and Biochemistry, University of North Carolina Wilmington, Wilmington, NC, 28409, USA.

[3]Indicatrix Crystallography Ltd, Newcastle University, Newcastle upon Tyne, Tyne and Wear, NE1 7RU, UK.

[4]Chemistry – School of Natural and Environmental Sciences, Newcastle University, Newcastle upon Tyne, Tyne and Wear, NE1 7RU, UK.

[5]Department of Chemistry, Universitas Indonesia, Depok, Jawa Barat, 16424, Indonesia.

[6]Department of Structure Analysis, Institute of Physics of the Czech Academy of Sciences, Na Slovance 2, Prague, Czechia

Table of Contents

EXPERIMENTAL SECTION **S1**

Crystallization Experiments **S1**

X-ray Crystal Structure Analysis of the Wheldone *p*-bromobenzylamide Derivative **S10**

Table S1. Stock solutions of wheldone prepared for ENaCt experiments. **S1**

Table S2. Oils used for ENaCt experiments. **S1**

Table S3. Crystallization of wheldone *p*-bromobenzylamide by slow evaporation. **S8**

Table S4. Crystallization of wheldone *p*-bromobenzylamide by solvent-solvent diffusion. **S9**

Table S5. Crystallographic tables for wheldone *p*-bromobenzylamide. **S14**

Table S6. Comparison of the previously reported ^{1}H (700 MHz) and ^{13}C (175 MHz) NMR data of wheldone and the isolated wheldone in this work. **S15**

Table S7. ^{1}H- (700 MHz), ^{13}C- (175 MHz) NMR data for wheldone in CD_3OD. **S16**

Table S8. ^{1}H- (700 MHz) and ^{13}C- (175 MHz) NMR data for wheldone *p*-bromobenzylamide in CD_3OD. **S17**

Table S9. Comparison of the experimental and calculated ^{1}H- and ^{13}C-NMR chemical shifts based on the original publication of the wheldone structure, with the numbering as shown in the structure below. **S18**

Table S10. Coordinates of the significant conformers of the orignal proposed structure of wheldone. **S19**

Table S11. Total Gibbs Free Energy and Boltzmann distribution of the significant conformers (>1%) conformers of the orignal proposed structure of wheldone. **S25**

Table S12. Comparison of the experimental and calculated ^{1}H- and ^{13}C-NMR chemical shifts of the revised structure of wheldone, using the numbering scheme in the drawing below. **S26**

Table S13. Coordinates of the significant conformers of the revised structure of wheldone. **S27**

Table S14. Total Gibbs Free Energy and Boltzmann distribution of the significant conformers (>1%) conformers of the revised structure of wheldone. **S28**

Table S15. MAE and RMSD for all possible configurations tested. **S29**

Figure S1. The asymmetric unit of wheldone *p*-bromobenzylamide with the solvent molecules modelled; only the major disorder component is shown and anisotropic displacement parameters are given at 50%. **S12**

Figure S2. The asymmetric unit of wheldone *p*-bromobenzylamide with a solvent mask applied; only the major disorder component is shown and anisotropic displacement parameters are given at 50%. **S12**

Figure S3. The structure of wheldone p-bromobenzylamide shown from different perspectives. Only the major disorder component of one crystallographically independent molecule is shown. Anisotropic displacement parameters are given at 50 %. Key: brown – bromine, red – oxygen, blue – nitrogen, grey – carbon, green – hydrogen. **S13**

Figure S4. 3D representation of wheldone and key NOESY correlations. **S28**

Figure S5. ^{1}H NMR spectra comparison between wheldone (**a**) and wheldone *p*-bromobenzylamide (**b**) in CD_3OD (700 MHz). **S31**

Figure S6. ^{1}H NMR spectrum of wheldone in CD_3OD (700 MHz). **S32**

Figure S7. ^{13}C NMR spectrum of wheldone in CD_3OD (175 MHz). **S33**

Figure S8. 1,1-HD-ADEQUATE NMR spectrum of wheldone in CD_3OD. **S34**

Figure S9. gHSQCAD spectrum of wheldone in CD_3OD. **S35**

Figure S10. gHMBCAD spectrum of wheldone in CD_3OD. **S36**

Figure S11. gCOSY spectrum of wheldone in CD_3OD. **S37**

Figure S12. NOESY spectrum of wheldone in CD_3OD. **S38**

Figure S13. 1D NOE spectrum of wheldone (excitation of H-8) in CD_3OD. **S39**

Figure S14. 1D NOE spectrum of wheldone (excitation of H-9) in CD_3OD. **S40**

Figure S15. 1D NOE spectrum of wheldone (excitation of H-10) in CD_3OD. **S41**

Figure S16. 1D NOE spectrum of wheldone (excitation of H-15) in CD_3OD. **S42**

Figure S17. 1D NOE spectrum of wheldone (excitation of H-24) in CD_3OD. **S43**

Figure S18. ^{1}H NMR spectrum of wheldone *p*-bromobenzylamide in CD_3OD (700 MHz). **S44**

Figure S19. ^{13}C NMR spectrum of wheldone *p*-bromobenzylamide in CD_3OD (175 MHz). **S45**

Figure S20. gHSQCAD spectrum of wheldone *p*-bromobenzylamide in CD_3OD. **S46**

Figure S21. gHMBCAD spectra of wheldone *p*-bromobenzylamide in CD_3OD. **S47**

Figure S22. gCOSY spectra of wheldone *p*-bromobenzylamide in CD_3OD. **S48**

Figure S23. NOESY spectra of wheldone *p*-bromobenzylamide in CD_3OD. **S49**

References **S50**

EXPERIMENTAL SECTION

Crystallization Experiments

ENaCt First Round – Crystallization Screening

Crystallization of wheldone was attempted using the encapsulated nanodroplet crystallization (ENaCt) methods.[1] To do so, 7 mg of wheldone was dissolved in 800 μL of MeOH, and 100 μL of this stock solution was transferred into 8x screw top vials. The solvent was allowed to evaporate under ambient conditions overnight, yielding aliquots of approximately 0.9 mg each.

To prepare vials 1 to 8, solvent was added in increments starting from 6 µL, until the sample had just dissolved or until a total volume of 192 µL was reached, at which point the supernatant was used. Following step-up of initial ENaCt experiments, the remaining material from vials 3 to 8 was recovered, pooled, split into 4 further vials and the solvent allowed to evaporate overnight. Then to prepare vials 9 to 12, as before, solvent was added in increments starting from 6 µL, until the sample had just dissolved or until a total volume of 192 µL was reached, at which point the supernatant was used.

Table S1. Stock solutions of wheldone prepared for ENaCt experiments.

Vial	Solvent	Volume of solvent (µL)
1	DMSO	12
2	DMF	6
3	MeOH	12
4	1,1,1,3,3,3-Hexafluoroisopropanol (HFIP)	12
5	Toluene	192[a]
6	1,2-Dichloroethane (DCE)	48
7	2-MeTHF	24
8	Methyl *tert*-butyl ether (MTBE) [a]	192[a]
9	EtOAc	12
10	MeCN	12
11	Methyl *iso*-butyl ketone [a]	192[a]
12	Nitromethane (NM)[a]	192[a]

[a] supernatant used for further experiments.

Table S2. Oils used for ENaCt experiments.

PDMSO	poly(dimethylsiloxane); CAS: 63148-62-9; supplier: Sigma Aldrich
FC-40	Fluorinert FC-40; CAS: 51142-49-5; supplier: Fluorochem
FY	Fomblin YR-1800; CAS: 69991-67-9; supplier: Alfa Aesar
MO	Mineral oil; CAS: 8042-47-5; supplier: Sigma Aldrich

ENaCt "Standard Method"

Using an SPT Labtech Mosquito liquid-handling robot, 200 nL of one of four oils (PDMSO, FC-40, FY and MO) was dispensed into each well of a 96-well SWISSCI LCP plate with 100-micron spacer. Next, 50 nL of stock solution containing wheldone was dispensed into the oil droplets within each well. The plates were sealed with a glass cover slip and were stored in the dark at room temperature for 2 weeks prior to analysis.

ENaCt End Point Analysis

After 14 days, the 96 well plates were examined by cross-polarised optical microscopy, and the result of each ENaCt experiment was classified as: **F**: Fail; **1**: remains in solution; **2**: oiled-out or non-crystalline solid; **3**: micro-crystalline solid; **4**: crystals suitable for X-ray diffraction analysis.

ENaCt First Round - Plate Layout and Crystallization Outcomes

**P1**														
**Vial**	**Standard Method**		**Volume of Oil = 200 nL**											
	**Volume of Solvent**		**50 nL**											
	**Solvents**		**1**	**2**	**3**	**4**	**5**	**6**	**7**	**8**	**9**	**10**	**11**	**12**
**1**	DMSO	**A**	No oil	PDMSO					No oil	FY				
	DMSO	**B**	No oil	FC-40					No oil	MO				
**2**	DMF	**C**	No oil	PDMSO					No oil	FY				
	DMF	**D**	No oil	FC-40					No oil	MO				
**3**	MeOH	**E**	No oil	PDMSO					No oil	FY				
	MeOH	**F**	No oil	FC-40					No oil	MO				
**4**	HFIP	**G**	No oil	PDMSO					No oil	FY				
	HFIP	**H**	No oil	FC-40					No oil	MO				

**P1**														
**Vial**	**Standard Method**		**Volume of Oil = 200 nL**											
	**Volume of Solvent**		**50 nL**											
	**Solvents**		**1**	**2**	**3**	**4**	**5**	**6**	**7**	**8**	**9**	**10**	**11**	**12**
**1**	DMSO	**A**	2	1	1	1	1	1	1	1	1	1	1	1
	DMSO	**B**	1	1	1	1	1	1	1	1	1	1	1	1
**2**	DMF	**C**	2	2	2	1	1	1	2	1	1	1	1	1
	DMF	**D**	F	1	1	1	1	1	2	1	1	1	1	1
**3**	MeOH	**E**	2	2	2	2	2	F	F	1	1	1	1	1
	MeOH	**F**	1	2	2	2	2	2	F	1	1	1	1	1
**4**	HFIP	**G**	2	2	2	2	2	2	F	1	1	1	1	1
	HFIP	**H**	2	2	2	2	2	2	F	1	1	1	1	1

P2														
**Vial**	**Standard Method**		**Volume of Oil = 200 nL**											
	**Volume of Solvent**		**50 nL**											
	**Solvents**		**1**	**2**	**3**	**4**	**5**	**6**	**7**	**8**	**9**	**10**	**11**	**12**
**5**	Tol	**A**	No oil	PDMSO					No oil	FY				
	Tol	**B**	No oil	FC-40					No oil	MO				
**6**	DCE	**C**	No oil	PDMSO					No oil	FY				
	DCE	**D**	No oil	FC-40					No oil	MO				
**7**	2-MeTHF	**E**	No oil	PDMSO					No oil	FY				
	2-MeTHF	**F**	No oil	FC-40					No oil	MO				
**8**	MTBE	**G**	No oil	PDMSO					No oil	FY				
	MTBE	**H**	No oil	FC-40					No oil	MO				

P2														
**Vial**	**Standard Method**		**Volume of Oil = 200 nL**											
	**Volume of Solvent**		**50 nL**											
	**Solvents**		**1**	**2**	**3**	**4**	**5**	**6**	**7**	**8**	**9**	**10**	**11**	**12**
**5**	Tol	**A**	2	F	2	2	1	2	2	1	1	1	1	1
	Tol	**B**	2	1	1	1	1	1	2	1	1	1	1	1
**6**	DCE	**C**	2	2	2	2	2	2	2	2	1	1	1	2
	DCE	**D**	2	2	2	1	1	2	2	2	2	2	2	2
**7**	2-MeTHF	**E**	2	2	F	F	F	F	F	1	1	1	1	1
	2-MeTHF	**F**	2	2	1	1	1	1	F	1	1	1	1	1
**8**	MTBE	**G**	2	2	2	2	1	2	F	1	1	1	1	1
	MTBE	**H**	2	1	1	1	1	1	2	1	1	1	1	2

P3														
**Vial**	**Standard Method**		**Volume of Oil = 200 nL**											
	**Volume of Solvent**		**50 nL**											
	**Solvents**		**1**	**2**	**3**	**4**	**5**	**6**	**7**	**8**	**9**	**10**	**11**	**12**
**9**	EtOAc	**A**	No oil	PDMSO					No oil	FY				
	EtOAc	**B**	No oil	FC-40					No oil	MO				
**10**	MeCN	**C**	No oil	PDMSO					No oil	FY				
	MeCN	**D**	No oil	FC-40					No oil	MO				
**11**	MIBK	**E**	No oil	PDMSO					No oil	FY				
	MIBK	**F**	No oil	FC-40					No oil	MO				
**12**	NM	**G**	No oil	PDMSO					No oil	FY				
	NM	**H**	No oil	FC-40					No oil	MO				

P3														
**Vial**	**Standard Method**		**Volume of Oil = 200 nL**											
	**Volume of Solvent**		**50 nL**											
	**Solvents**		**1**	**2**	**3**	**4**	**5**	**6**	**7**	**8**	**9**	**10**	**11**	**12**
**9**	EtOAc	**A**	2	2	2	2	2	1	2	1	1	1	1	1
	EtOAc	**B**	2	2	2	2	2	2	2	1	1	1	2	1
**10**	MeCN	**C**	2	2	2	2	2	2	2	2	2	2	2	2
	MeCN	**D**	2	2	1	1	1	1	2	2	2	2	2	2
**11**	MIBK	**E**	F	1	2	2	2	2	F	1	1	1	1	1
	MIBK	**F**	F	1	1	1	1	1	F	1	1	1	1	1
**12**	NM	**G**	2	2	2	2	2	2	2	2	2	1	2	2
	NM	**H**	2	1	1	1	1	1	2	1	1	1	1	1

ENaCt First Round - Results

No crystalline solids were observed, with experiments only giving amorphous material not suitable for SCXRD analysis.

ENaCt Second Round – Crystallization Screening with Secondary Solvents

A stock solution was prepared by dissolving wheldone (9.65 mg) in MeOH (96 µL), giving concentration of 100.5 mg/mL. This was then used to set up the following ENaCt experiments

ENaCt "Secondary Solvent Method" (SSM)

Using an SPT Labtech Mosquito liquid-handling robot, 250 nL of one of 48 high boiling point solvents was dispensed into each well of a 96-well SWISSCI LCP plate with 100-micron spacer. Next, 50 nL of stock solution containing wheldone was dispensed into the solvent droplet within each well. The plates were sealed with a glass cover slip and were stored in the dark at room temperature for 2 weeks prior to analysis.

ENaCt Second Round - Plate Layout and Crystallization Outcomes

P4														
**Vial**	**SSM**		**Volume of high BP Solvent = 250nL**											
	**Volume of Stock Solution**		**50 nL**											
	**Stock Solutions**		**1**	**2**	**3**	**4**	**5**	**6**	**7**	**8**	**9**	**10**	**11**	**12**
**13**	Wheldone in MeOH	**A**	DMSO				NO_2Ph				2-MeO(C_2H_4)OAc			
		**B**	DESO				*n*-PrCN				2-Ph(C_2H_4)OAc			
		**C**	DMF				*n*-BuCN				$MeNO_2$			
		**D**	FMD				*i*-BuCN				$HexNO_2$			
		**E**	DiiPrF				PhCN				1,4-Dioxane			
		**F**	DinBuF				*n*-PrOAc				$CPNO_2$			
		**G**	Toluene				*n*-BuOAc				$DMSO_3$			
		**H**	MIBK				*i*-BuOAc				$DESO_3$			

P4														
**Vial**	**SSM**		**Volume of high BP Solvent = 250 nL**											
	**Volume of Stock Solution**		**50 nL**											
	**Stock Solutions**		**1**	**2**	**3**	**4**	**5**	**6**	**7**	**8**	**9**	**10**	**11**	**12**
13	Wheldone in MeOH	A	2	2	2	2	2	2	2	2	2	2	2	2
		B	2	1	1	2	2	2	2	2	2	2	2	2
		C	2	2	2	2	2	2	2	2	2	2	2	2
		D	2	2	2	2	2	2	2	2	2	2	2	2
		E	3	3	3	3	3	3	3	3	2	2	2	2
		F	2	2	2	2	2	2	2	2	2	2	2	2
		G	2	2	2	2	2	2	2	2	2	2	2	2
		H	2	2	2	2	2	2	2	2	2	2	2	2

P5														
**Vial**	**SSM**		**Volume of high BP Solvent = 250 nL**											
	**Volume of Stock Solution**		**50 nL**											
	**Stock Solutions**		**1**	**2**	**3**	**4**	**5**	**6**	**7**	**8**	**9**	**10**	**11**	**12**
**13**	Wheldone in MeOH	**A**	ES				Ethylene glycol				NMP			
		**B**	DMC				MPD				THTP-1-oxide			
		**C**	*n*-Butanol				H_2O				NMF			
		**D**	*n*-Hexanol				Chlorobenzene				Methoxybenzene			
		**E**	*n*-Octanol				*p*-fluorotoluene				HDN			
		**F**	2,2,2-TCE				*p*-Xylene				NMI			
		**G**	2,2,2-TFE				2-Methylpyridine				1-Formylpyrrolidine			
		**H**	2-nitroethanol				4-Formylmorpholine				DMAN			

P5														
Vial	SSM		Volume of high BP Solvent = 250 nL											
	Volume of Stock Solution		50 nL											
	Stock Solutions		1	2	3	4	5	6	7	8	9	10	11	12
13	Wheldone in MeOH	A	3	3	3	3	2	2	2	2	1	1	1	1
		B	2	2	2	2	2	2	2	2	2	2	2	2
		C	2	2	2	2	3	3	3	3	2	2	2	2
		D	2	2	2	2	2	2	2	2	2	2	2	2
		E	2	2	2	2	2	2	2	2	2	2	2	2
		F	2	2	2	2	2	2	2	2	3	2	1	3
		G	2	2	2	2	2	2	2	2	2	2	2	2
		H	1	1	1	1	1	1	1	1	2	2	2	2

ENaCt Second Round - Selected Images of Crystallization Outcomes

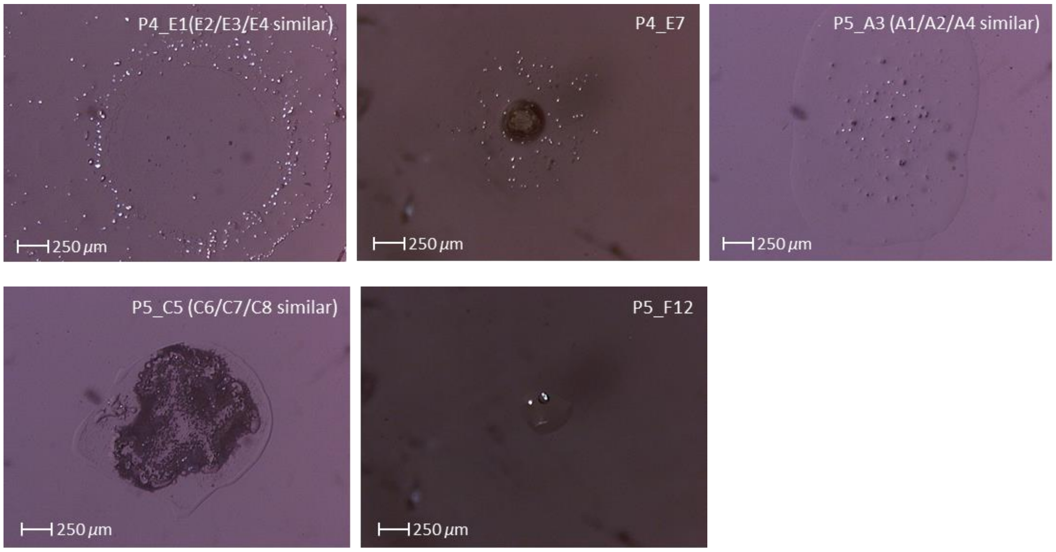

ENaCt Second Round - Results

Microcrystalline solids were observed in wells from MeOH stock solutions mixed with the following secondary solvents: Di*i*PrF (P4_E1/E2/E3/E4), PhCN (P4_E5/E6/E7/E8), ES (P5_A1/A2/A3/A4), H_2O (P5_C5/C6/C7/C8), and NMI (P5-F9/F12). However, these crystalline materials were very small and not suitable for SCXRD analysis.

Crystallization of Wheldone by Classical Slow Evaporation Methods

Wheldone was recovered previous ENaCt experiments, dissolved in MeOH and split equally into 5x screw top vials. The MeOH was allowed to evaporate under ambient conditions overnight. Then solvents were added (MeOH, DMSO, Di*i*PrF, PhCN, and NMI) until the sample had just dissolved. Solvents were chosen based on the results of previous ENaCt screening. The vials were then closed and stored under ambient conditions, in the dark. Following slow evaporation of the solvent, crystallization outcomes were assessed over time by cross polarized optical microscopy.

Slow Evaporation Crystallization - Selected Images of Crystallization Outcomes

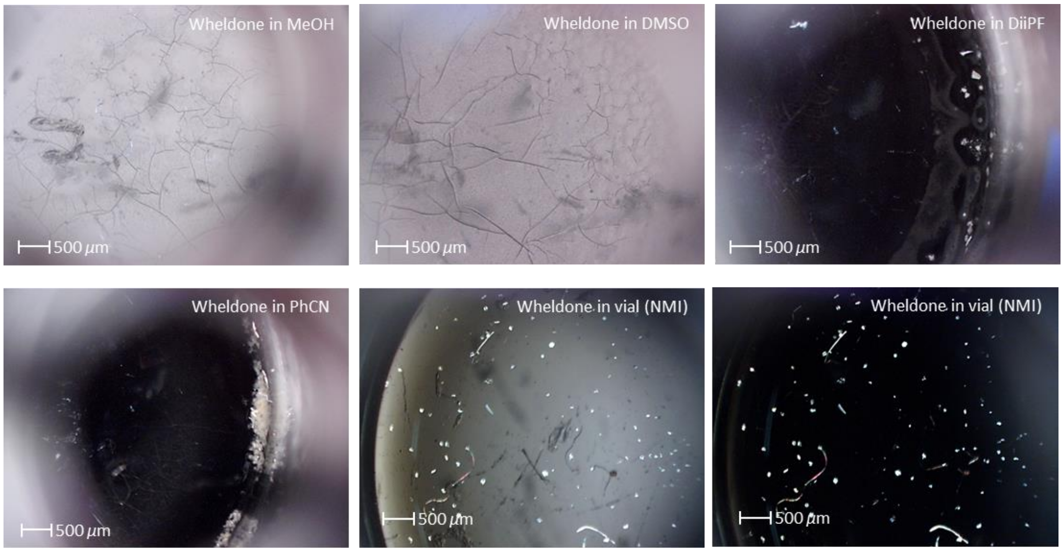

Slow Evaporation Crystallization – Results

Microcrystalline solids were observed from Di*i*PrF, PhCN, and NMI. However, crystalline solids observed were very small and not suitable for SCXRD analysis.

Crystallization of Wheldone *p*-bromobenzylamide by Classical Slow Evaporation Methods

4.78 mg of wheldone *p*-bromobenzylamide was dissolved in 900 μL of MeOH, and 100 μL of this stock solution was transferred into 9 x 400-µL flat bottom glass insert vials (Agilent Technologies), followed by placing insert vials into 9 x 2-mL screw top vials (Agilent Technologies), labelled vials 1-9. The solvent was allowed to evaporate under ambient conditions overnight. Into each 400-µL flat bottom glass insert vial, solvent was added portion-wise until the sample had just dissolved. Samples were then closed with a 9-mm blue screw cap PTFE/RS, stored under ambient conditions in the dark for 4 days, and assessed over time by cross polarized optical microscopy.

After assessment of the crystallization outcomes, any remaining solvent was allowed to evaporate and samples were recycled through the addition of a new solvent, and the crystallization experiments were repeated to give vials 1a-7a. Finally, vial 2a, 6, 8 and 9 were pooled and evaporated to give vial 10, and new solvent added, and crystallization repeated.

Table S3. Crystallization of wheldone *p*-bromobenzylamide by slow evaporation.

**Crystallization Results – Slow Evaporation**				
Vial	Sample mass (mg)	Solvent	Crystalline solids obtained (Y/N)	Volume of solvent added (µL)
1	0.53	MeOH	N	24
2	0.53	$CHCl_3$	Y	24
3	0.53	2-MeTHF	Y	24
4	0.53	EtOAc	Y	24
5	0.53	MeCN	N	36
6	0.53	NM	Y	24
7	0.53	1,4-Dioxane	N	24
8	0.53	MIBK	N	24
9	0.53	PhCl	N	36
1a	0.53	IPA	N	24
2a	0.53	DCM	N	24
3a	0.53	THF	Y	24
4a	0.53	*i*-PrOAc	N	24
5a	0.53	DCE	N	36
7a	0.53	*n*-$PentNO_2$	N	36
10	2.12	NM	N	200

Slow Evaporation Crystallization Results

Microcrystalline solids were observed from a number of solvents; however, they were very small and not suitable for SCXRD analysis.

Crystallization of Wheldone *p*-bromobenzylamide by Classical Layered Diffusion Methods

The recovered samples of wheldone *p*-bromobenzylamide from previous slow evaporation experiments, in 400-µL flat bottom glass inserts, were redissolved in minimal solvent followed by slow addition of an anti-solvent, so as to form two discreet layers. The glass inserts containing samples were then placed in 2-mL screw top vials and closed with a 9-mm blue screw cap PTFE/RS, stored under ambient conditions in the dark for 4 days, and assessed over time by cross polarized optical microscopy.

Table S4. Crystallization of wheldone *p*-bromobenzylamide by solvent-solvent diffusion.

**Crystallization Results – Solvent-solvent diffusion**				
Vial	Sample mass (mg)	Solvent / (µL)	Antisolvent / (µL)	Crystalline solids (Y/N)
11 (previously from 1a)	0.53	MeOH/ 40	Cyclohexane/ 40	N
12 (previously from 3a)	0.53	THF/ 30	Cycloheptane/ 30	Y
13 (previously from 5a)	0.53	$CHCl_3$/ 30	Cyclohexane/ 40	N
12a (previously from 12)	0.53	2-MeTHF/ 30	Cycloheptane/ 30	Y

Layered Diffusion Crystallization Results

Large crystals were observed from layered diffusion experiments using 2-MeTHF/cycloheptane (vial 12a), and these were analyzed by SCXRD.

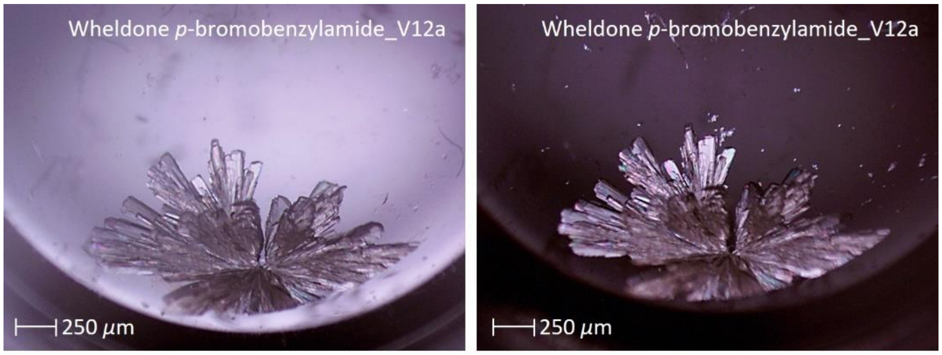

X-ray Crystal Structure Analysis of the Wheldone *p*-bromobenzylamide Derivative.

A colorless crystal (0.046 × 0.126 × 0.308 mm) was analyzed on a Rigaku XtaLAB Synergy diffractometer equipped with a micro-focus sealed Cu Kα X-ray tube radiation and a HyPix Arc-100 detector. The sample was extracted under the inert oil Fomblin-YR1800 from a microscope slide then mounted on a 35 µm MiTeGen loop before being flash cooled to 150 K using an Oxford Cryosystems CryostreamPlus open-flow N_2 cooling device. Unit cell measurement, data collection and data reduction were performed using the software CrysAlisPRO (Oxford Diffraction /Agilent Technologies UK Ltd., Yarnton, England). A numerical absorption correction was applied using the Gaussian integration over a multi-faceted crystal model.

The raw diffraction images of wheldone *p*-bromobenzylamide revealed a set of much weaker reflections, indicative of incommensurate modulation. A complete description of the structure would thus also require the determination of the modulation. The modulation vector refined to q = (0,-0.004(8),0.2414(8)). This is very close to the commensurate value of 0.25c*. However, the value 0.2414(8) is significantly different from 0.25 at the confidence level of 11σ, and the modulation is thus more likely to be incommensurate, with a four-fold superstructure being a very good approximation.

The basic structure is triclinic, space group $P1$; thus, the only possible superspace group is $P1(\alpha\beta\gamma)$. The modulation vector's first two coordinates are thus not fixed to 0 by symmetry but only by the nature of the modulation. The prominent feature of the structure is that it is quasi-layered, with well-ordered layers of the wheldone *p*-bromobenzylamide molecules separated by the more disordered regions of the solvent. The layers alternate along *c* and the modulation vector along *c** indicates that the modulation involves small changes in the structure of the consecutive layers along *c*. Another interesting feature is the almost complete absence of the first-order satellites in the layer *0kl*. Such a feature is typical for systematic absences, but no systematic absences are associated with triclinic superspace groups. This indicates an unusual feature of the modulation: in the projection onto the *b**-*c** plane, the modulation in two layers separated by a phase shift of 0.5 appears the same. In the superstructure description, this would mean an apparent *c*-glide-like feature, although the structure as a whole does not have this symmetry.

An attempt was made to determine and refine the modulated structure. The structure was solved directly in superspace by the program Superflip.[2,3] This method provides a superspace electron

density, which allows an *ab initio* determination of both the average structure and its modulation. The solution was successful, and the complete wheldone *p*-bromobenzylamide molecule could be located in the solution. However, the features in the superspace density did not reveal any interpretable modulation of the molecule. It appears that the modulation is localized mainly in the disordered solvent region, especially on the cycloheptane molecules, and it does not affect, or only minimally affects, the wheldone *p*-bromobenzylamide molecule. The modulation is thus, in all likelihood, caused by only partial ordering of the disordered solvent, and because the solvent region is challenging to model already in the average structure, attempts to model the modulation of solvent were doomed to failure. Nevertheless, the whole analysis confirms that ignoring the modulation has no impact on the description of the main molecule, its structure, connectivity, or absolute structure determination.

Because the modulation was impossible to properly treat, the average structure was instead reduced and refined, although the authors acknowledge the presence of incommensurate modulation in the data. The structure was solved using SHELXT[4] and refined using SHELXL[5] through the Olex2[6] interface. Crystallographic data for wheldone *p*-bromobenzylamide (either with the solvent molecules modelled or masked by a solvent mask, respectively) have been deposited with the Cambridge Crystallographic Data Centre (CCDC) with the codes CCDC 2330602 and CCDC 2330603. Full refinement details can be found within the relevant Crystallographic Information Files (CIFs) in the fields '_diffrn_special_details' and '_refine_special_details'.

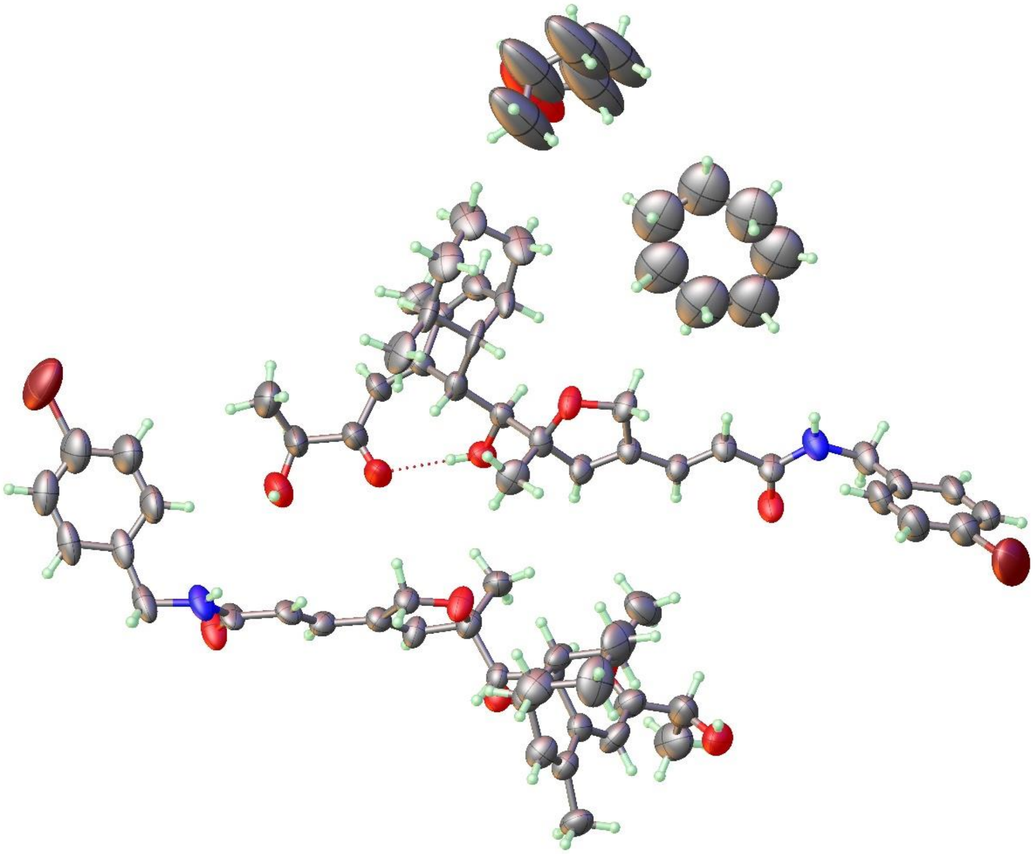

Figure S1. The asymmetric unit of wheldone *p*-bromobenzylamide with the solvent molecules modelled; only the major disorder component is shown, and anisotropic displacement parameters are given at 50%. Key: brown – bromine, red – oxygen, blue – nitrogen, grey – carbon, green – hydrogen.

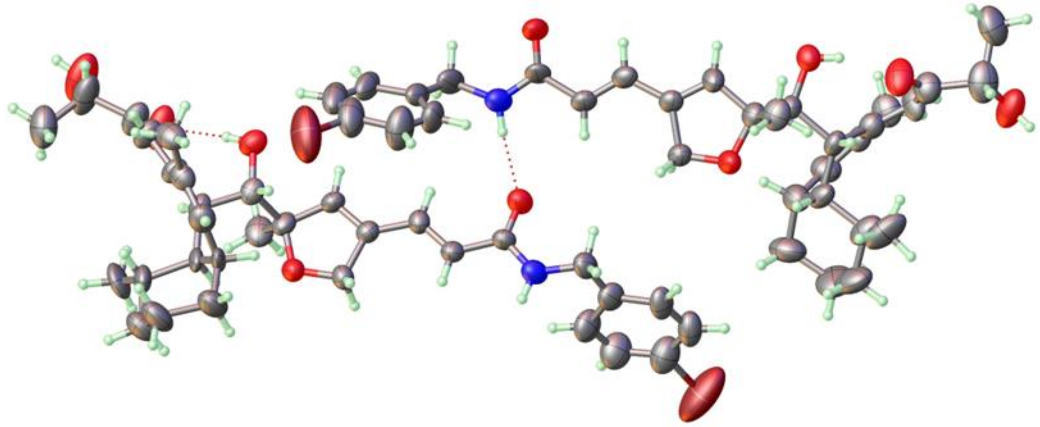

Figure S2. The asymmetric unit of wheldone *p*-bromobenzylamide with a solvent mask applied; only the major disorder component is shown, and anisotropic displacement parameters are given at 50%. Key: brown – bromine, red – oxygen, blue – nitrogen, grey – carbon, green – hydrogen.

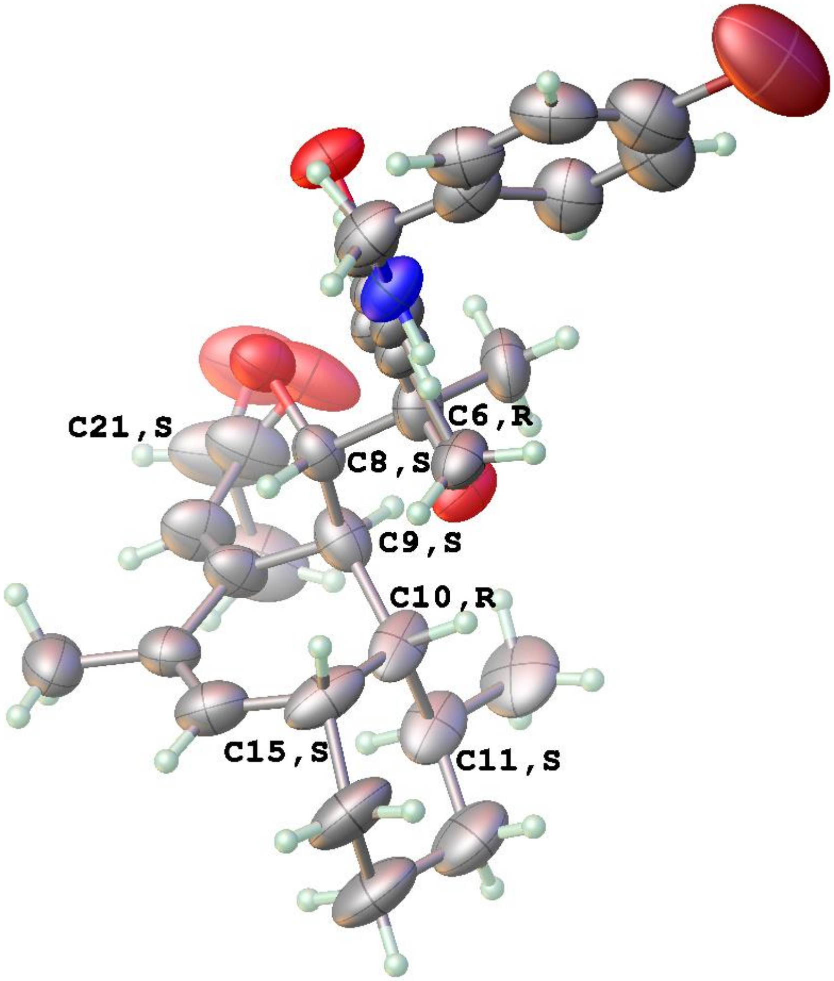

Figure S3. The structure of wheldone p-bromobenzylamide shown from a different perspective. Only the major disorder component of one crystallographically independent molecule is shown. Anisotropic displacement parameters are given at 50%. Key: brown – bromine, red – oxygen, blue – nitrogen, grey – carbon, green – hydrogen.

Table S5. Crystallographic tables for wheldone *p*-bromobenzylamide.

**Identification code**	CCDC 2330602	CCDC 2330603
**Empirical formula**	$C_{38}H_{52}BrNO_{5.5}$	$C_{32}H_{40}BrNO_5$
**Formula weight**	690.71	598.56
**Temperature/K**	149.99(10)	149.99(10)
**Crystal system**	triclinic	triclinic
**Space group**	P1	P1
***a*/Å**	9.2578(3)	9.2578(3)
***b*/Å**	14.0193(4)	14.0193(4)
***c*/Å**	14.8024(7)	14.8024(7)
***α*/°**	107.647(3)	107.647(3)
***β*/°**	99.007(4)	99.007(4)
***γ*/°**	90.433(2)	90.433(2)
**Volume/$Å^3$**	1805.10(12)	1805.10(12)
Z	2	2
**$ρ_{calc}$g/cm^3**	1.271	1.101
**μ/mm^{-1}**	1.894	1.817
**F(000)**	732.0	628.0
**Crystal size/mm^3**	0.308 × 0.126 × 0.046	0.308 × 0.126 × 0.046
**Radiation**	Cu Kα (λ = 1.54184)	Cu Kα (λ = 1.54184)
**2Θ range for data collection/°**	6.354 to 133.19	6.354 to 133.19
**Index ranges**	-10 ≤ h ≤ 11, -16 ≤ k ≤ 16, -17 ≤ l ≤ 17	-10 ≤ h ≤ 11, -16 ≤ k ≤ 16, -17 ≤ l ≤ 17
**Reflections collected**	45063	45063
**Independent reflections**	11884 [R_{int} = 0.0640, R_{sigma} = 0.0501]	11884 [R_{int} = 0.0640, R_{sigma} = 0.0501]
**Data/restraints/parameters**	11884/1878/1076	11884/1194/842
**Goodness-of-fit on F^2**	1.316	1.067
**Final R indexes [I>=2σ (I)]**	R_1 = 0.1149, wR_2 = 0.3133	R_1 = 0.0929, wR_2 = 0.2596
**Final R indexes [all data]**	R_1 = 0.1330, wR_2 = 0.3286	R_1 = 0.1074, wR_2 = 0.2736
**Largest diff. peak/hole / e $Å^{-3}$**	1.28/-0.76	0.51/-0.54
**Flack parameter (classical)**	-0.07(4)	-0.08(4)
**Flack parameter (Parsons' method)**	-0.03(4)	-0.02(4)

Table S6. Comparison of the previously reported ^{1}H (700 MHz) and ^{13}C (175 MHz) NMR data of wheldone[7] and the isolated wheldone in this work.

	**Original paper**			**This Paper**		
**pos**	**δC,**	**type**	**δH (mult, *J* in Hz)**	**δC,**	**type**	**δH (mult, *J* in Hz)**
1	170.2[†]	C		170.3	C	
2	122.6	CH	5.71 (d, 15.97)	122.7	CH	5.71 (d, 16.00)
3	137.4	CH	7.40 (d, 16.00)	137.4	CH	7.40 (d, 16.01)
4	137.1	C		137.1	C	
5	142.8	CH	6.46 (t, 1.96)	142.8	CH	6.45 (t, 2.02)
6	74.8	CH_2	4.75 (dd, 12.01, 1.30)	74.8	CH_2	4.76 (dd, 12.08, 1.34)
			4.81 (dd, 12.01, 1.03)			4.82 (dd, 12.08, 1.10)
7	95.2	C		95.2	C	
8	76.1	CH	3.54 (d, 10.41)	76.1	CH	3.54 (d, 10.47)
9	43.0	CH	3.84 (dd, 10.45, 2.12)	43.0	CH	3.85 (dd, 10.46, 2.06)
10	44.0	CH	1.96 (dt, 10.78, 3.49)	44.0	CH	1.96 (dt, 10.84, 3.46)
11	31.6	CH	1.19 (m)	31.6	CH	1.22 (q, 12.47)
12	36.4	CH_2	0.94 (dq, 12.82, 2.63)	36.4	CH_2	0.95 (dq, 12.85, 2.47)
			1.56 (m)			1.57 (d, 12.95)
13	23.5	CH_2	1.23 (m)	23.5	CH_2	1.22 (m)
			1.48 (m)			1.48 (m)
14	32.8	CH_2	1.50 (m)	32.8	CH_2	1.50 (m)
			1.74 (m)			1.79 (d, 13.19)
15	34.6	CH	2.88 (br s)	34.6	CH	2.89 (br s)
16	142.9	CH	5.91 (br s)*	143.0	CH	5.92 (br s)
17	133.6	C		133.6	C	
18	121.9	CH	6.57 (s)	122.0	CH	6.57 (s)
19	157.3	C		157.3	C	
20	208.7	C		208.7	C	
21	74.2	CH	4.41 (q, 7.04)	74.2	CH	4.42 (q, 7.02)
22	20.8	CH_3	1.35 (d, 7.04)	20.8	CH_3	1.35 (d, 7.04)
23	19.3	CH_3	1.42 (s)	19.3	CH_3	1.42 (s)
24	20.5	CH_3	0.78 (d, 6.47)	20.5	CH_3	0.79 (d, 6.50)
25	20.0	CH_3	1.91 (d, 1.11)	20.0	CH_3	1.91 (d, 2.34)

*There was a typographical error in the original publication, where this was noted as δ_H 5.81. Upon re-examining the spectra, we note that this should have been reported as δ_H 5.91. †There was a typographical error in the original publication, where this was noted as δ_C 172.2. Upon re-examining the spectra, we note that this should have been reported as δ_C 170.2. These data are organized based on the numbering proposed in the original publication (as shown below).

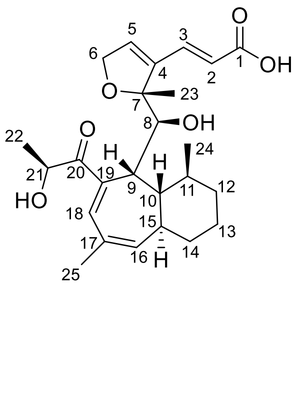

Table S7. ^{1}H (700 MHz), ^{13}C (175 MHz) NMR data for wheldone in CD_3OD (Note: this uses the newly proposed structure and numeration).

pos	*δ*C,	type	*δ*H (mult, *J* in Hz)
1	170.3	C	
2	122.7	CH	5.71 (d, 16.00)
3	137.4	CH	7.40 (d, 16.01)
4	137.1	C	
5	142.8	CH	6.45 (t, 2.02)
6	95.2	C	
7	74.8	CH_2	4.76 (dd, 12.08, 1.34)
			4.82 (dd, 12.08, 1.10)
8	76.1	CH	3.54 (d, 10.47)
9	43.0	CH	3.85 (dd, 10.46, 2.06)
10	44.0	CH	1.96 (dt, 10.84, 3.46)
11	31.6	CH	1.22 (q, 12.47)
12	36.4	CH_2	0.95 (dq, 12.85, 2.47)
			1.57 (d, 12.95)
13	23.5	CH_2	1.22 (m)
			1.48 (m)
14	32.8	CH_2	1.50 (m)
			1.79 (d, 13.19)
15	34.6	CH	2.89 (br s)
16	143.0	CH	5.92 (br s)
17	133.6	C	
18	157.3	C	
19	122.0	CH	6.57 (s)
20	208.7	C	
21	74.2	CH	4.42 (q, 7.02)
22	20.8	CH_3	1.35 (d, 7.04)
23	19.3	CH_3	1.42 (s)
24	20.5	CH_3	0.79 (d, 6.50)
25	20.0	CH_3	1.91 (d, 2.34)

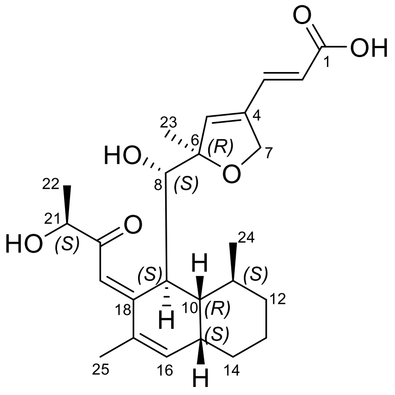

Table S8. ^{1}H (700 MHz) and ^{13}C (175 MHz) NMR data for wheldone *p*-bromobenzylamide in CD_3OD.

pos	*δ*C,	type	*δ*H (mult, *J* in Hz)
1	168.1	C	
2	124.1	CH	5.87 (d, 15.80)
3	133.8	CH	7.33 (d, 15.80)
4	135.5	C	
5	141.8	CH	6.42 (s)
6	95.3	C	
7	74.9	CH_2	4.74 (dd, 11.99, 1.54)
			4.81 (dd, 11.94, 1.15)
8	76.1	CH	3.53 (d, 10.39)
9	43.0	CH	3.84 (dd, 10.34, 2.52)
10	44.1	CH	1.95 (dt, 11.52, 3.26)
11	31.6	CH	1.22 (m)
12	36.4	CH_2	0.93 (dq, 13.07, 2.76)
			1.56 (d, 13.24)
13	23.5	CH_2	1.22 (m)
			1.46 (m)
14	32.8	CH_2	1.49 (m)
			1.78 (d, 13.20)
15	34.6	CH	2.88 (br s)
16	142.8	CH	5.91 (br s)
17	133.7	C	
18	157.3	C	
19	122.0	CH	6.57 (s)
20	208.7	C	
21	74.2	CH	4.42 (q, 7.01)
22	20.7	CH_3	1.35 (d, 7.01)
23	19.4	CH_3	1.41 (s)
24	20.4	CH_3	0.78 (d, 6.37)
25	20.0	CH_3	1.90 (d, 2.34)
1'		NH	8.53 (br s)
2'	43.6	CH_2	4.41 (s)
3'	139.2	C	
4'/8'	130.6	CH	7.22 (d, 8.10)
5'/7'	132.7	CH	7.47 (d, 8.10)
6'	122.0	C	

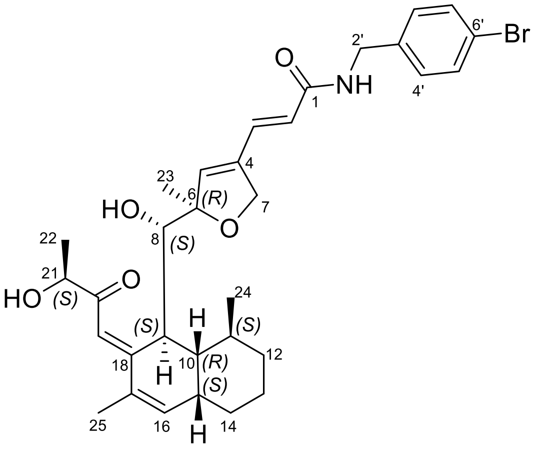

Table S9. Comparison of the experimental and calculated ^{1}H- and ^{13}C-NMR chemical shifts based on the original publication of the wheldone structure,[7] with the numbering as shown in the structure below.

Position	Assigned ^{13}C Chemical Shift (ppm)	Boltzmann-Weighted Predicted ^{13}C Chemical Shift (ppm)	Δ ^{13}C Chemical Shift (ppm)	Assigned ^{1}H Chemical Shift (ppm)	Boltzmann-Weighted Predicted ^{1}H Chemical Shift (ppm)	Δ ^{1}H Chemical Shift (ppm)
**1**	170.2†	166.0	4.2			
**2**	122.6	115.9	6.7	5.71	5.38	0.33
**3**	137.4	143.7	-6.3	7.40	7.13	0.27
**4**	137.1	139.5	-2.4			
**5**	142.8	145.6	-2.8	6.46	6.17	0.29
**6**	74.8	74.1	0.7	4.75	4.63	0.12
				4.81	4.83	-0.02
**7**	95.2	91.4	3.8			
**8**	76.1	71.0	5.1	3.54	3.93	-0.39
**9**	43.0	37.4	5.6	3.84	3.50	0.34
**10**	44.0	53.1	-9.1	1.96	0.92	1.04
**11**	31.6	38.4	-6.8	1.19	1.72	-0.53
**12**	36.4	36.3	0.1	0.94	0.88	0.06
				1.56	1.53	0.03
**13**	23.5	27.5	-4.0	1.23	1.43	-0.20
				1.48	1.65	-0.17
**14**	32.8	34.9	-2.1	1.50	0.96	0.54
				1.74	1.85	-0.11
**15**	34.6	44.9	-10.3	2.88	2.78	0.10
**16**	142.9	148.2	-5.3	5.91*	6.14	-0.23
**17**	133.6	128.9	4.7			
**18**	121.9	143.4	-21.5	6.57	6.48	0.09
**19**	157.3	138.0	19.3			
**20**	208.7	201.0	7.7			
**21**	74.2	67.0	7.2	4.41	4.59	-0.18
**22**	20.8	22.5	-1.7	1.35	1.22	0.13
**23**	19.3	22.4	-3.1	1.42	1.39	0.03
**24**	20.5	19.1	1.4	0.78	1.06	-0.28
**25**	20.0	25.4	-5.4	1.91	1.89	0.02

12 Conformers used to weight Boltzmann populations. The 12 conformers make up a predicted total of 96.4%. No imaginary frequencies were present after optimization.

^{13}C Mean Absolute Error: 5.9. Optimization: Gas phase, M062X/6-31+G(d,p), Chemical shift prediction: PCM MeOH, B3LYP/6-311+G(2d,p).

^{1}H Mean Absolute Error: 0.24 Optimization: Gas phase, M062X/6-31+G(d,p), Chemical shift prediction: PCM MeOH, B3LYP/6-311+G(2d,p).

*There was a typographical error in the original publication, where this was noted as δ_H 5.81. Upon re-examining the spectra, we note that this should have been reported as δ_H 5.91. †There was a typographical error in the original publication, where this was noted as δ_C 172.2. Upon re-examining the spectra, we note that this should have been reported as δ_C 170.2.

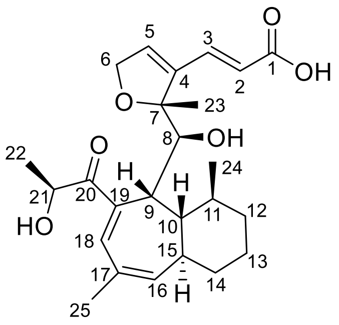

Table S10. Coordinates of the significant conformers of the orignal proposed structure of wheldone.

	Conformer 1			Conformer 3		
atom	x	y	z	x	y	z
C1	-0.1313939427	-6.1534712098	1.0894262474	-0.1278062532	-6.1033332082	1.0588570911
C2	-0.1594486179	-4.8915549548	1.5713815640	-0.1911755479	-4.8369337172	1.5233690694
C3	-0.7474121566	-3.6962917620	0.9572566473	-0.8232009111	-3.6667606972	0.9028354203
C4	-1.7779584377	-3.6872685131	0.0880034759	-1.8549341521	-3.7012854482	0.0357314963
C5	-2.6345103892	-4.7820424269	-0.4876537907	-2.6800070941	-4.8272613151	-0.5255702590
C6	-2.3003840154	-6.2356813523	-0.1103063240	-2.3085852163	-6.2618484055	-0.1147662299
C7	-0.7779467652	-6.5544150576	-0.2176668973	-0.7807718419	-6.5450446154	-0.2304307989
C8	-4.1079672592	-4.4718884856	-0.1481642127	-4.1607086726	-4.5474765830	-0.1911857664
C9	-5.0575351003	-5.4452377229	-0.8419588444	-5.0883887046	-5.5641575707	-0.8512021817
C10	-4.6937181379	-6.8905504644	-0.5141210431	-4.6856040950	-6.9897811859	-0.4853926257
C11	-3.2344471416	-7.2142088593	-0.8615633712	-3.2209230666	-7.2813548088	-0.8366944105
C12	0.3487341617	-7.2500980553	1.9682625274	0.4126226927	-7.1763516051	1.9371166677
C13	1.0042308616	-6.9691858948	3.3321282920	1.0778356486	-6.8637050116	3.2893147529
O14	1.0723904721	-8.1764176156	4.0632998787	1.2188027302	-8.0537736865	4.0230600072
C15	2.4084789235	-6.3821204419	3.1842946540	2.4536586412	-6.2180315323	3.1112458483
O16	0.2028077357	-8.4274042337	1.6525744643	0.3146466902	-8.3570278134	1.6252221792
C17	-0.1342809612	-2.3950174537	1.4272355422	-0.2584320827	-2.3398996620	1.3610824167
C18	-0.1049720079	-5.9473864148	-1.4725175023	-0.1450758300	-5.9503815587	-1.5095149929
C19	1.2793527753	-6.5570990981	-1.8133729000	1.2581195294	-6.5161262801	-1.8492104702
C20	2.2881462256	-6.6226969418	-0.6779282412	2.2860579199	-6.5173487166	-0.7296102288
C21	2.5273785034	-7.9056607216	-0.3695259088	2.5775209270	-7.7829645152	-0.3983244005
C22	1.6989428102	-8.8157004597	-1.2128845484	1.7639255859	-8.7416676837	-1.2043482452
O23	1.0395519506	-7.9444958847	-2.1332582832	1.0603378680	-7.9170719864	-2.1291765271
C24	2.9909021033	-5.5100078905	-0.0635532725	2.9522943391	-5.3590052445	-0.1563713181
C25	2.7637997864	-4.1979965068	-0.2395566285	2.6188615041	-4.0701204079	-0.3307857691
C26	3.6051197765	-3.2223747588	0.4789275445	3.3647979158	-2.9668474666	0.3008071453
O27	3.3104706939	-1.9590436429	0.1339640464	4.4045361600	-3.3731600537	1.0622817092
O28	4.4708412878	-3.4908624682	1.2920720131	3.0840847422	-1.7954218787	0.1714905473
C29	1.8721752961	-5.8937702227	-3.0574609315	1.8092838529	-5.8634668978	-3.1176728593
O30	-0.9334818135	-6.1442675137	-2.6115916293	-0.9753801733	-6.2167249700	-2.6291294901
C31	-2.9395418606	-8.6809215633	-0.5359303403	-2.8785753565	-8.7310113642	-0.4830958022
H32	-2.5519612843	-6.3613264191	0.9547744442	-2.5480311318	-6.3673629027	0.9554656128
H33	-0.6636328970	-7.6374270464	-0.3024526158	-0.6408316216	-7.6266876479	-0.2909145208
H34	-2.5419674149	-4.6955523681	-1.5819408385	-2.5849906053	-4.7657131038	-1.6220568743
H35	0.3102951672	-4.6913761883	2.5320285144	0.2878475028	-4.6127449670	2.4748122820
H36	-2.0963579084	-2.6981904380	-0.2472825729	-2.2053658412	-2.7255198381	-0.3071155262
H37	-4.2381737602	-4.5386287083	0.9412812307	-4.2849134449	-4.5824003979	0.9003668447
H38	-4.3415149478	-3.4408730553	-0.4372402249	-4.4235705993	-3.5316453676	-0.5086554388
H39	-6.0916956624	-5.2343995150	-0.5483768920	-6.1261883117	-5.3707811157	-0.5583482698
H40	-4.9956109256	-5.2945919669	-1.9284523132	-5.0364808728	-5.4435579229	-1.9417755020
H41	-4.8495818794	-7.0738898878	0.5601688338	-4.8312973002	-7.1496956753	0.5940727092
H42	-5.3555558810	-7.5791182579	-1.0526183690	-5.3333253346	-7.7091838186	-1.0000827032
H43	-3.1060004225	-7.0577135254	-1.9404721851	-3.1042046316	-7.1389369521	-1.9186259614
H44	0.3676384995	-6.2780083490	3.8935020801	0.4213035634	-6.1902705574	3.8541204212
H45	0.8621704093	-8.8911199463	3.4387931107	0.9644817653	-8.7808652345	3.4321799692
H46	2.8351803527	-6.2418026592	4.1798889229	2.8994573565	-6.0754739652	4.0980936010
H47	2.4033394453	-5.4199726565	2.6673064936	2.4099297958	-5.2562811189	2.5945332477
H48	3.0446657189	-7.0792020849	2.6314884057	3.1000877676	-6.8947167464	2.5442063429
H49	0.9348474980	-2.3559115099	1.1909249863	-0.7970649153	-1.5035939640	0.9108997466
H50	-0.6193283837	-1.5361833932	0.9584072462	0.7990258666	-2.2363107329	1.0955165910
H51	-0.2277141493	-2.2953927898	2.5144176546	-0.3336056123	-2.2454000914	2.4506317474
H52	-0.0011398076	-4.8637242983	-1.3668326867	-0.0870091869	-4.8602182373	-1.4377760413
H53	3.2227570823	-8.2599822876	0.3842201652	3.2953955369	-8.0939658384	0.3539018603
H54	0.9621888322	-9.3676489843	-0.6135923864	1.0602305688	-9.3018411468	-0.5732188654
H55	2.3073639119	-9.5365179123	-1.7711333088	2.3853368588	-9.4545498030	-1.7605479338
H56	3.7967763870	-5.7868468401	0.6147401125	3.8066898731	-5.5800056503	0.4796033520
H57	1.9882309252	-3.7984207733	-0.8850339229	1.7743137146	-3.7473772668	-0.9309895086
H58	3.8885005385	-1.3551744893	0.6291904067	4.8224255768	-2.5741843776	1.4169691752
H59	1.2113661934	-6.0556692735	-3.9109764396	1.9348353307	-4.7851615391	-2.9899389360
H60	1.9973651397	-4.8170281509	-2.9167799939	2.7802616555	-6.3037514783	-3.3583406049
H61	2.8485485245	-6.3362497510	-3.2730345575	1.1215597872	-6.0349054585	-3.9467348389
H62	-0.8930920309	-7.0891678792	-2.8270352234	-0.8694632371	-7.1588721920	-2.8308985086
H63	-1.9918844641	-9.0276226247	-0.9589409332	-3.6594840961	-9.4046072624	-0.8495368918
H64	-3.7304834835	-9.3229659174	-0.9370513912	-2.8039973266	-8.8617343169	0.6036369852
H65	-2.9006994182	-8.8350238211	0.5501867632	-1.9317802814	-9.0628017188	-0.9194253004

Table S10. Coordinates of the significant conformers of the orignal proposed structure of wheldone (*cont.*).

	Conformer 2			Conformer 4		
atom	x	y	z	x	y	z
C1	-0.0129608519	-5.8117895390	1.1145230127	-0.0390878687	-5.7905224840	1.0837354205
C2	-0.0309373422	-4.5096185337	1.4620226000	-0.0999241499	-4.4805495674	1.3978096894
C3	-0.6989783919	-3.3871835184	0.7944011055	-0.8258998487	-3.3989741050	0.7234919889
C4	-1.7610455550	-3.4716154144	-0.0298582643	-1.8908698832	-3.5429796104	-0.0892711322
C5	-2.6080771796	-4.6231072235	-0.4973338437	-2.6979661139	-4.7349750719	-0.5254845483
C6	-2.2448360683	-6.0420117935	-0.0233412680	-2.2903466408	-6.1231416656	-0.0001486326
C7	-0.7257073046	-6.3602335193	-0.1030675012	-0.7635415396	-6.4020761276	-0.0968603962
C8	-4.0790293233	-4.3199064160	-0.1391607822	-4.1777950467	-4.4606868498	-0.1809272736
C9	-5.0325588539	-5.3504825021	-0.7392882817	-5.1012707059	-5.5418123047	-0.7360839538
C10	-4.6263989470	-6.7704930739	-0.3533305270	-4.6567715098	-6.9289845043	-0.2810151348
C11	-3.1684348707	-7.0684586805	-0.7270925365	-3.1927280569	-7.2052313555	-0.6465806178
C12	0.7394844598	-6.7092022119	2.0405030510	0.7868594030	-6.6285692915	2.0014446116
C13	0.3020497293	-8.1649757481	2.2332207715	0.4348101640	-8.1018697834	2.2301037233
O14	1.3425397495	-8.8957217987	2.8306763906	1.5307323652	-8.7677237268	2.8041910101
C15	-0.9619267268	-8.1721960486	3.0995582608	-0.8002945020	-8.1578221030	3.1349813777
O16	1.6547594360	-6.3042171787	2.7415414397	1.6996068976	-6.1636049085	2.6694761127
C17	-0.0937234421	-2.0384781926	1.1177111216	-0.2800882478	-2.0189569426	1.0215144103
C18	-0.0489932540	-5.8779139621	-1.4151388298	-0.1342048947	-5.9530783065	-1.4429361821
C19	1.2870789043	-6.6077360532	-1.7270597226	1.2321365601	-6.6251027288	-1.7460311533
C20	2.2609268352	-6.7766538342	-0.5710993567	2.2513320705	-6.6594785312	-0.6184505623
C21	2.3816413524	-8.0801402234	-0.2774959891	2.4982640856	-7.9336268820	-0.2798339926
C22	1.5375894278	-8.9080857983	-1.1908719028	1.7007570585	-8.8692287559	-1.1287266630
O23	0.9074631857	-7.9649900196	-2.0494408603	0.9181784794	-8.0199472282	-1.9593077057
C24	3.0396764941	-5.7344177537	0.0822254264	2.9599197111	-5.5262188677	-0.0402912823
C25	2.9131012587	-4.4114528898	-0.0891775871	2.7166247032	-4.2297922938	-0.2815660829
C26	3.7993192918	-3.5012221644	0.6665027842	3.4671452062	-3.1446581932	0.3771260842
O27	3.4703619247	-2.2051153528	0.4604465790	4.5067849877	-3.5696067302	1.1217173119
O28	4.7150788217	-3.8285929892	1.3843796792	3.1885162173	-1.9694294090	0.2704126686
C29	1.9569476694	-6.0036124597	-2.9609082014	1.8363579068	-6.0682197997	-3.0354684229
O30	-0.8988790721	-6.0797357911	-2.5308252645	-0.9909919831	-6.2590418232	-2.5297698741
C31	-2.8301020522	-8.5249139394	-0.3986448815	-2.8159280258	-8.6350656539	-0.2495587610
H32	-2.4982199239	-6.0983826540	1.0474738047	-2.5246515809	-6.1413671829	1.0766941524
H33	-0.6187692301	-7.4470817535	-0.1018919537	-0.6251218228	-7.4847897794	-0.0612379554
H34	-2.5400067598	-4.6236525319	-1.5966964260	-2.6272030505	-4.7707999363	-1.6244721876
H35	0.5588979519	-4.2433661223	2.3393371167	0.5044703541	-4.1692542421	2.2500315773
H36	-2.1164519458	-2.5192305029	-0.4287940451	-2.2883983142	-2.6127109127	-0.5002145626
H37	-4.1765123831	-4.3115165031	0.9556174522	-4.2773205898	-4.4097291361	0.9125905585
H38	-4.3426558356	-3.3148391212	-0.4882067814	-4.4684979804	-3.4785946008	-0.5713131171
H39	-6.0594100107	-5.1448731405	-0.4177930715	-6.1346389269	-5.3490309703	-0.4277724691
H40	-5.0172077001	-5.2589868745	-1.8337936877	-5.0830787646	-5.5016620802	-1.8336231549
H41	-4.7520099943	-6.9115226374	0.7313935686	-4.7760467528	-7.0179474798	0.8099477041
H42	-5.2858187061	-7.4964286328	-0.8435809478	-5.2968254355	-7.6967203895	-0.7316944601
H43	-3.0697256317	-6.9235770910	-1.8108610021	-3.1034575613	-7.1105537445	-1.7361946468
H44	0.0824179756	-8.6399510981	1.2727350767	0.2115524212	-8.6045742397	1.2849623981
H45	1.8833240700	-8.2512172023	3.3164370563	2.0442262160	-8.0889963518	3.2723130704
H46	-1.7831863784	-7.6338838417	2.6156503228	-1.6567896870	-7.6593760260	2.6701457690
H47	-0.7520706454	-7.6965693727	4.0624922940	-0.5836664385	-7.6629608509	4.0866903624
H48	-1.2696342359	-9.2048452297	3.2777700884	-1.0584143224	-9.2007051784	3.3314770501
H49	0.9605660341	-1.9983999945	0.8209246742	0.7617403813	-1.9218908799	0.6956309349
H50	-0.6255605953	-1.2317697152	0.6086208988	-0.8680871181	-1.2445058771	0.5242574364
H51	-0.1326300327	-1.8484834035	2.1960887509	-0.3000181304	-1.8238765020	2.0997596386
H52	0.1263079766	-4.7987573126	-1.3642103472	-0.0189292397	-4.8646459120	-1.4516496101
H53	3.0092182487	-8.4995243426	0.5022050378	3.1903782851	-8.2645848712	0.4875399785
H54	0.7779715267	-9.4983174319	-0.6569785209	1.0429661526	-9.5296304903	-0.5461312318
H55	2.1398289361	-9.6050571129	-1.7886166422	2.3473614711	-9.5058780695	-1.7485962829
H56	3.7912558799	-6.0727454180	0.7935055808	3.7435461808	-5.7785284170	0.6692499072
H57	2.1718047125	-3.9555409036	-0.7377756150	1.9263832201	-3.8831473534	-0.9397222314
H58	4.0902676089	-1.6675341776	0.9766357862	4.9069814394	-2.7838458405	1.5227953078
H59	1.2646518333	-6.0312708957	-3.8039895074	1.1201406816	-6.1695622421	-3.8523767128
H60	2.2505938029	-4.9656279620	-2.7859663864	2.0957254660	-5.0120726175	-2.9274693552
H61	2.8506590397	-6.5818006778	-3.2088516837	2.7444034647	-6.6256269153	-3.2801654776
H62	-0.8563351175	-7.0238511582	-2.7457270812	-0.9124885182	-7.2120502247	-2.6870326081
H63	-1.8853249254	-8.8503640156	-0.8463387095	-1.8628535529	-8.9583569882	-0.6808698436
H64	-3.6120139613	-9.1892501523	-0.7797660589	-3.5801808589	-9.3366961061	-0.5983638405
H65	-2.7643859288	-8.6817687682	0.6859047661	-2.7473699269	-8.7382123636	0.8409569196

Table S10. Coordinates of the significant conformers of the orignal proposed structure of wheldone (*cont.*).

	Conformer 14			Conformer 7		
atom	x	y	z	x	y	z
C1	-0.0673565264	-5.8724086449	1.0524701251	-0.0932010438	-5.8335110691	1.0107360817
C2	-0.0615462497	-4.5593340106	1.3586394677	-0.1241494720	-4.5121664967	1.2812688618
C3	-0.7426581213	-3.4489592885	0.6856633534	-0.8611689906	-3.4424225410	0.6020055912
C4	-1.8170191882	-3.5382332607	-0.1215559754	-1.9525049550	-3.5922557631	-0.1732435588
C5	-2.6908475657	-4.6887543956	-0.5392370227	-2.7942340760	-4.7827700746	-0.5417442208
C6	-2.3672055583	-6.0845417889	0.0230323164	-2.4115410898	-6.1506059471	0.0518848581
C7	-0.8613938124	-6.4621623917	-0.0931611163	-0.8971384048	-6.4812702890	-0.0965971324
C8	-4.1515656642	-4.3076154379	-0.2158832497	-4.2599726518	-4.4400093935	-0.1994227696
C9	-5.1421944086	-5.3397441115	-0.7446162534	-5.2258094269	-5.5181836748	-0.6788269605
C10	-4.7902075135	-6.7346449524	-0.2387602030	-4.8192305204	-6.8839117804	-0.1362546304
C11	-3.3474237608	-7.1214321500	-0.5887921705	-3.3719442627	-7.2368642775	-0.5032305953
C12	0.7901827238	-6.7386500467	1.9172083316	0.8145130790	-6.6463081058	1.8745842788
C13	0.5540574713	-8.2494884239	1.9679499778	0.6478781853	-8.1653899640	1.9552623051
O14	1.6971485759	-8.8933104906	2.4714662263	1.8314466832	-8.7484552611	2.4392544694
C15	-0.6738248024	-8.5007865898	2.8491929712	-0.5444228049	-8.4573396706	2.8715267254
O16	1.6472242481	-6.2809341746	2.6596534758	1.6644774864	-6.1400205512	2.5942884447
C17	-0.1143313844	-2.1007545544	0.9639853062	-0.2787369893	-2.0649256336	0.8328351482
C18	-0.2409632733	-6.1023874898	-1.4666802959	-0.3279022572	-6.1363871929	-1.4963553315
C19	1.1640068785	-6.6970731454	-1.7156561364	1.0905319572	-6.6850815941	-1.7724015899
C20	2.2359505065	-6.4004336108	-0.6793759608	2.1763891747	-6.3411503638	-0.7668961375
C21	2.7972077347	-7.5543388993	-0.2948619897	2.7848260357	-7.4706722511	-0.3821536843
C22	2.1444163628	-8.7042630479	-0.9992014411	2.1529377661	-8.6507454374	-1.0548889479
O23	1.0136047651	-8.1256022407	-1.6478987979	0.9913477766	-8.1176167434	-1.6869536038
C24	2.6397344069	-5.0382353498	-0.3386433417	2.5226149516	-4.9625946681	-0.4353792765
C25	3.4583803153	-4.7159818613	0.6726668442	3.3814107752	-4.5909771113	0.5228537609
C26	3.8828552355	-3.3325891885	0.9537637970	3.6263108552	-3.1543200915	0.7575586137
O27	3.3307150084	-2.4096124668	0.1294851277	4.5627121838	-2.9518436049	1.7037038787
O28	4.6605088204	-3.0230099645	1.8265328627	3.0740267419	-2.2366909745	0.1882122713
C29	1.6709947341	-6.2947965926	-3.1052382804	1.5483104149	-6.2803784765	-3.1777040710
O30	-1.0893240325	-6.5433128652	-2.5150230743	-1.1844842272	-6.6385349939	-2.5097732452
C31	-3.0872225602	-8.5634933210	-0.1470018926	-3.0599996888	-8.6546760457	-0.0188252749
H32	-2.5825889173	-6.0605134730	1.1041263995	-2.5984266157	-6.1006824975	1.1373834613
H33	-0.7980920139	-7.5480367745	-0.0300064246	-0.7938492113	-7.5625509761	-0.0079094928
H34	-2.6159302811	-4.7572872427	-1.6382036171	-2.7393193072	-4.8795693135	-1.6396787560
H35	0.5851538189	-4.2724416043	2.1879893457	0.5339655786	-4.1813077669	2.0847970776
H36	-2.1636366976	-2.5909714061	-0.5397139877	-2.3441326803	-2.6685440719	-0.6040460326
H37	-4.2527221953	-4.2182915883	0.8749465552	-4.3435221571	-4.3223099793	0.8902485306
H38	-4.3724841369	-3.3196902391	-0.6362375211	-4.5203989133	-3.4721722478	-0.6431886460
H39	-6.1617891974	-5.0684970357	-0.4498876551	-6.2485639607	-5.2707255160	-0.3741395025
H40	-5.1155561174	-5.3372083733	-1.8427144457	-5.2177833789	-5.5489451890	-1.7768337325
H41	-4.9173754280	-6.7755186225	0.8540173290	-4.9234886966	-6.8923379936	0.9597273820
H42	-5.4780911767	-7.4751781137	-0.6637264752	-5.4909449257	-7.6597527637	-0.5224172491
H43	-3.2477306447	-7.0684537651	-1.6798632427	-3.2958740521	-7.2182453268	-1.5972615132
H44	0.3739898899	-8.6482948977	0.9661525191	0.4610462982	-8.5890197890	0.9650661950
H45	2.1576505901	-8.2390986229	3.0221856143	2.2755935219	-8.0632269643	2.9651100949
H46	-0.8827196789	-9.5721112704	2.8895395177	-0.7016990087	-9.5362773250	2.9361506347
H47	-1.5555040204	-7.9799852299	2.4620780220	-1.4581124301	-7.9860000524	2.4964195709
H48	-0.4748013906	-8.1407630976	3.8630831629	-0.3382736578	-8.0690782911	3.8735492884
H49	-0.6442992424	-1.2988889017	0.4454216676	-0.2635651363	-1.8302419541	1.9033079380
H50	0.9328436402	-2.0909695370	0.6423209983	0.7549785621	-2.0105989424	0.4745766317
H51	-0.1272494960	-1.8839082278	2.0379900553	-0.8637210510	-1.2962480022	0.3233044251
H52	-0.1768481369	-5.0132598563	-1.5705130182	-0.3083740488	-5.0490352993	-1.6309660949
H53	3.6410937737	-7.6732990747	0.3751138889	3.6426665231	-7.5542297650	0.2747570793
H54	1.8098138863	-9.4970514399	-0.3208643562	1.8574368912	-9.4413634948	-0.3557835833
H55	2.8223018496	-9.1541161484	-1.7407117200	2.8267289058	-9.0941231739	-1.8038660621
H56	2.2379612346	-4.2354961493	-0.9553959852	2.0274022161	-4.1699393605	-0.9971574309
H57	3.8600143010	-5.4543877068	1.3574592095	3.8935693416	-5.2971355630	1.1669673518
H58	3.6999449255	-1.5519147495	0.3881097178	4.6692861456	-1.9935124327	1.8031692483
H59	0.9450221427	-6.5695529705	-3.8713520601	0.8093959005	-6.5806095900	-3.9216833035
H60	1.8369046741	-5.2139231523	-3.1586490566	1.6835619261	-5.1957791723	-3.2419739161
H61	2.6202827493	-6.7993902793	-3.3057308483	2.5052484691	-6.7605730608	-3.4005449127
H62	-1.0041467980	-7.5070570120	-2.5636718411	-1.0553041799	-7.5981916997	-2.5404862983
H63	-3.8694479018	-9.2200699918	-0.5410020500	-2.0922631769	-9.0301228660	-0.3661553879
H64	-3.1074800587	-8.6507291457	0.9459716599	-3.8227560831	-9.3486756922	-0.3860643749
H65	-2.1295972230	-8.9595169145	-0.4995382163	-3.0706498840	-8.7078721773	1.0764779963

Table S10. Coordinates of the significant conformers of the orignal proposed structure of wheldone (*cont.*).

	Conformer 5			Conformer 6		
atom	x	y	z	x	y	z
C1	-0.2487241811	-6.1550671956	1.0042010275	-0.0965519241	-5.7984633853	1.0138767395
C2	-0.3249284088	-4.8890643076	1.4675962177	-0.1037901529	-4.4661117235	1.2257463242
C3	-0.9810535179	-3.7313425611	0.8480953539	-0.8146596745	-3.4130110813	0.4936727675
C4	-2.0295854052	-3.7841372155	0.0032505940	-1.9004259095	-3.5741624318	-0.2873936648
C5	-2.8593266568	-4.9251549094	-0.5212437851	-2.7649113227	-4.7619621402	-0.6093232596
C6	-2.4675786937	-6.3492975778	-0.0903076769	-2.4186399674	-6.1047964184	0.0582975465
C7	-0.9403048721	-6.6210906432	-0.2577757190	-0.9112740591	-6.4780138407	-0.0657762068
C8	-4.3338985225	-4.6433574338	-0.1649948588	-4.2265402264	-4.3693080979	-0.3069933942
C9	-5.2687995755	-5.6781425870	-0.7839375702	-5.2089939705	-5.4484431748	-0.7480825563
C10	-4.8539456942	-7.0908481348	-0.3844884689	-4.8439125881	-6.7910930447	-0.1253720323
C11	-3.3963441704	-7.3911581125	-0.7573488718	-3.3993362768	-7.1991212650	-0.4432838365
C12	0.3728342948	-7.2101220915	1.8528603694	0.8014770136	-6.5896956313	1.9076500971
C13	1.0119955167	-6.8904621568	3.2157394736	0.6220048108	-8.1041837065	2.0360404919
O14	1.1782872164	-8.0829159981	3.9400073060	1.8007631132	-8.6824278916	2.5387608134
C15	2.3683633233	-6.2001703769	3.0507505223	-0.5731318646	-8.3562187858	2.9603064546
O16	0.3721532889	-8.3832851187	1.5025573469	1.6544216173	-6.0672732276	2.6122263556
C17	-0.3963187546	-2.3997382045	1.2662300852	-0.2044233223	-2.0390465278	0.6669125358
C18	-0.3743190128	-6.0445048433	-1.5769141822	-0.3271701587	-6.2156633089	-1.4775317916
C19	1.0692860182	-6.4812512318	-1.9165428476	1.0842061333	-6.7987101812	-1.7163492973
C20	2.1022175359	-6.2299709957	-0.8367965236	2.1763309616	-6.4072645439	-0.7330556268
C21	2.6342652595	-7.3948459239	-0.4524318426	2.7850294725	-7.5193374727	-0.3001832868
C22	1.9939993723	-8.5297942391	-1.1926554918	2.1428336644	-8.7275587490	-0.9110146277
O23	1.0536657309	-7.9079140420	-2.0663695346	0.9678981776	-8.2212508278	-1.5409465214
C24	2.4300661659	-4.8731459806	-0.4077461164	2.5398828257	-5.0177368057	-0.4744020897
C25	3.6028083121	-4.4810472575	0.1074212164	3.3684980862	-4.6088045244	0.4955519389
C26	3.7898111529	-3.0726368782	0.5127523645	3.6557264723	-3.1691916968	0.6457897816
O27	5.0371538688	-2.8444600605	0.9655809033	4.5551107816	-2.9354834966	1.6206092695
O28	2.9511850003	-2.1995331927	0.4586458162	3.1651839441	-2.2735558473	-0.0087815545
C29	1.5190231560	-5.8469690114	-3.2345598199	1.5503340908	-6.4871899801	-3.1434062014
O30	-1.2006838481	-6.4474603012	-2.6584615218	-1.1894272773	-6.7535557568	-2.4679480132
C31	-3.0473126295	-8.8282421314	-0.3622710638	-3.1336727202	-8.5892618550	0.1397883694
H32	-2.6691783349	-6.4300809872	0.9897230361	-2.6087405670	-5.9930920175	1.1386823502
H33	-0.7808514812	-7.7007558439	-0.3108259584	-0.8354587686	-7.5554350041	0.0756212516
H34	-2.7881040064	-4.8847010215	-1.6211626072	-2.6983585577	-4.9153323727	-1.7001647492
H35	0.1784531647	-4.6499363972	2.4026117149	0.5593122686	-4.1126520966	2.0153784982
H36	-2.3896031819	-2.8158642762	-0.3507376013	-2.2671166196	-2.6627164027	-0.7637717352
H37	-4.4374749336	-4.6540417966	0.9291753257	-4.3236723745	-4.1958482872	0.7740878815
H38	-4.6053795073	-3.6353692543	-0.4999145434	-4.4576986825	-3.4191238262	-0.8021023777
H39	-6.3025577385	-5.4799907251	-0.4803483299	-6.2309844061	-5.1625947320	-0.4761737042
H40	-5.2335995034	-5.5867445064	-1.8780453165	-5.1820973674	-5.5376212276	-1.8425491981
H41	-4.9776117881	-7.2175870503	0.7021620867	-4.9679930866	-6.7363478336	0.9669385808
H42	-5.5097364380	-7.8273975519	-0.8634288106	-5.5269266048	-7.5718693019	-0.4805113561
H43	-3.3022046648	-7.2853377514	-1.8453732902	-3.3050762022	-7.2535370075	-1.5347639226
H44	0.3327000456	-6.2454037133	3.7846866972	0.4328742284	-8.5570174847	1.0594791097
H45	1.0397346720	-8.8131138142	3.3157401209	2.2436002909	-7.9896457276	3.0554823519
H46	2.8029879732	-6.0487482843	4.0412401091	-0.7422695641	-9.4309375164	3.0572569543
H47	2.2934784799	-5.2388937880	2.5377396846	-1.4815291371	-7.8867315006	2.5704514182
H48	3.0408834598	-6.8481924157	2.4812637348	-0.3637114204	-7.9399938278	3.9503409712
H49	0.6660550281	-2.3311341570	1.0065893462	-0.7716545081	-1.2809670466	0.1225582740
H50	-0.9194876198	-1.5697413091	0.7866748971	-0.1866648969	-1.7586882751	1.7262568828
H51	-0.4754654562	-2.2698031355	2.3520552935	0.8306886652	-2.0210277853	0.3086149187
H52	-0.4087088413	-4.9503409527	-1.5680035516	-0.2879305931	-5.1370242779	-1.6670407505
H53	3.4031556682	-7.5382585636	0.2985586240	3.6526961225	-7.5739660431	0.3472380629
H54	1.4715790830	-9.2150141949	-0.5138914709	1.8616367505	-9.4886955349	-0.1747202749
H55	2.7207362078	-9.1005614854	-1.7859224245	2.8042103493	-9.1988911034	-1.6542052197
H56	1.6603263999	-4.1101025737	-0.5344509416	2.0936968467	-4.2510978682	-1.1094125959
H57	4.4435071546	-5.1546892439	0.2356268436	3.8274536584	-5.2873009617	1.2060914059
H58	5.0920122757	-1.9069883803	1.2064061069	4.6938147318	-1.9769325274	1.6598178326
H59	0.8214777880	-6.0976571847	-4.0348905678	0.8091022451	-6.8232727512	-3.8694631024
H60	1.5647919692	-4.7565187440	-3.1414829545	1.6995906089	-5.4104476243	-3.2742566854
H61	2.5148803479	-6.2181703250	-3.4909360642	2.5016778700	-6.9919517832	-3.3334653120
H62	-1.0280050905	-7.3903588890	-2.8022798209	-1.0890727752	-7.7166187808	-2.4401075045
H63	-2.1010732686	-9.1714661293	-0.7896455812	-2.1683205000	-9.0109026533	-0.1574179430
H64	-3.8280534796	-9.5148754302	-0.7047543513	-3.9056487125	-9.2878161007	-0.1984974925
H65	-2.9706704065	-8.9247920284	0.7279962744	-3.1709971639	-8.5681145151	1.2355992546

Table S10. Coordinates of the significant conformers of the orignal proposed structure of wheldone (*cont.*).

	Conformer 10			Conformer 32		
atom	x	y	z	x	y	z
C1	-0.0697136780	-5.9107620191	1.0807023659	-0.0863691406	-6.1263278718	1.0513713439
C2	-0.0416837456	-4.5972029567	1.3816482492	-0.1109109224	-4.8818629603	1.5769898487
C3	-0.6909357511	-3.4767187365	0.6943432742	-0.7135775769	-3.6691518298	1.0108251274
C4	-1.7593529698	-3.5470056140	-0.1225314573	-1.7591027392	-3.6238906095	0.1632597763
C5	-2.6583656675	-4.6801205948	-0.5344382639	-2.6270053944	-4.6951419424	-0.4335337818
C6	-2.3722553582	-6.0749510681	0.0497840670	-2.2795852987	-6.1641396099	-0.1327874926
C7	-0.8756452367	-6.4926088087	-0.0604750933	-0.7627071113	-6.4890077245	-0.2535513507
C8	-4.1107860335	-4.2562916230	-0.2284040156	-4.0929352733	-4.4137607652	-0.0410112502
C9	-5.1237422171	-5.2702940144	-0.7486373993	-5.0558625745	-5.3526860637	-0.7642116560
C10	-4.8134866739	-6.6633527805	-0.2126713915	-4.6709824962	-6.8134920790	-0.5444742162
C11	-3.3795498611	-7.0972825317	-0.5430257149	-3.2158333441	-7.0923086890	-0.9440299648
C12	0.7726676858	-6.7854344697	1.9495956767	0.4711949428	-7.2529035291	1.8564281604
C13	0.5244434685	-8.2938027213	1.9977348373	1.2572497347	-7.0625505835	3.1736138301
O14	1.6653956106	-8.9477107807	2.4944664145	1.9911334988	-5.8671408066	3.3302890588
C15	-0.7013758444	-8.5357556214	2.8849026586	0.2694118406	-7.1374408451	4.3361099340
O16	1.6307910247	-6.3374333708	2.6975457861	0.2516870802	-8.4139848870	1.5369029986
C17	-0.0326338990	-2.1416906216	0.9664147022	-0.0665785399	-2.3877013433	1.4913937070
C18	-0.2477313408	-6.1725802742	-1.4412657302	-0.0719577323	-5.8534807176	-1.4877713815
C19	1.1562508690	-6.7750776196	-1.6724163012	1.2233283933	-6.5913919518	-1.9300918971
C20	2.2409937466	-6.3839102401	-0.6797676782	2.1922805415	-6.9798260010	-0.8278201316
C21	2.8384616893	-7.4965010058	-0.2339764207	2.2241065306	-8.3137450606	-0.7089640223
C22	2.2014598288	-8.7045038852	-0.8523542209	1.2716574430	-8.9493087816	-1.6633809095
O23	1.0317887294	-8.1952198459	-1.4923804452	0.7868267047	-7.8667997153	-2.4511041445
C24	2.6386604013	-4.9978417961	-0.4488910792	2.9779214462	-6.1049553898	0.0188433057
C25	3.3003398414	-4.5675136469	0.6349941460	2.9494138101	-4.7650970641	0.0863165706
C26	3.7280090673	-3.1661321523	0.8049442901	3.7478606345	-4.1032989832	1.1323347999
O27	3.4135990346	-2.3658279679	-0.2423813968	3.7701251966	-2.7669472101	0.9971422080
O28	4.3099486108	-2.7434714062	1.7773218162	4.3186472708	-4.6713209387	2.0449053353
C29	1.6347479667	-6.4741168554	-3.0978952004	1.9122110358	-5.8386887304	-3.0665366097
O30	-1.1010208338	-6.6333189001	-2.4775771077	-0.9332484338	-5.8498417090	-2.6149300736
C31	-3.1655965503	-8.5344844861	-0.0623065315	-2.8951096970	-8.5775090938	-0.7576887532
H32	-2.5856151127	-6.0271800936	1.1304892773	-2.5280962101	-6.3522661672	0.9239822514
H33	-0.8397013396	-7.5781976877	0.0197933662	-0.6681058473	-7.5728524888	-0.3536742652
H34	-2.5772325468	-4.7653894773	-1.6318877252	-2.5589065095	-4.5647451760	-1.5245599307
H35	0.6045279603	-4.3184275046	2.2140390537	0.4169713280	-4.7216274533	2.5126252263
H36	-2.0793903896	-2.5956617315	-0.5522740339	-2.0762824575	-2.6229074807	-0.1376851128
H37	-4.2178623586	-4.1472939023	0.8600618096	-4.1946184673	-4.5368707434	1.0464519390
H38	-4.3021868748	-3.2695889695	-0.6657052637	-4.3414234252	-3.3700572325	-0.2671423302
H39	-6.1383379420	-4.9666273838	-0.4686956734	-6.0828877352	-5.1719601839	-0.4280157146
H40	-5.0867974816	-5.2890298042	-1.8462464369	-5.0294361882	-5.1327890182	-1.8401980465
H41	-4.9502017968	-6.6779544381	0.8793550437	-4.8035417705	-7.0762196314	0.5163135425
H42	-5.5179972366	-7.3938312748	-0.6276663675	-5.3386916702	-7.4686841428	-1.1165261971
H43	-3.2727716688	-7.0745804033	-1.6343849733	-3.1123998985	-6.8377979968	-2.0075374755
H44	0.3363826032	-8.6882507046	0.9957537357	1.9221818133	-7.9353490406	3.2141767398
H45	2.1308853475	-8.3006205169	3.0492419391	2.8321222665	-5.8777464253	2.8510909681
H46	-0.9196681788	-9.6052101360	2.9238285384	-0.3208101244	-8.0542125952	4.2784737267
H47	-1.5802924729	-8.0061930898	2.5033489068	-0.4047755757	-6.2760729343	4.3109523825
H48	-0.4939524217	-8.1797266600	3.8985238851	0.8215759067	-7.1172997759	5.2783022716
H49	-0.5584832559	-1.3269628987	0.4640510535	0.9943661880	-2.3549805890	1.2161831425
H50	1.0061510983	-2.1462158664	0.6175466512	-0.5566643882	-1.5092875063	1.0656246952
H51	-0.0145837357	-1.9308932127	2.0411632906	-0.1164292934	-2.3149550759	2.5836097070
H52	-0.1753760166	-5.0868976121	-1.5738060618	0.1590205002	-4.8016410692	-1.2884481996
H53	3.7064060123	-7.5445556174	0.4140063610	2.8119863908	-8.8718107644	0.0120692365
H54	1.9160278730	-9.4686138953	-0.1218812022	0.4482960275	-9.4420513713	-1.1248387979
H55	2.8704824242	-9.1699275048	-1.5924868060	1.7490161151	-9.6852940110	-2.3215261685
H56	2.3726749917	-4.2723339440	-1.2172756706	3.6467730720	-6.6159637513	0.7125378651
H57	3.5348166153	-5.2196222249	1.4701012575	2.3343511300	-4.1384994736	-0.5508693216
H58	3.7527865801	-1.4833612784	-0.0304904523	4.2892576673	-2.4078109789	1.7335023476
H59	0.9158606438	-6.8467335926	-3.8281370560	1.2123947397	-5.6970621906	-3.8917918370
H60	1.7504815189	-5.3963097313	-3.2515104296	2.2595156601	-4.8566050934	-2.7348466648
H61	2.6032481449	-6.9537915595	-3.2645956613	2.7721618587	-6.4149435920	-3.4165284338
H62	-1.0509598863	-7.6005072858	-2.4819438935	-0.9476669074	-6.7585243420	-2.9517544390
H63	-3.9590756244	-9.1791467937	-0.4533684705	-1.9735445091	-8.8739503963	-1.2680357916
H64	-3.2068574004	-8.5934152406	1.0320015623	-3.7039401889	-9.1939909411	-1.1628189681
H65	-2.2135663917	-8.9662132936	-0.3870007757	-2.7798046606	-8.8248354746	0.3046784079

Table S10. Coordinates of the significant conformers of the orignal proposed structure of wheldone (*cont.*).

	Conformer 35			Conformer 9		
atom	x	y	z	x	y	z
C1	-0.7905899902	-6.9624111044	1.6923995006	-0.1982922690	-6.1078886314	1.0143207239
C2	-1.3748961133	-6.2158448722	2.6502160223	-0.2568739980	-4.8393951445	1.4715733319
C3	-1.9756147007	-4.8798452958	2.5361617245	-0.8838068105	-3.6741039235	0.8358252374
C4	-2.4522834334	-4.3050829677	1.4158144474	-1.9266892222	-3.7100464706	-0.0163152761
C5	-2.6221176485	-4.8120613452	0.0102027023	-2.7703492927	-4.8385942849	-0.5452448225
C6	-2.1752240752	-6.2547600965	-0.2781251341	-2.4024045763	-6.2682659199	-0.1114775603
C7	-0.7405805487	-6.5720302070	0.2332237905	-0.8771258872	-6.5614938524	-0.2585359563
C8	-4.1008047537	-4.6167989770	-0.3847161309	-4.2442343645	-4.5376162413	-0.2025178668
C9	-4.3454487256	-4.9776016613	-1.8462230924	-5.1859263503	-5.5593262768	-0.8337375061
C10	-3.8753948244	-6.3994249319	-2.1344966984	-4.7937979904	-6.9785782073	-0.4341141130
C11	-2.4007969994	-6.6102740792	-1.7679586985	-3.3360991867	-7.2975868263	-0.7904998732
C12	-0.2219379899	-8.2956236996	2.0406424072	0.3752344101	-7.1746300525	1.8832358103
C13	-0.0535441785	-8.7424565610	3.4995677740	1.0083771384	-6.8610421219	3.2500791023
O14	0.2529133873	-10.1122415926	3.5338721669	1.1433978309	-8.0534140540	3.9808547781
C15	1.0569512018	-7.9487824593	4.1942171960	2.3798926032	-6.1971915424	3.1014949789
O16	0.1370024936	-9.0733198316	1.1688382479	0.3307145298	-8.3505784413	1.5468668852
C17	-2.0303007788	-4.1309490964	3.8509768620	-0.2704273844	-2.3524705251	1.2444783288
C18	0.2874992151	-5.4509563918	-0.0087359901	-0.2774060463	-5.9893353690	-1.5649090767
C19	1.7579817655	-5.8926399300	0.2556326797	1.1476402000	-6.4899089410	-1.8976967851
C20	2.6622998219	-4.6537103290	0.2258914919	2.1740903425	-6.3323711198	-0.7940909654
C21	2.8852440607	-4.2654008131	1.4904619888	2.6061073620	-7.5390162766	-0.4134865841
C22	2.2289007732	-5.2210289421	2.4396854695	1.9045100887	-8.6125782735	-1.1878499272
O23	1.8730678343	-6.3204750262	1.6197206680	1.0545294039	-7.9089894035	-2.0926023643
C24	3.2167367652	-3.9544466945	-0.9236695038	2.6037899568	-5.0125329447	-0.3358699374
C25	3.0901898238	-4.2701814015	-2.2230815976	3.8078333294	-4.7450399191	0.1883620438
C26	3.7801177552	-3.4334095536	-3.2307387073	4.2089842527	-3.4092414114	0.6665939711
O27	3.5872103554	-3.9024923960	-4.4791396262	3.2440911404	-2.4679752413	0.5384787076
O28	4.4428476442	-2.4465718234	-3.0013351484	5.2942947701	-3.1540243828	1.1347642935
C29	2.1960577015	-7.0547995017	-0.6258249178	1.6490695297	-5.8424333788	-3.1896472915
O30	0.1569742809	-5.0223556132	-1.3572811055	-1.1087130719	-6.3388931330	-2.6606085846
C31	-1.9936970313	-8.0496889938	-2.0969151599	-3.0102686047	-8.7390771243	-0.3908831077
H32	-2.8446394418	-6.9196364488	0.2911893596	-2.6193657278	-6.3478404764	0.9656891035
H33	-0.3877580086	-7.4525086010	-0.3053558683	-0.7352686460	-7.6436337485	-0.3111252817
H34	-2.0332853493	-4.1476637896	-0.6429757759	-2.6879782823	-4.7990113529	-1.6441378957
H35	-1.3753498159	-6.6018205765	3.6675958658	0.2328178554	-4.6063731148	2.4153354368
H36	-2.8405521360	-3.2919721240	1.5403046233	-2.2642821115	-2.7372762645	-0.3802498182
H37	-4.7221656468	-5.2500826283	0.2641780824	-4.3592320182	-4.5498739052	0.8905050549
H38	-4.3966797300	-3.5786636702	-0.1922259866	-4.4998105483	-3.5255610548	-0.5377683655
H39	-5.4078698999	-4.8656781773	-2.0890857979	-6.2201819693	-5.3490169958	-0.5403292570
H40	-3.7951253098	-4.2765807651	-2.4887826418	-5.1383400486	-5.4655687130	-1.9271499132
H41	-4.4900193195	-7.1118172144	-1.5625232789	-4.9315788849	-7.1070787945	0.6506365775
H42	-4.0220485471	-6.6392357053	-3.1941114118	-5.4535560659	-7.7051575190	-0.9226958573
H43	-1.7947064890	-5.9261138726	-2.3751955521	-3.2280057337	-7.1925911349	-1.8774395422
H44	-1.0040494274	-8.5974559426	4.0283842060	0.3339832655	-6.1992704929	3.8069288645
H45	0.4483915413	-10.3797757788	2.6213616850	0.9651254600	-8.7836397431	3.3669050275
H46	1.2014598485	-8.3632173098	5.1942593422	2.8141454583	-6.0782371271	4.0964268155
H47	0.8134862976	-6.8858024382	4.2763215251	2.3281559301	-5.2224393943	2.6119219361
H48	1.9870812300	-8.0528512000	3.6289626727	3.0418730388	-6.8433822799	2.5174210208
H49	-1.0299897147	-4.0254843336	4.2873678539	0.7980256963	-2.3263638100	1.0013283747
H50	-2.4549466422	-3.1328647077	3.7257495583	-0.7602722667	-1.5153348894	0.7427383416
H51	-2.6424920637	-4.6733516647	4.5804344231	-0.3604492812	-2.2020217625	2.3266729928
H52	0.0717177818	-4.6134958914	0.6712439384	-0.2630040092	-4.8949799458	-1.5383001668
H53	3.4553679924	-3.3953465235	1.7981574676	3.3404191296	-7.7501363682	0.3560779363
H54	1.3333982839	-4.7772160436	2.9130827166	1.3026938269	-9.2564288472	-0.5336890499
H55	2.8934991093	-5.5760634074	3.2344729726	2.6027255966	-9.2382513274	-1.7589182754
H56	3.8276767417	-3.0815131499	-0.6932585143	1.8911984951	-4.1947634291	-0.4472232586
H57	2.5187907127	-5.1181606348	-2.5830692101	4.5764150953	-5.5041527792	0.2921003934
H58	4.0669138240	-3.3178832187	-5.0855753178	3.6230445718	-1.6360455647	0.8606176441
H59	1.7940491480	-7.9826642980	-0.2114946240	0.9500448306	-6.0305280371	-4.0057533351
H60	1.8182273288	-6.9389135311	-1.6443330353	1.7516597234	-4.7592656522	-3.0620619623
H61	3.2864238600	-7.1256004265	-0.6483838299	2.6269868004	-6.2582908722	-3.4455179842
H62	0.6202715964	-4.1836907529	-1.4733886018	-0.9660368585	-7.2836269364	-2.8248404945
H63	-0.9104868631	-8.1952384697	-2.0984858474	-2.0647658082	-9.0956608730	-0.8090416118
H64	-2.3592211591	-8.3261512239	-3.0910502146	-3.7970049209	-9.4154157363	-0.7399593430
H65	-2.4248640698	-8.7536748856	-1.3740022547	-2.9451474428	-8.8352549062	0.7001128587

Table S11. Total Gibbs Free Energy and Boltzmann distribution of the significant conformers (> 1%) conformers of the orignal proposed structure of wheldone.

Structure:	Conformer 1 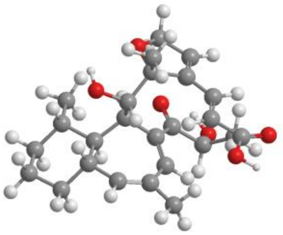	Conformer 3 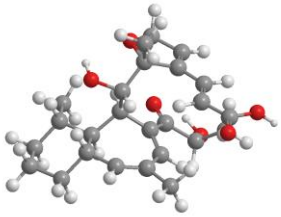	Conformer 2 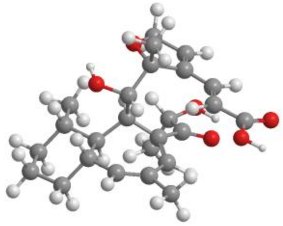
Total Gibbs Free Energy (Hartrees):	-1423.425429	-1423.425419	-1423.424843
Population:	30.24%	23.96%	12.59%
Structure:	Conformer 4 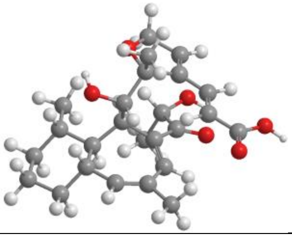	Conformer 14 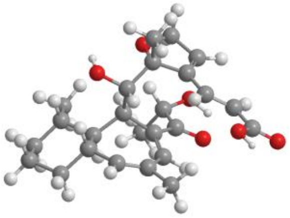	Conformer 7 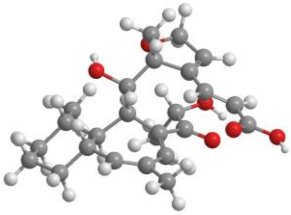
Total Gibbs Free Energy (Hartrees):	-1423.424271	-1423.424098	-1423.424072
Population:	6.36%	5.42%	5.28%
Structure:	Conformer 5 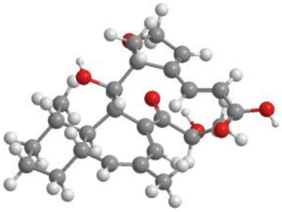	Conformer 6 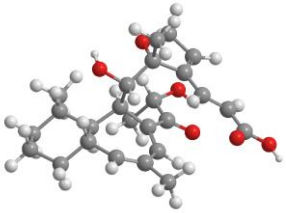	Conformer 10 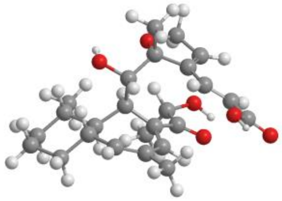
Total Gibbs Free Energy (Hartrees):	-1423.424343	-1423.422973	-1423.423119
Population:	2.81%	2.78%	2.51%
Structure:	Conformer 32 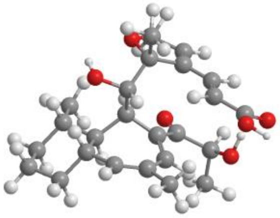	Conformer 35 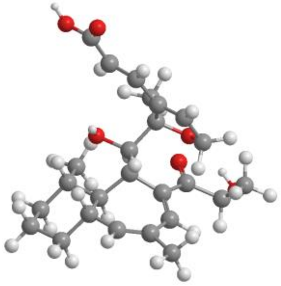	Conformer 9 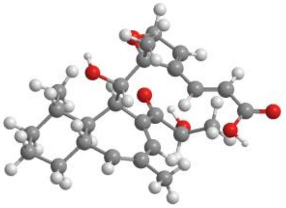
Total Gibbs Free Energy (Hartrees):	-1423.423244	-1423.423322	-1423.422627
Population:	1.81%	1.59%	1.05%

Total Gibbs free energy, G_{tot} (H_{tot} - T*S)

Table S12. Comparison of the experimental and calculated ^{1}H- and ^{13}C-NMR chemical shifts of the revised structure of wheldone, using the numbering scheme in the drawing below.

Position	Assigned ^{13}C Chemical Shift (ppm)	Boltzmann-Weighted Predicted ^{13}C Chemical Shift (ppm)	Δ ^{13}C Chemical Shift (ppm)	Assigned ^{1}H Chemical Shift (ppm)	Boltzmann-Weighted Predicted ^{1}H Chemical Shift (ppm)	Δ ^{1}H Chemical Shift (ppm)
**1**	170.3	168.0	2.3			
**2**	122.7	116.9	5.8	5.71	5.59	0.12
**3**	137.4	142.6	-5.2	7.40	7.57	-0.17
**4**	137.1	136.9	0.2			
**5**	142.8	151.5	-8.7	6.45	6.75	-0.30
**6**	95.2	97.9	-2.7			
**7**	74.8	74.7	0.1	4.76	4.81	-0.05
				4.82	4.72	0.10
**8**	76.1	78.4	-2.3	3.54	3.53	0.01
**9**	43.0	44.1	-1.1	3.85	3.97	-0.12
**10**	44.0	44.4	-0.4	1.96	1.98	-0.02
**11**	31.6	32.4	-0.8	1.22	1.11	0.11
**12**	36.4	35.4	1.0	0.95	0.93	0.02
				1.57	1.44	0.13
**13**	23.5	24.5	-1.0	1.22	1.13	0.09
				1.48	1.46	0.02
**14**	32.8	32.7	0.1	1.50	1.49	0.01
				1.79	1.78	0.01
**15**	34.6	36.7	-2.1	2.89	2.83	0.06
**16**	143.0	152.0	-9.0	5.92	6.26	-0.34
**17**	133.6	135.3	-1.7			
**18**	157.3	165.7	-8.4			
**19**	122.0	119.2	2.8	6.57	6.26	0.31
**20**	208.7	206.4	2.3			
**21**	74.2	74.5	-0.3	4.42	4.40	0.02
**22**	20.8	21.2	-0.4	1.35	1.34	0.01
**23**	19.3	18.6	0.7	1.42	1.45	-0.03
**24**	20.5	19.0	1.5	0.79	0.76	0.03
**25**	20.0	20.1	-0.1	1.91	1.92	-0.01

153 Conformers used to weight Boltzmann populations. The 153 conformers make up a total of 100%. No imaginary frequencies were present in frequency data after optimization.

^{13}C Mean Absolute Error: 2.4. Optimization: Gas phase, B3LYP/6-31+G(d,p), Chemical shift prediction: PCM MeOH, mPW1PW91/6-311+G(2d,p).[8]

^{1}H Mean Absolute Error: 0.09. Optimization: Gas phase, B3LYP/6-31+G(d,p), Chemical shift prediction: PCM MeOH, WP04/aug-cc-pVDZ.[8]

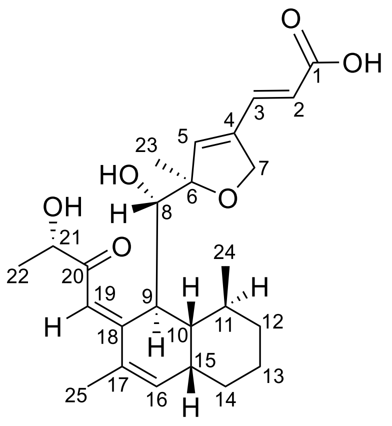

Table S13. Coordinates of the significant conformers of the revised structure of wheldone.

	Conformer 1			Conformer 3		
atom	x	y	z	x	y	z
C1	2.1661069031	-0.0574478591	1.0333766697	2.1512840479	-0.0729335227	1.0329872401
C2	2.0882451805	0.9541447808	2.1006718479	2.0809138827	0.9364126056	2.1026720454
C3	1.3502106933	2.0732046524	1.9123008922	1.3550943006	2.0635023628	1.9158207068
C4	0.5959536995	2.4247351344	0.6606654133	0.6070467871	2.4260743994	0.6638599323
C5	1.0893868641	1.5880261149	-0.5431300198	1.0954906830	1.5887105615	-0.5416716582
C6	1.1875673448	0.0928059318	-0.1278068143	1.1790044932	0.0912326324	-0.1313672125
C7	0.6027793106	3.9423951860	0.3791084715	0.6273904106	3.9445014133	0.3868526547
C8	1.9537150139	4.4324448870	-0.1509967100	1.9829616634	4.4247130340	-0.1402769609
C9	2.3529872755	3.6380046438	-1.3985112109	2.3775307493	3.6301460095	-1.3892416962
C10	2.4269169146	2.1197917874	-1.1396034910	2.4384608789	2.1107008227	-1.1342610778
C11	2.8291668890	0.7362866592	3.4012496300	2.8160793103	0.7067868993	3.4046656233
C12	3.0973710660	-1.0555815763	1.1172214440	3.0706543284	-1.0824636508	1.1182988843
C13	3.3131806190	-2.1671530748	0.1989179039	3.2782223504	-2.1939081409	0.1977367678
C14	4.6590592237	-2.8986263310	0.2870082927	4.6163101113	-2.9392263932	0.2882241779
C15	5.8417171215	-1.9894513323	-0.0809557696	5.8084904093	-2.0424241701	-0.0794785815
O16	2.4827448763	-2.5937548990	-0.6218779558	2.4461789744	-2.6101926457	-0.6269318282
C17	-0.1714463610	-0.5883062484	0.2453626027	-0.1876101689	-0.5785677855	0.2332886901
C18	-1.2870618280	-0.5499220996	-0.8550285068	-1.2978754415	-0.5225698119	-0.8712632255
O19	-1.8155636719	0.7992164101	-0.9606833604	-1.8140893311	0.8315430131	-0.9706448753
C20	-3.2312064756	0.8183959731	-0.7333540026	-3.2287251043	0.8642573349	-0.7378687734
C21	-3.5861450960	-0.5953114770	-0.3367465798	-3.5974258602	-0.5494364410	-0.3551579840
C22	-2.4771428730	-1.3533587911	-0.3963073525	-2.4965472034	-1.3182535068	-0.4222255529
C23	-4.9104431068	-1.0519163841	0.0228662074	-4.9255062454	-0.9964438029	0.0055769745
C24	-6.0198494115	-0.2821122304	0.0420449424	-6.0216143704	-0.2072013757	0.0406760953
C25	-7.3257952495	-0.8533655243	0.4133643150	-7.3659315972	-0.6770966425	0.4034149691
O26	-8.3108864197	0.0868906528	0.3656091011	-7.4294481029	-2.0048876145	0.7026140115
O27	-7.5514101709	-2.0086108443	0.7288257022	-8.3534374240	0.0371654210	0.4404683953
C28	-0.7973627197	-1.0188079737	-2.2347331131	-0.8064266190	-0.9869814563	-2.2515009474
H29	-0.5995517878	-0.1020454184	1.1286098852	-0.6140706598	-0.0930438848	1.1175678648
O30	4.6495324339	-4.0376610919	-0.5455769937	4.5959450870	-4.0791653798	-0.5429105229
H31	1.5807165958	-0.4745013497	-0.9716976972	1.5700493844	-0.4763516669	-0.9759936596
H32	3.2263326533	1.9572470518	-0.4024800606	3.2353118658	1.9395800115	-0.3963227212
H33	3.7963423505	-1.0326831446	1.9469589581	3.7674914355	-1.0688442434	1.9503397366
O34	0.0410962381	-1.9475364323	0.6112524541	0.0090582535	-1.9422473336	0.5919720645
H35	0.3312117731	1.6728134735	-1.3280820182	0.3396819509	1.6830630527	-1.3277644978
H36	-0.4558843109	2.1497448880	0.8337129130	-0.4474330537	2.1600047435	0.8340153559
C37	2.8269015420	1.3930723587	-2.4344531565	2.8346937757	1.3843099735	-2.4303442121
H38	4.7744508794	-3.2225800217	1.3350479384	4.7270699949	-3.2629994877	1.3369425857
H39	1.2807387449	2.7859724697	2.7349633585	1.2909648883	2.7743251118	2.7403958444
H40	-0.1732653632	4.1564177021	-0.3678507978	-0.1458418221	4.1673048986	-0.3604259350
H41	0.3186452009	4.4852820484	1.2901849033	0.3466494970	4.4872878696	1.2989775098
H42	2.7252404129	4.3136733021	0.6232857015	2.7524992835	4.2978596814	0.6346484341
H43	1.9026633162	5.5036432119	-0.3811960190	1.9409356904	5.4968429513	-0.3680169876
H44	1.6222547733	3.8303209174	-2.1991804203	1.6495695343	3.8306255354	-2.1904442595
H45	3.3235991284	3.9841016178	-1.7760065860	3.3515406032	3.9693029161	-1.7644302057
H46	3.9170023444	0.7537068707	3.2601176371	3.9045400105	0.7138055968	3.2665394199
H47	2.5812024188	1.5236700378	4.1176202174	2.5739150342	1.4941418150	4.1230570821
H48	2.5744473792	-0.2282218788	3.8546596590	2.5504548236	-0.2567251198	3.8541811536
H49	6.7620006770	-2.5784098845	-0.0438130467	6.7227895421	-2.6404798756	-0.0399712807
H50	5.9379093763	-1.1462666024	0.6098619634	5.9117720908	-1.1989440428	0.6100997808
H51	5.7229667395	-1.6036615160	-1.0982935365	5.6949075173	-1.6571213563	-1.0976634902
H52	-3.4598145153	1.5488531386	0.0554751409	-3.4470606122	1.5881929591	0.0597674000
H53	-3.7512576321	1.1378891031	-1.6488311876	-3.7477084928	1.1997360440	-1.6480137745
H54	-2.4055463950	-2.4081842633	-0.1638761105	-2.4349110778	-2.3756003071	-0.1982099076
H55	-5.0169893312	-2.1007734783	0.2930471630	-5.0311959193	-2.0479064708	0.2611063008
H56	-6.0024357282	0.7724084947	-0.2145742212	-5.9810715052	0.8492990657	-0.2031655977
H57	-9.1370494440	-0.3580099829	0.6187699474	-8.3562521019	-2.1927654465	0.9241669968
H58	-0.0517671974	-0.3295370843	-2.6390483440	-0.0544536625	-0.3008906662	-2.6493816749
H59	-0.3530950662	-2.0169863114	-2.1732365643	-0.3698029320	-1.9887436982	-2.1940393664
H60	-1.6376837078	-1.0580120832	-2.9335573763	-1.6443106214	-1.0158996310	-2.9538131270
H61	3.7827358067	-4.0441833452	-0.9911199479	3.7305271720	-4.0759008080	-0.9910802922
H62	0.7770250154	-2.3061359673	0.0805434266	0.7462151881	-2.3043564543	0.0650921537
H63	3.7452750343	1.8269022910	-2.8459331051	2.0554589690	1.4936222666	-3.1954233950
H64	3.0137448526	0.3256608719	-2.2854547125	3.7575009695	1.8115800578	-2.8389235728
H65	2.0452859023	1.4937284126	-3.1982250508	3.0123604731	0.3148813205	-2.2840604449

Table S14. Total Gibbs Free Energy and Boltzmann distribution of the significant conformers (> 1%) conformers of the revised structure of wheldone.

	Conformer 1	Conformer 3
Structure:	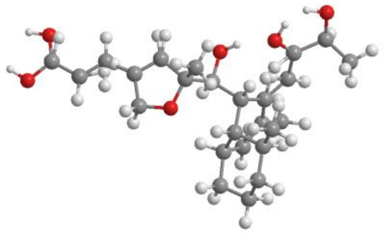	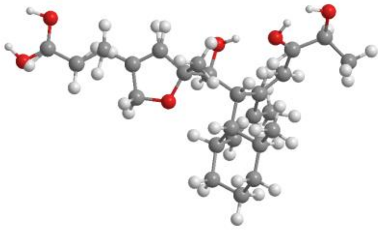
Total Gibbs Free Energy (Hartrees):	-1424.023475	-1424.022916
Population:	63.78%	35.25%

Total Gibbs free energy, G_{tot} (H_{tot} - T*S)

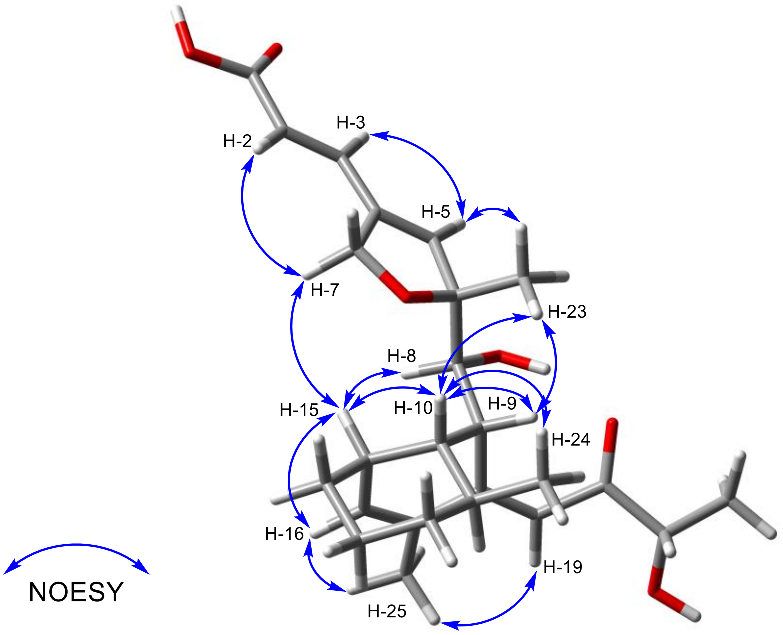

Figure S4. 3D representation of wheldone and key NOESY correlations.

Table S15. MAE and RMSD for all possible configurations tested.

6*R*,8*R*,9*R*,10*R*,11*S*,15*S*,21*S*		6*R*,8*R*,9*S*,10*R*,11*S*,15*S*,21*S*		6*R*,8*S*,9*R*,10*R*,11*S*,15*S*,21*S*		6*R*,8*S*,9*S*,10*R*,11*S*,15*S*,21*S*	
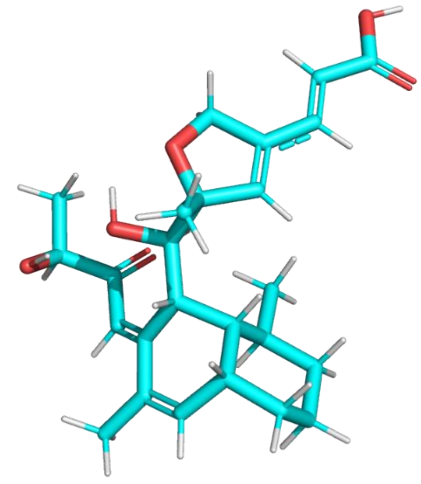		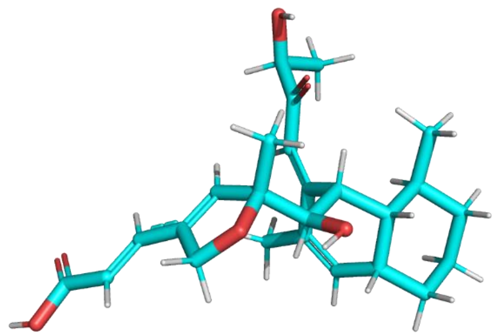		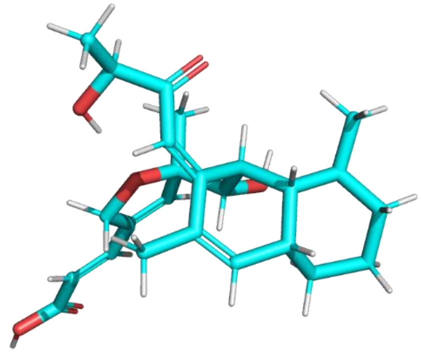		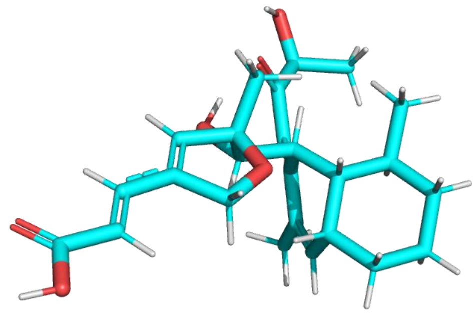	
^{13}C δ RMSD (ppm):	4.2	^{13}C δ RMSD (ppm):	4.3	^{13}C δ RMSD (ppm):	4.6	^{13}C δ RMSD (ppm):	3.7
^{13}C δ MAE (ppm):	3.2	^{13}C δ MAE (ppm):	3.0	^{13}C δ MAE (ppm):	3.2	^{13}C δ MAE (ppm):	2.4
^{13}C δ Max Deviation (ppm):	9.8	^{13}C δ Max Deviation (ppm):	11.0	^{13}C δ Max Deviation (ppm):	13.9	^{13}C δ Max Deviation (ppm):	9.0
^{1}H δ RMSD (ppm):	0.34	^{1}H δ RMSD (ppm):	0.22	^{1}H δ RMSD (ppm):	0.36	^{1}H δ RMSD (ppm):	0.13
^{1}H δ MAE (ppm):	0.25	^{1}H δ MAE (ppm):	0.16	^{1}H δ MAE (ppm):	0.27	^{1}H δ MAE (ppm):	0.09
^{1}H δ Max Deviation (ppm):	0.87	^{1}H δ Max Deviation (ppm):	0.50	^{1}H δ Max Deviation (ppm):	0.65	^{1}H δ Max Deviation (ppm):	0.35

Table S15. MAE and RMSD for all possible configurations tested (*cont*).

6*S*,8*R*,9*R*,10*R*,11*S*,15*S*,21*S*		6*S*,8*R*,9*S*,10*R*,11*S*,15*S*,21*S*		6*S*,8*S*,9*R*,10*R*,11*S*,15*S*,21*S*		6*S*,8*S*,9*S*,10*R*,11*S*,15*S*,21*S*	
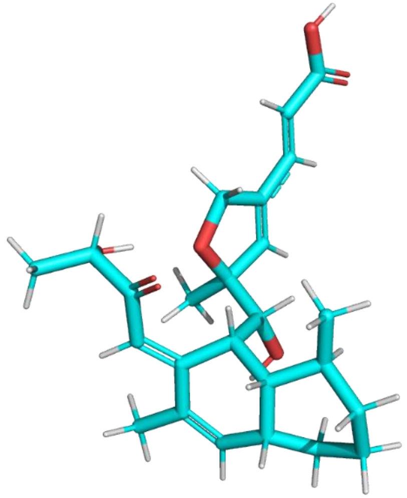		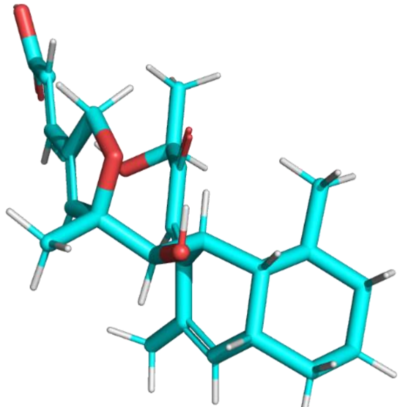		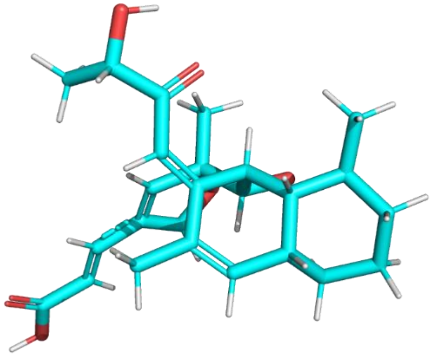		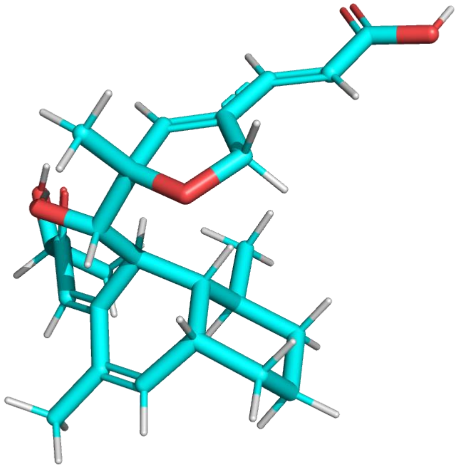	
^{13}C *δ* RMSD (ppm):	3.6	^{13}C *δ* RMSD (ppm):	4.1	^{13}C *δ* RMSD (ppm):	3.6	^{13}C *δ* RMSD (ppm):	3.5
^{13}C *δ* MAE (ppm):	2.6	^{13}C *δ* MAE (ppm):	3.1	^{13}C *δ* MAE (ppm):	2.7	^{13}C *δ* MAE (ppm):	2.6
^{13}C *δ* Max Deviation (ppm):	9.0	^{13}C *δ* Max Deviation (ppm):	10.3	^{13}C *δ* Max Deviation (ppm):	8.9	^{13}C *δ* Max Deviation (ppm):	8.6
^{1}H *δ* RMSD (ppm):	0.17	^{1}H *δ* RMSD (ppm):	0.23	^{1}H *δ* RMSD (ppm):	0.17	^{1}H *δ* RMSD (ppm):	0.17
^{1}H *δ* MAE (ppm):	0.13	^{1}H *δ* MAE (ppm):	0.15	^{1}H *δ* MAE (ppm):	0.13	^{1}H *δ* MAE (ppm):	0.13
^{1}H *δ* Max Deviation (ppm):	0.38	^{1}H *δ* Max Deviation (ppm):	0.71	^{1}H *δ* Max Deviation (ppm):	0.38	^{1}H *δ* Max Deviation (ppm):	0.35

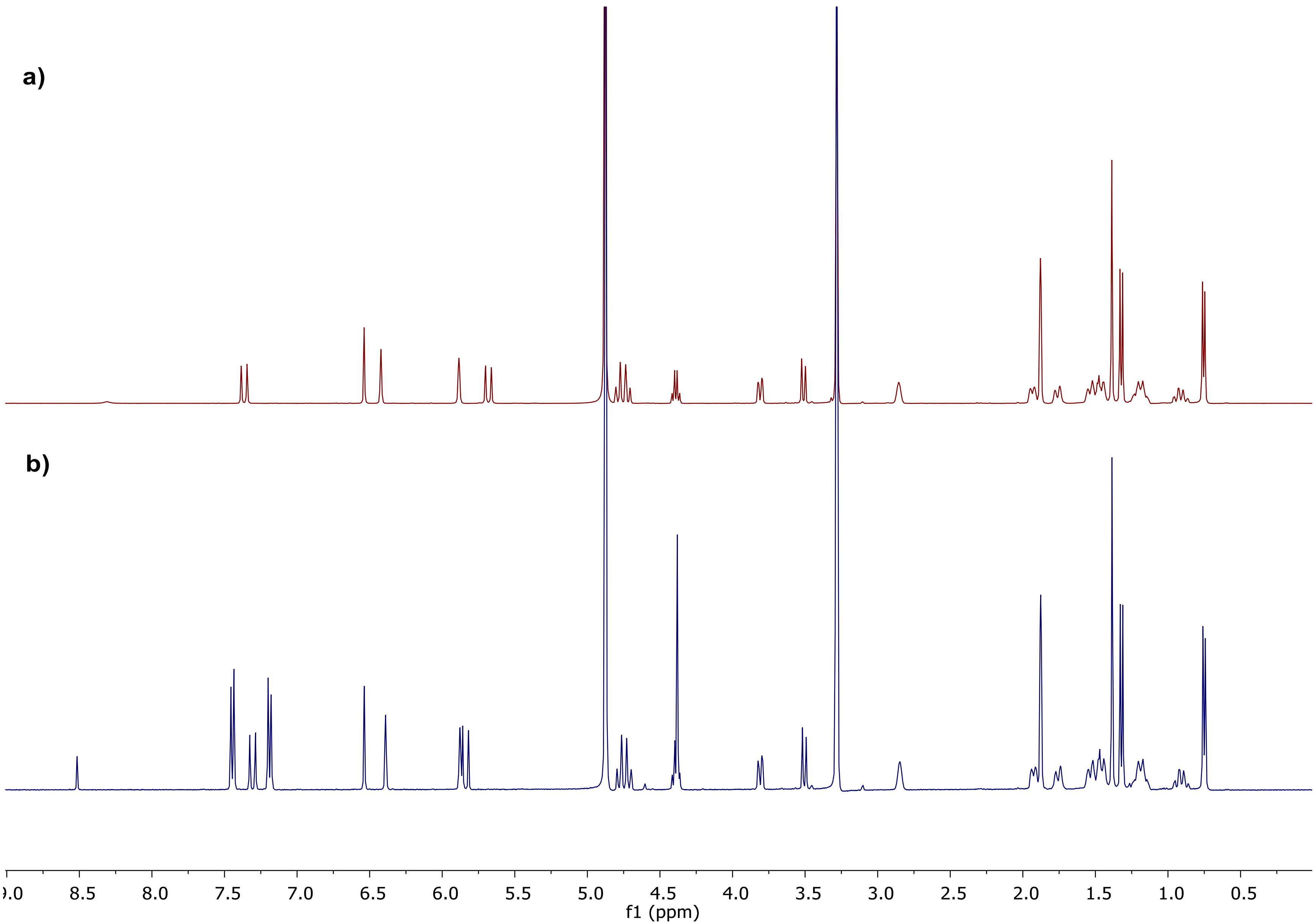

Figure S5. ^{1}H NMR spectra comparison between wheldone (**a**) and wheldone *p*-bromobenzylamide (**b**) in CD_3OD (700 MHz).

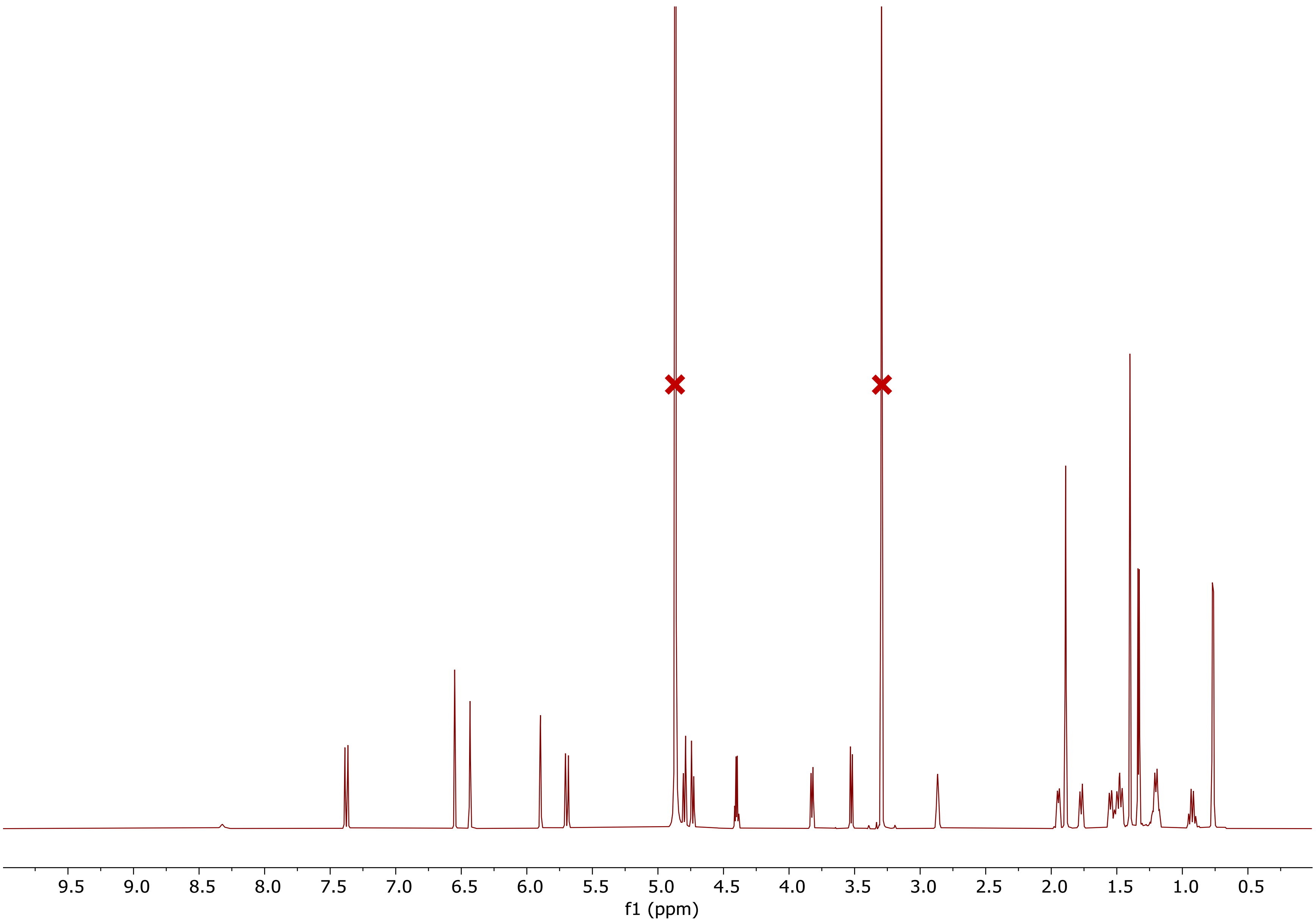

Figure S6. ^{1}H NMR spectrum of wheldone in CD_3OD (700 MHz).

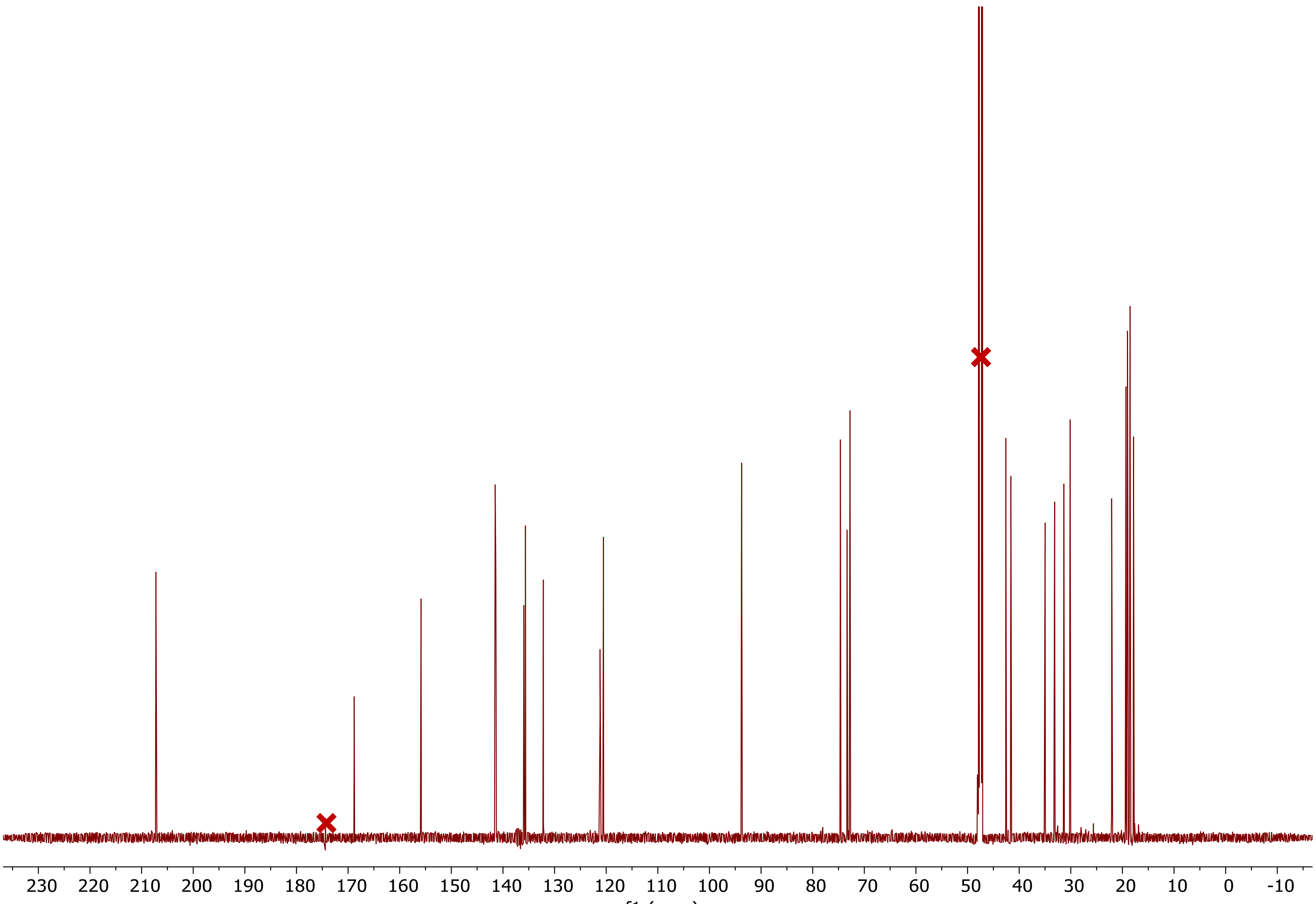

Figure S7. ^{13}C NMR spectrum of wheldone in CD_3OD (175 MHz).

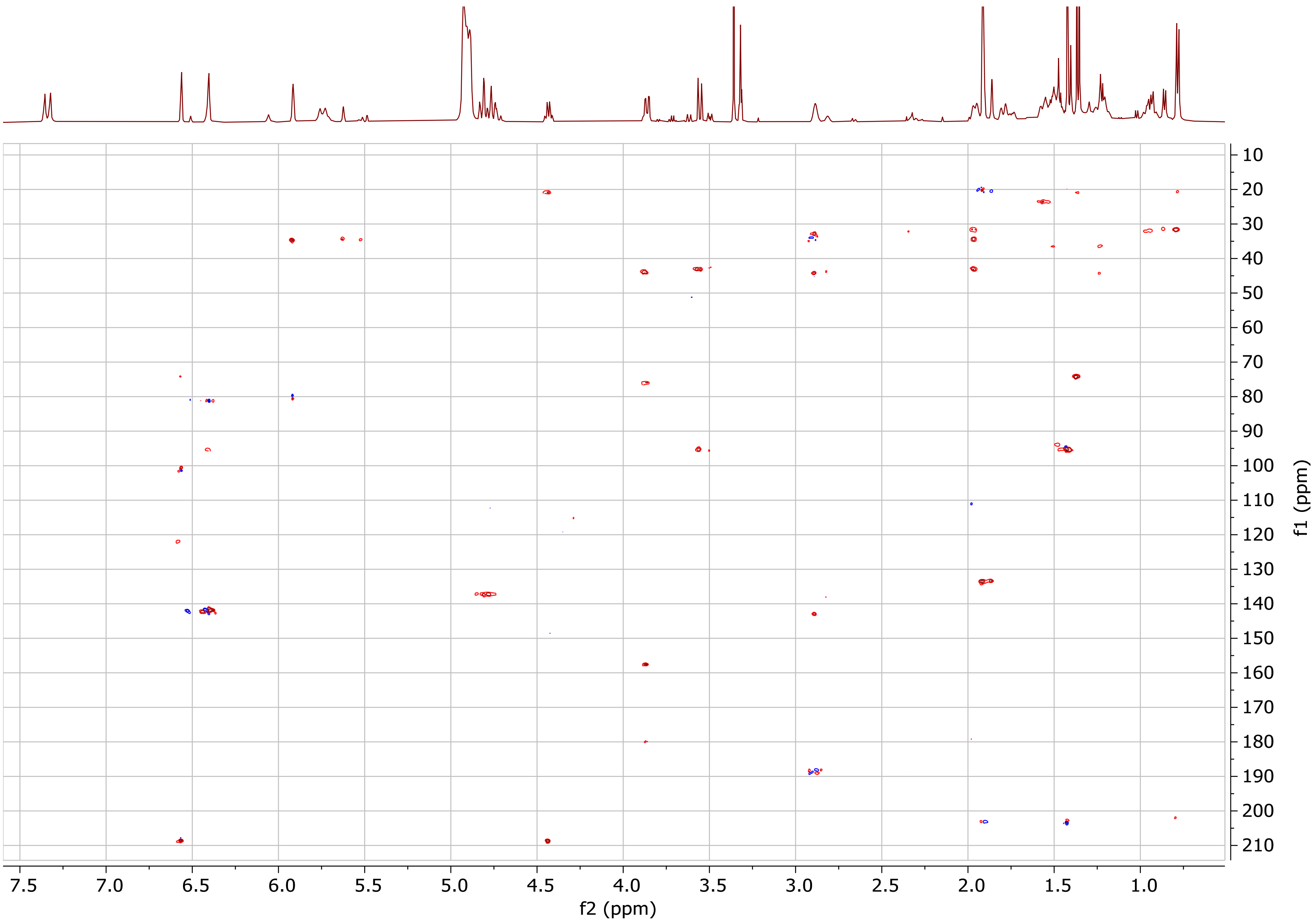

Figure S8. 1,1-HD-ADEQUATE NMR spectrum of wheldone in CD_3OD.

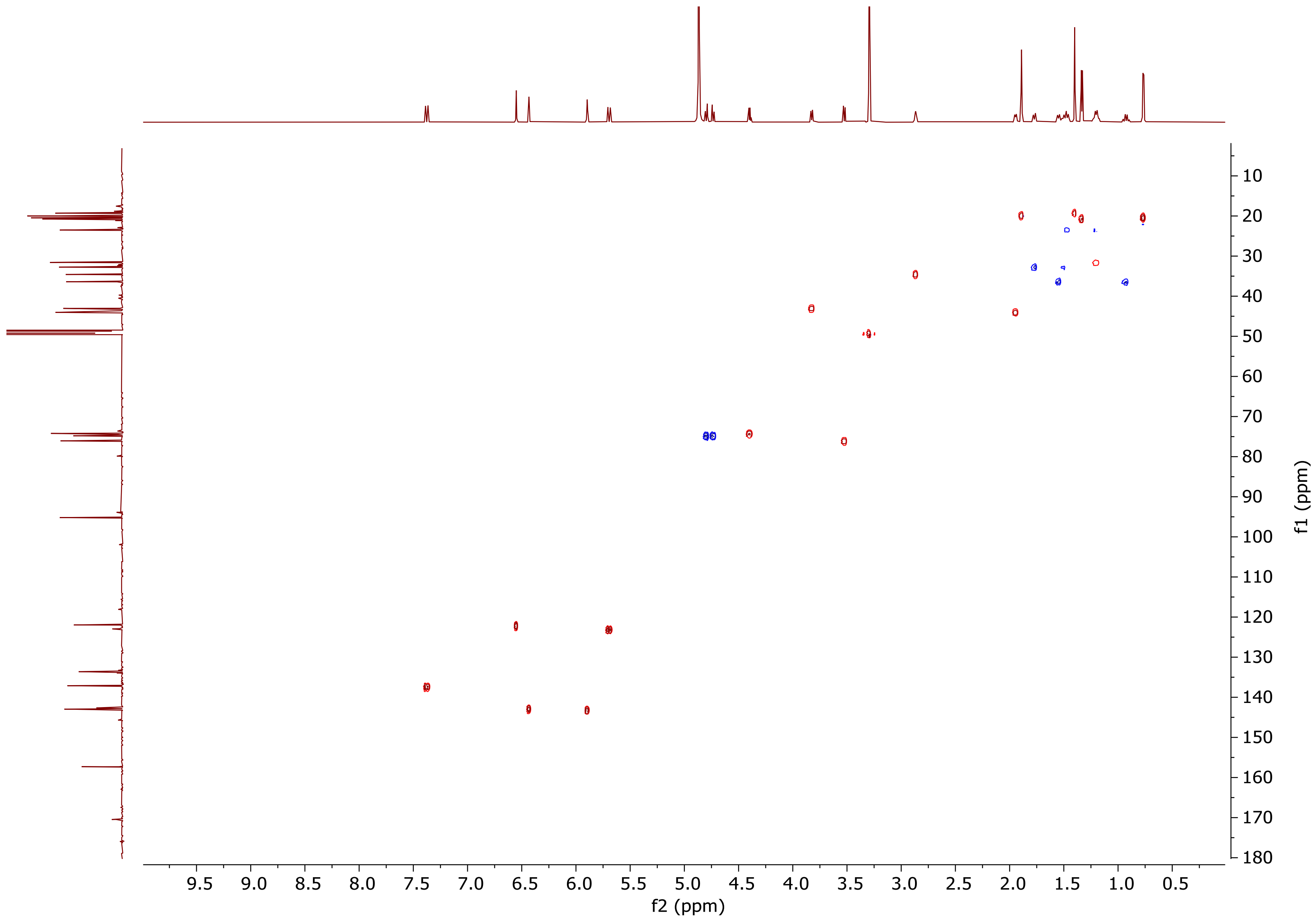

Figure S9. gHSQCAD spectrum of wheldone in CD_3OD.

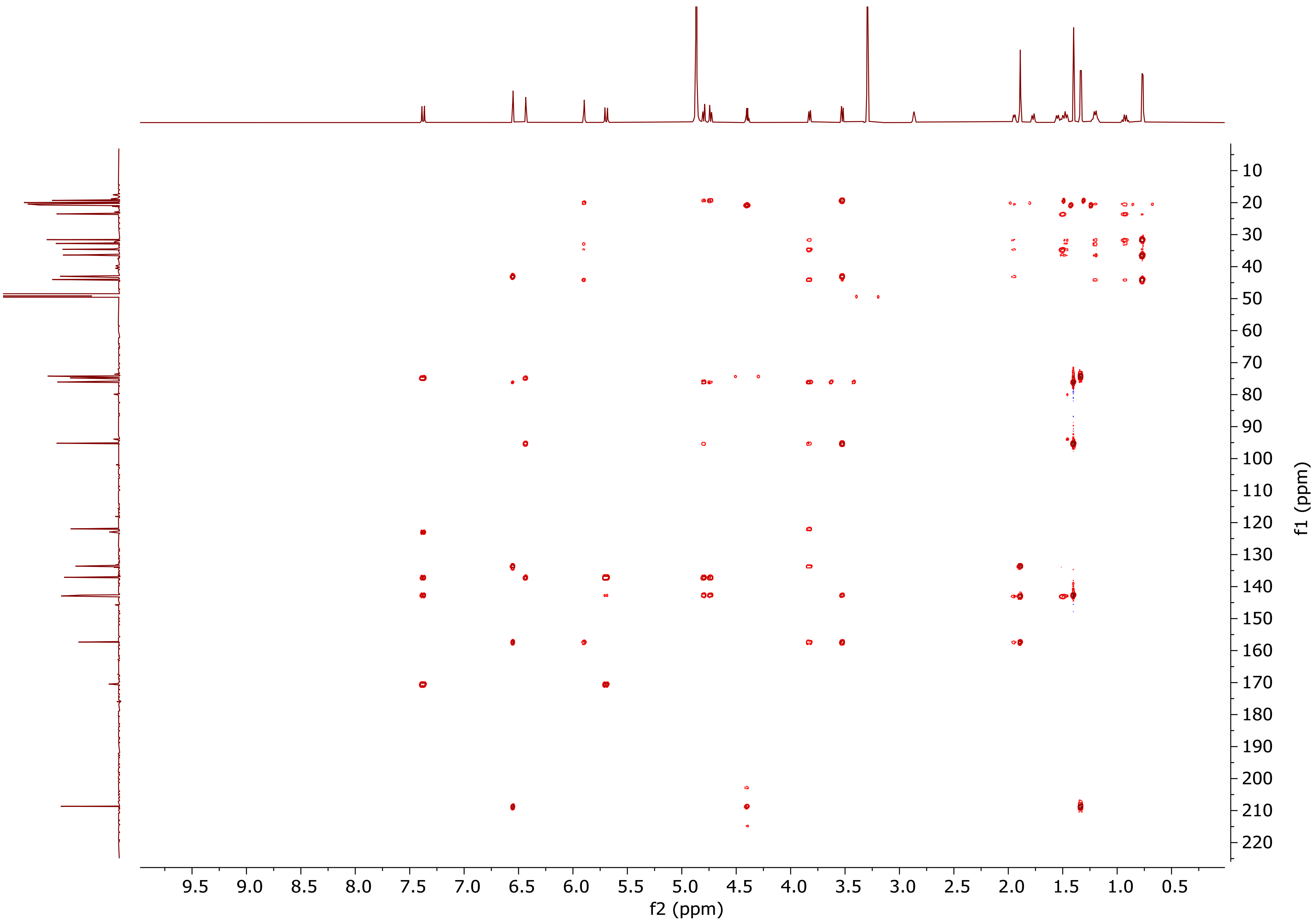

Figure S10. gHMBCAD spectrum of wheldone in CD_3OD.

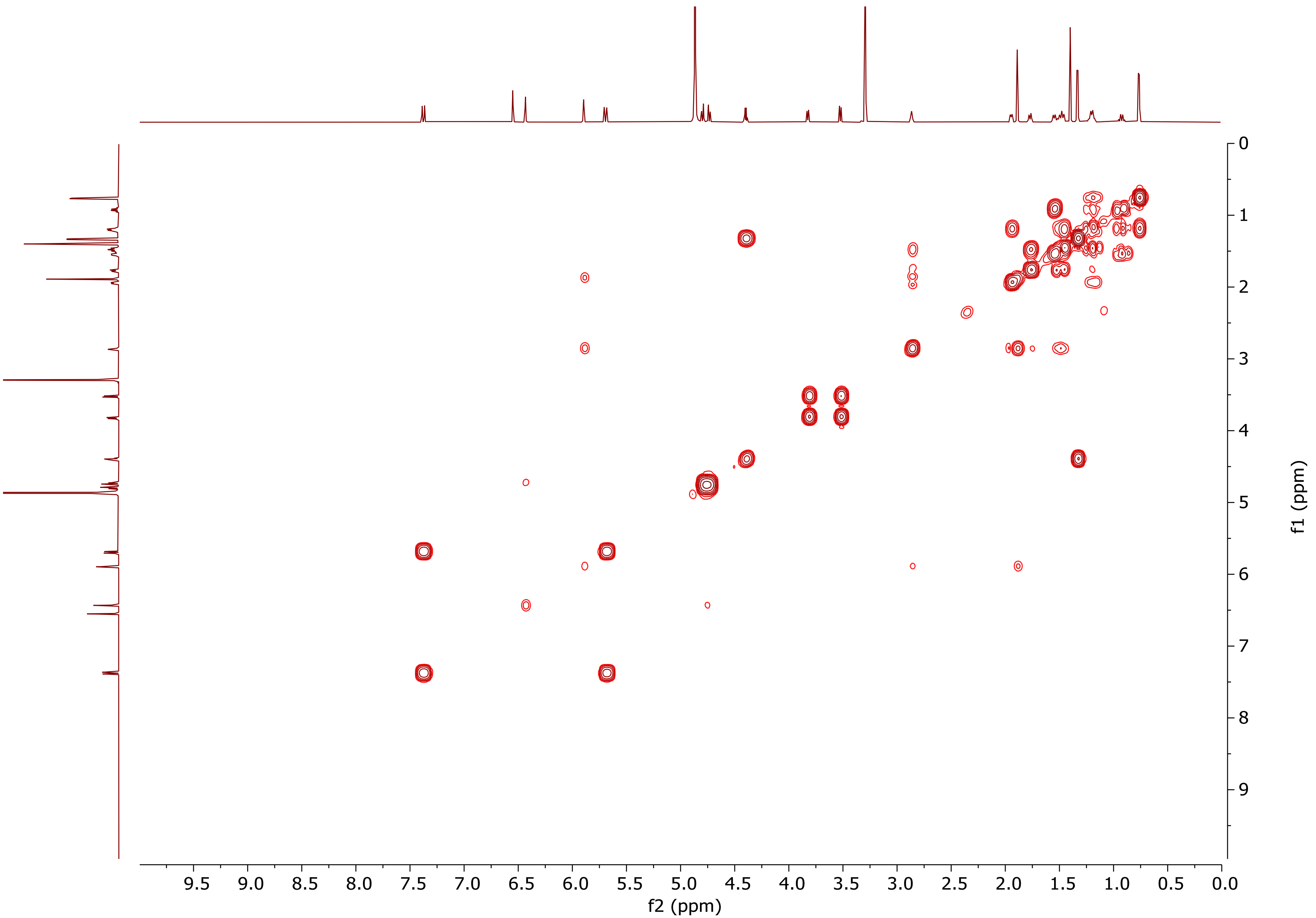

Figure S11. gCOSY spectrum of wheldone in CD_3OD.

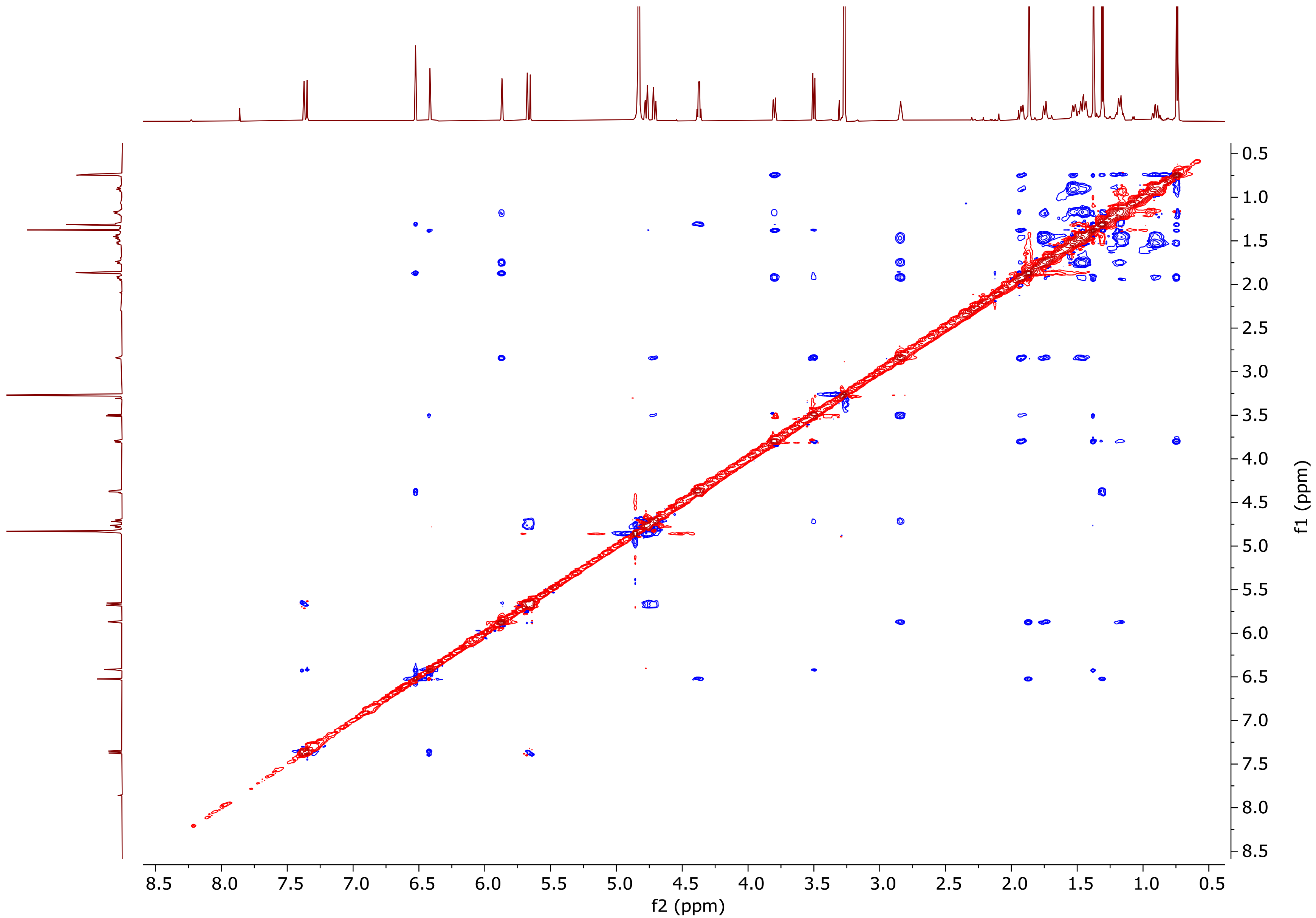

Figure S12. NOESY spectrum of wheldone in CD_3OD.

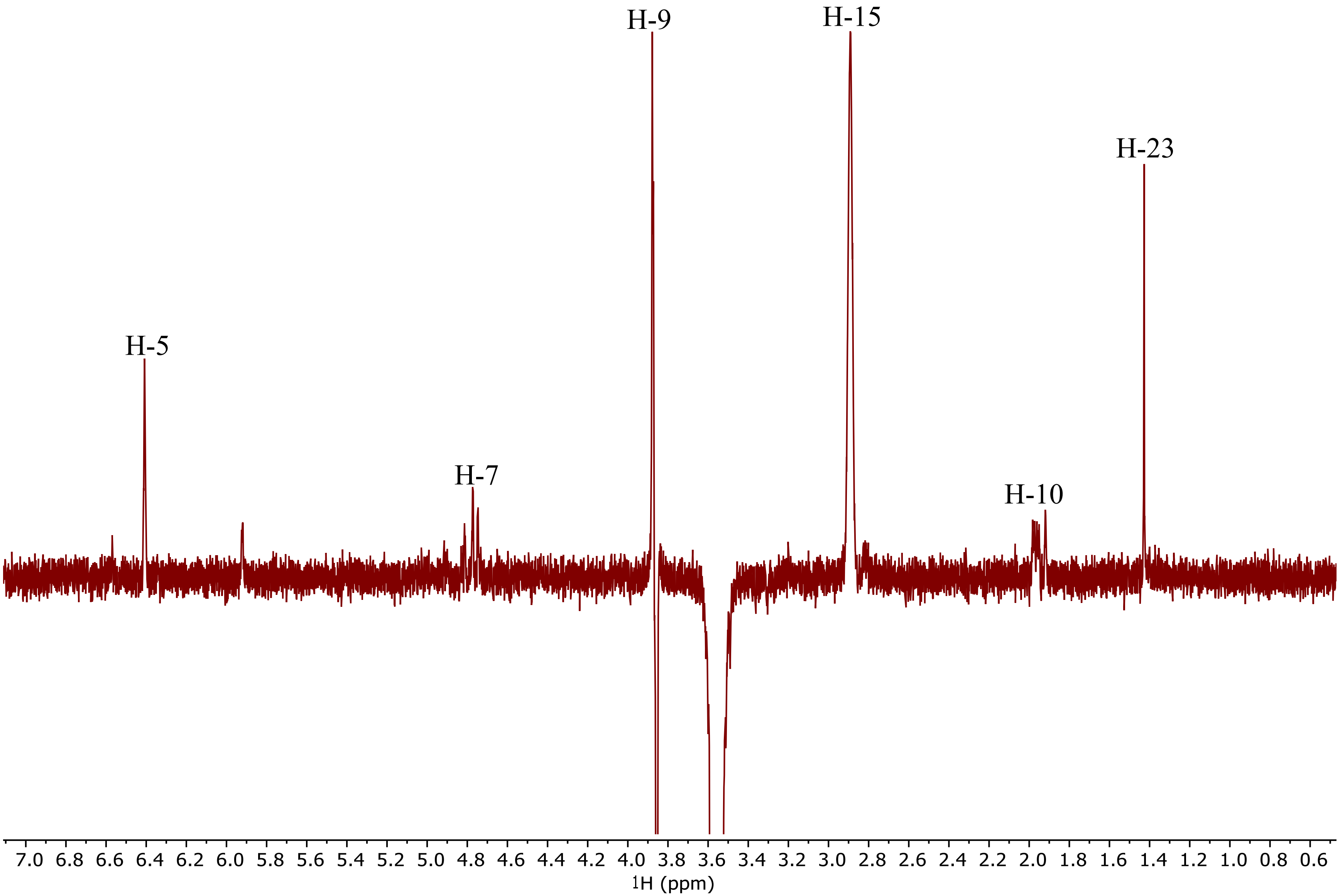

Figure S13. 1D NOE spectrum of wheldone (excitation of H-8) in CD_3OD.

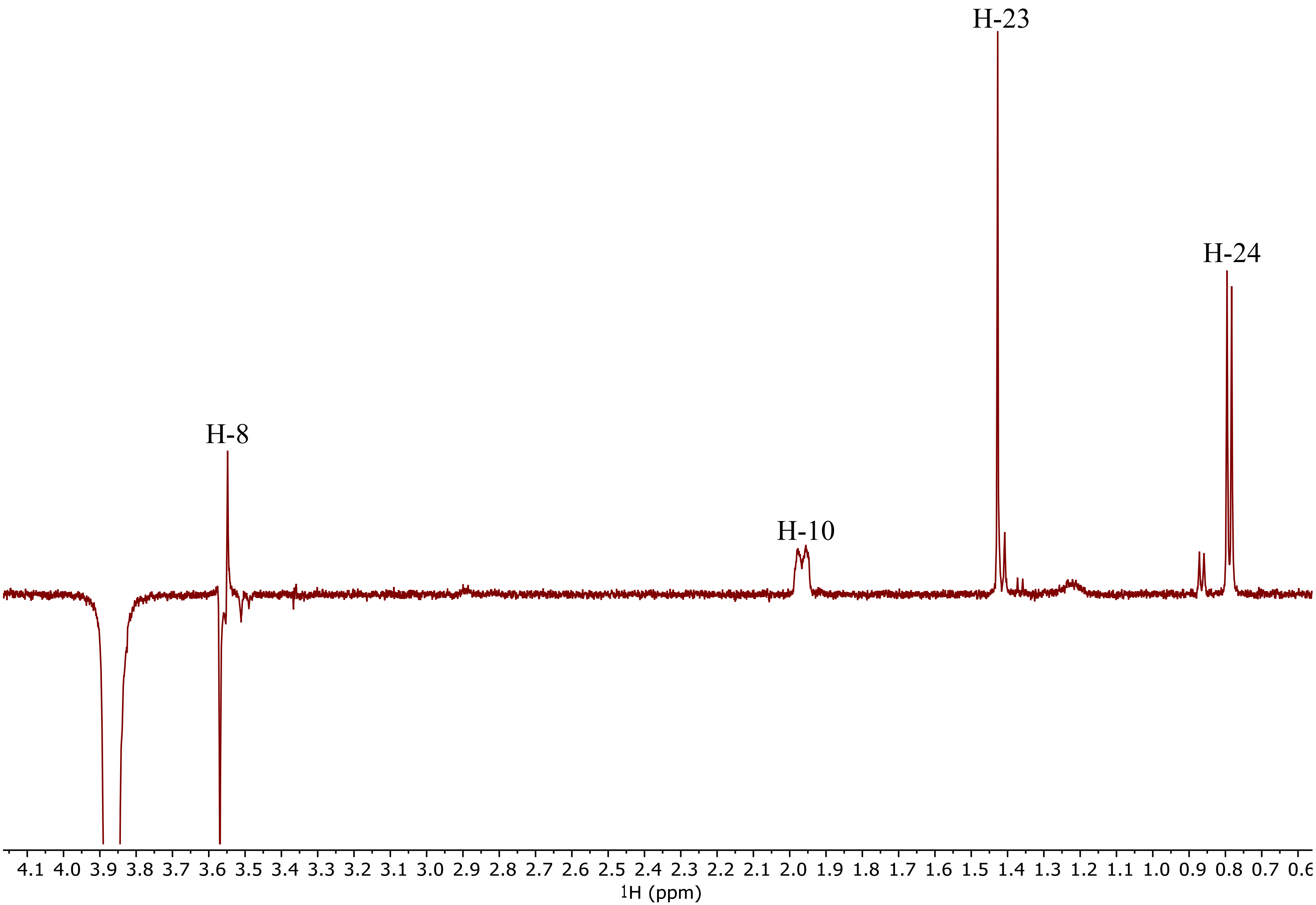

Figure S14. 1D NOE spectrum of wheldone (excitation of H-9) in CD_3OD.

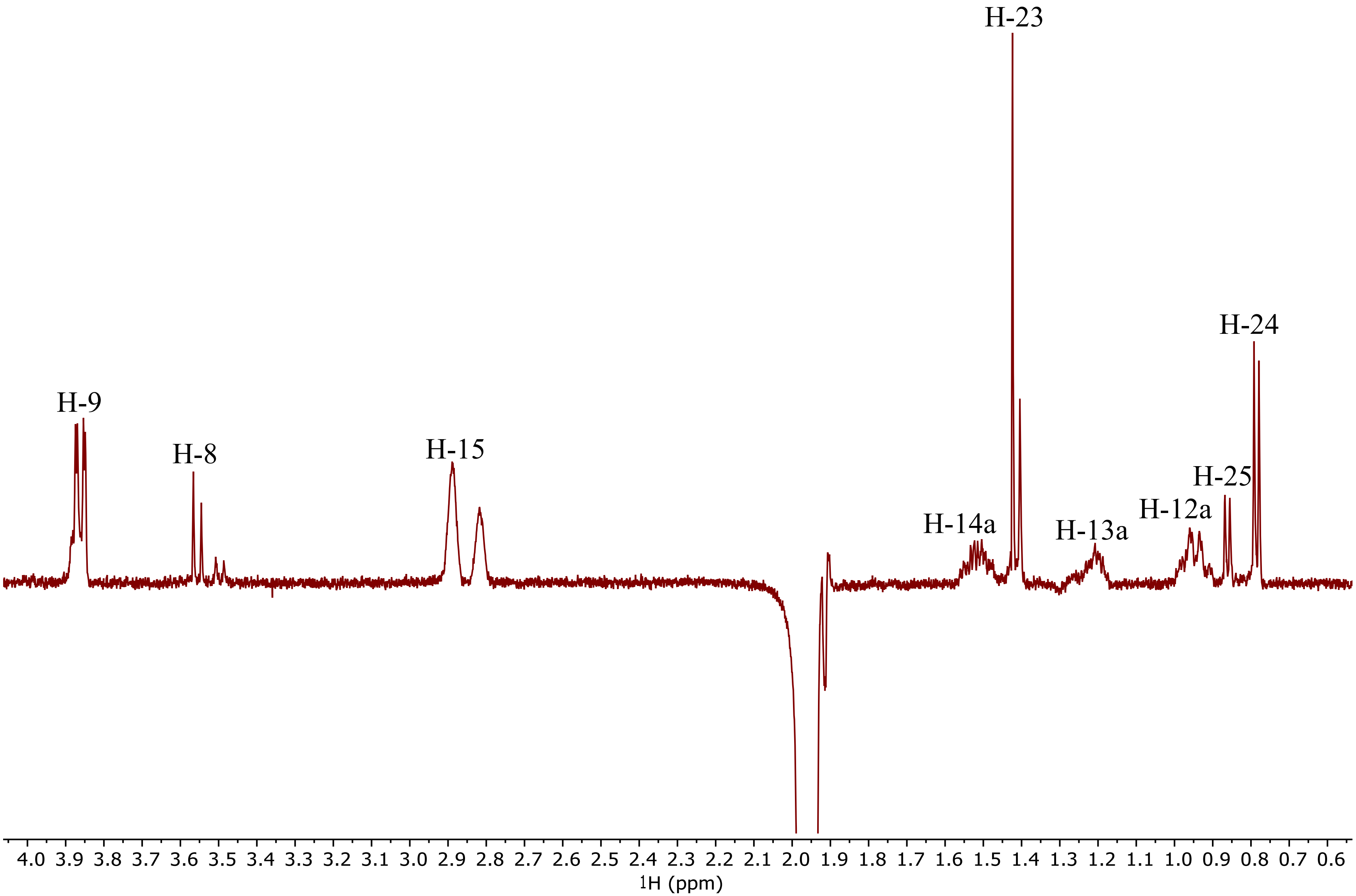

Figure S15. 1D NOE spectrum of wheldone (excitation of H-10) in CD_3OD.

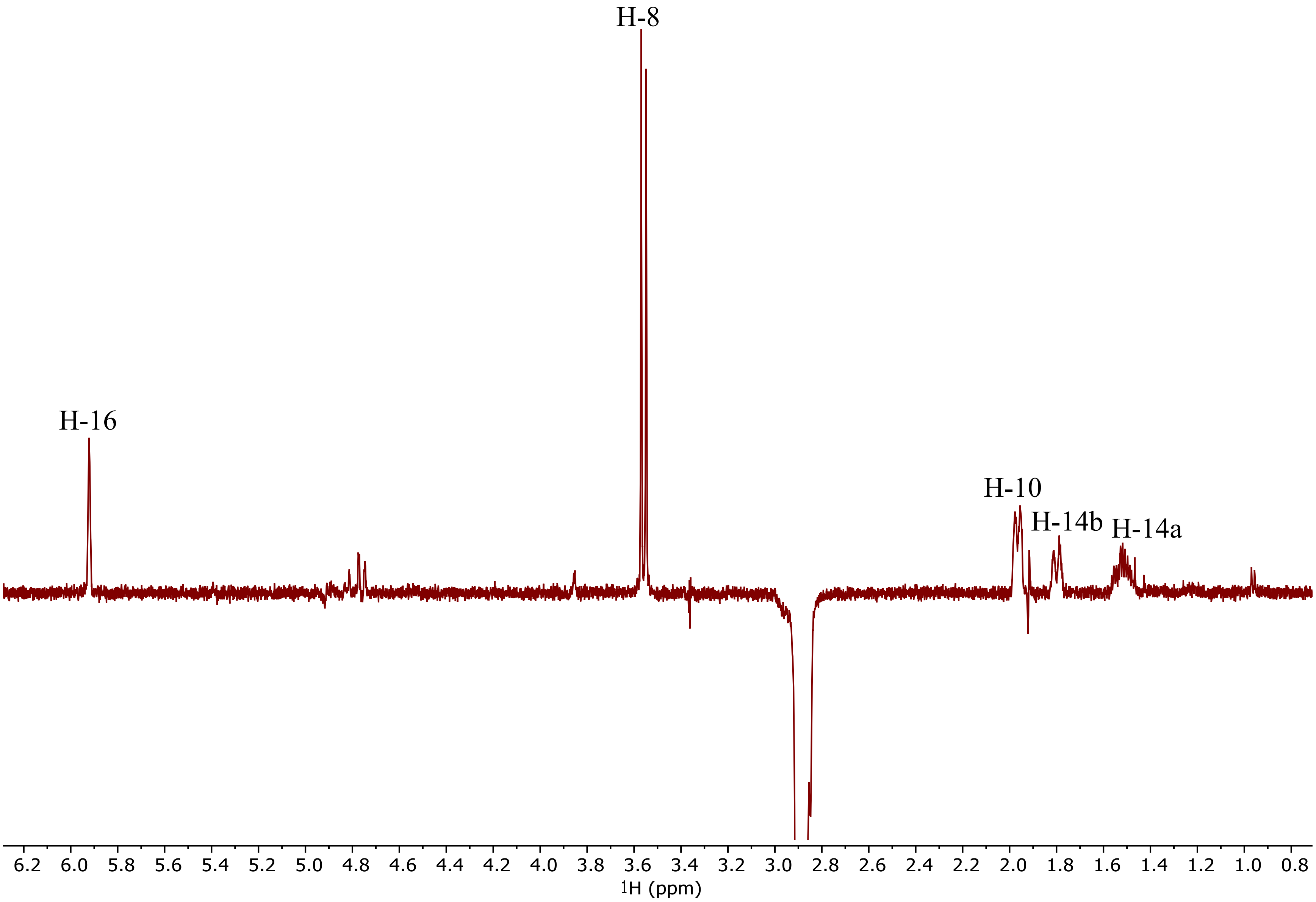

Figure S16. 1D NOE spectrum of wheldone (excitation of H-15) in CD_3OD.

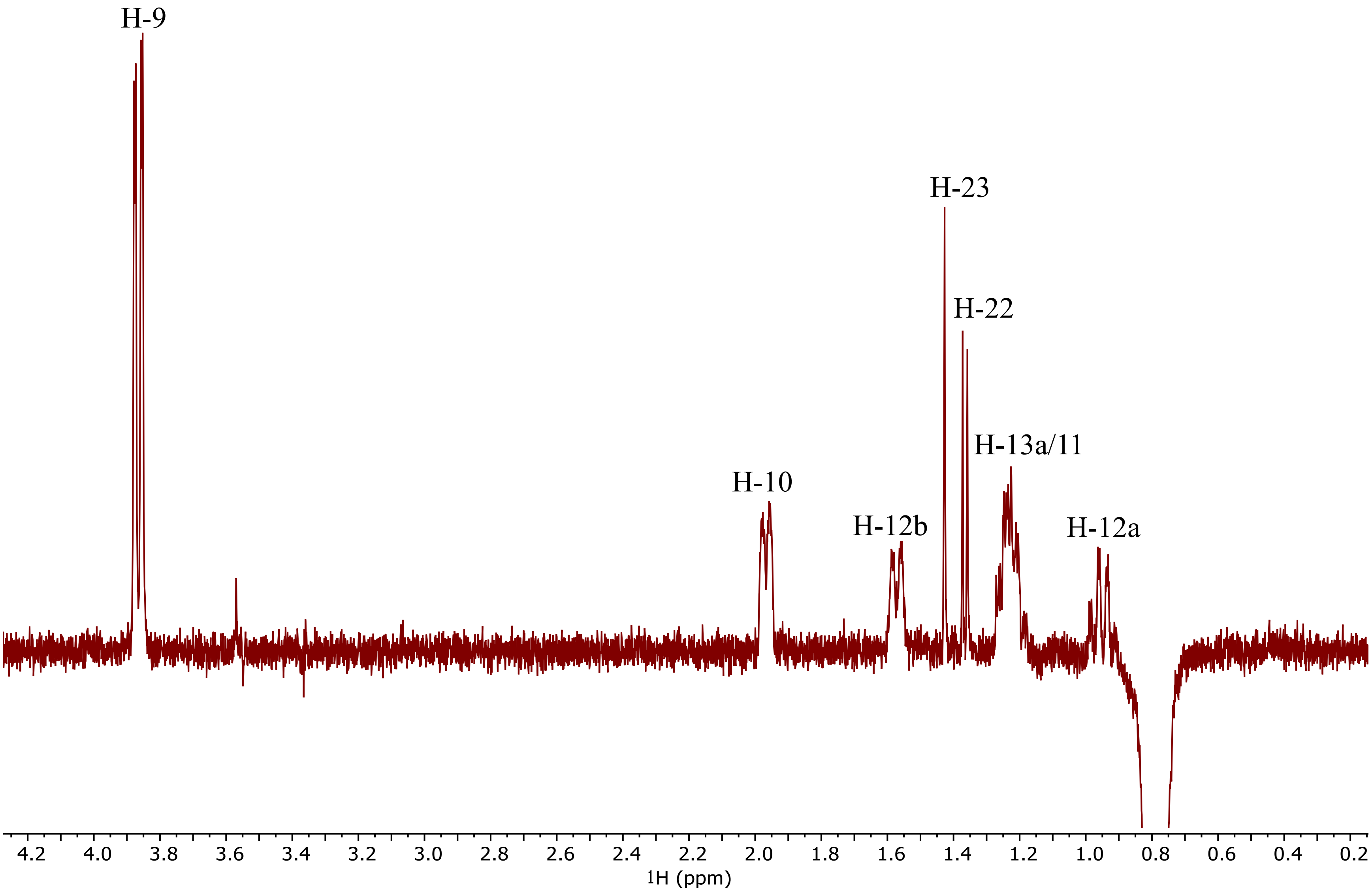

Figure S17. 1D NOE spectrum of wheldone (excitation of H-24) in CD_3OD.

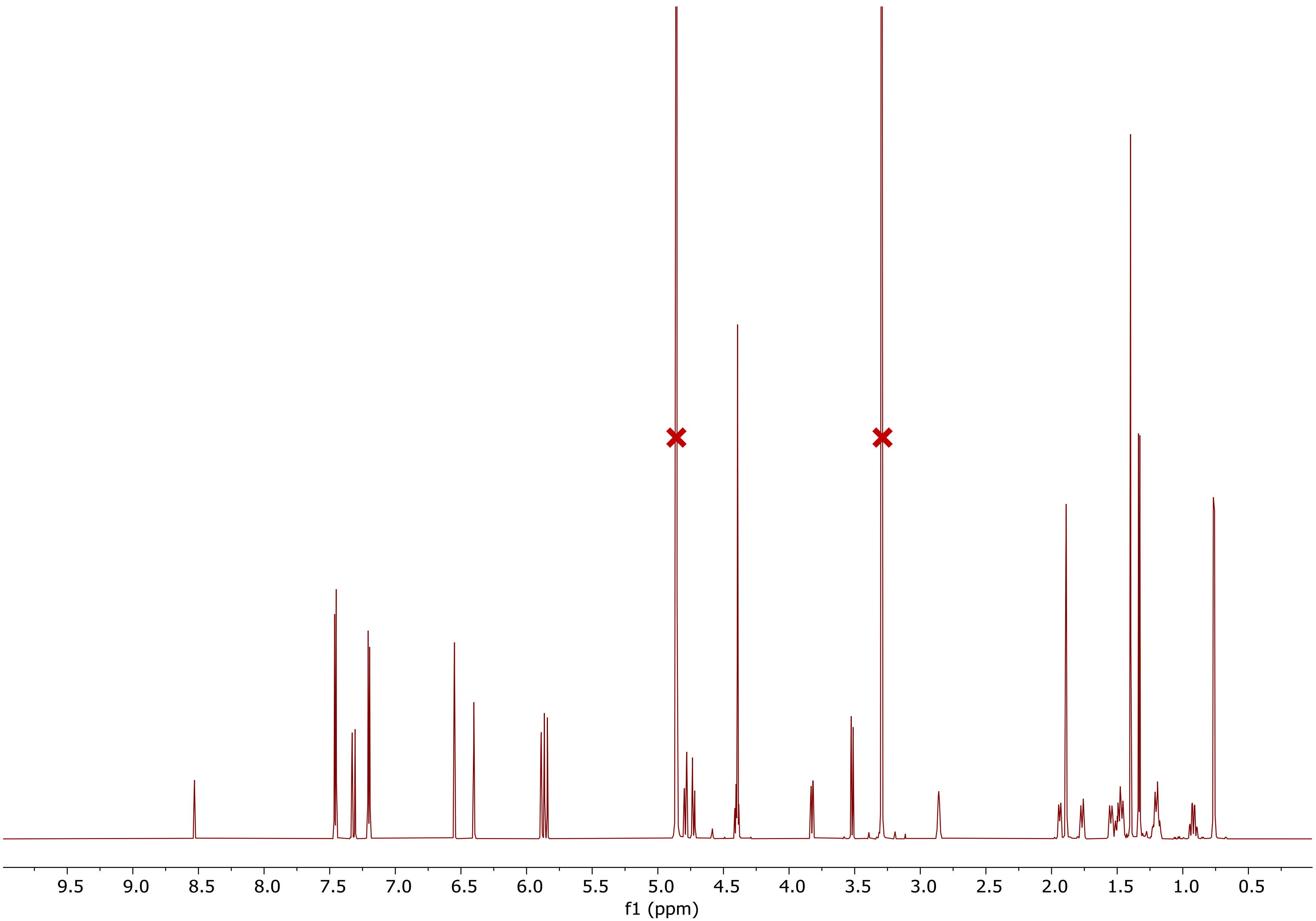

Figure S18. ^{1}H NMR spectrum of wheldone *p*-bromobenzylamide in CD_3OD (700 MHz).

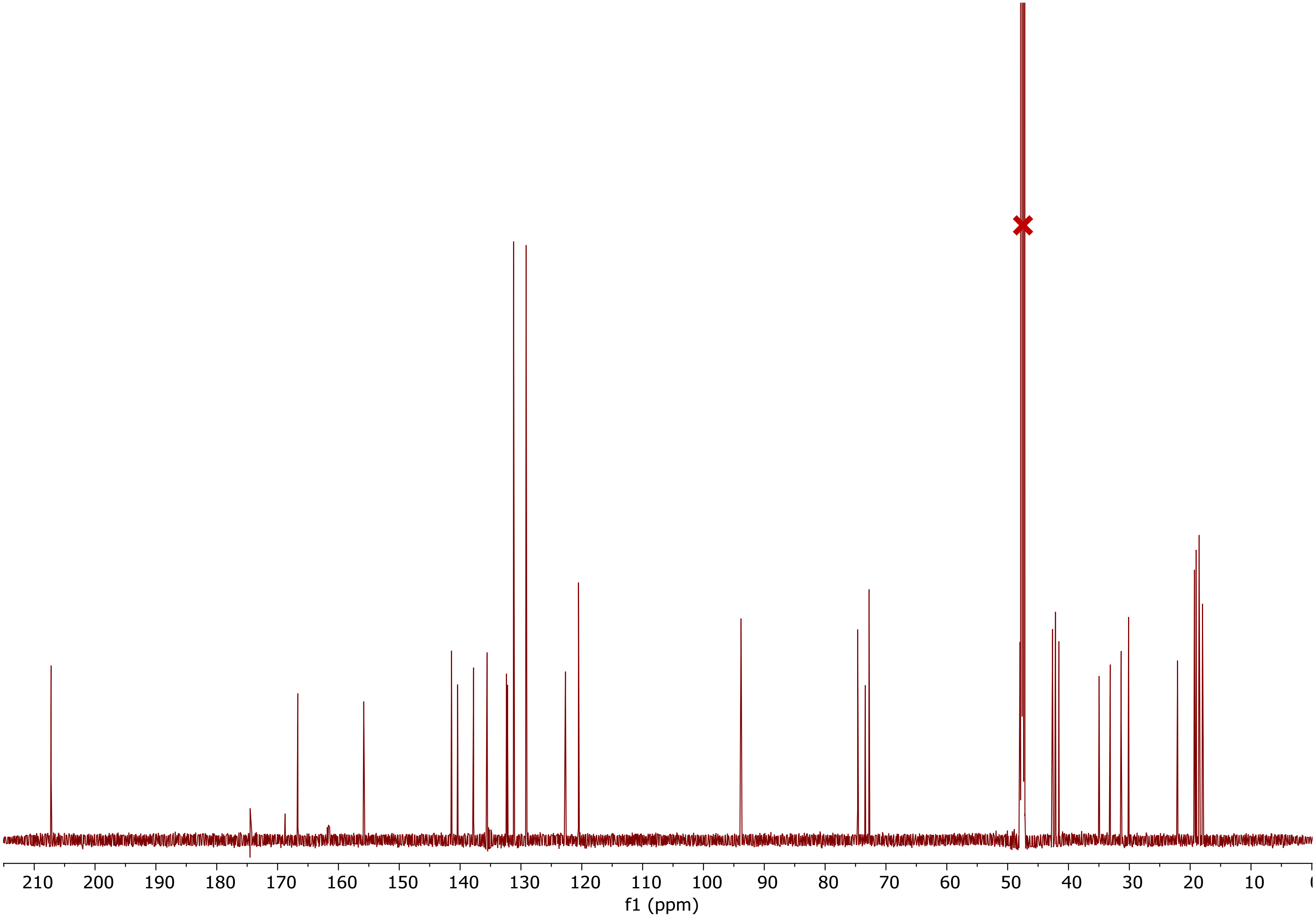

Figure S19. ^{13}C NMR spectrum of wheldone *p*-bromobenzylamide in CD_3OD (175 MHz).

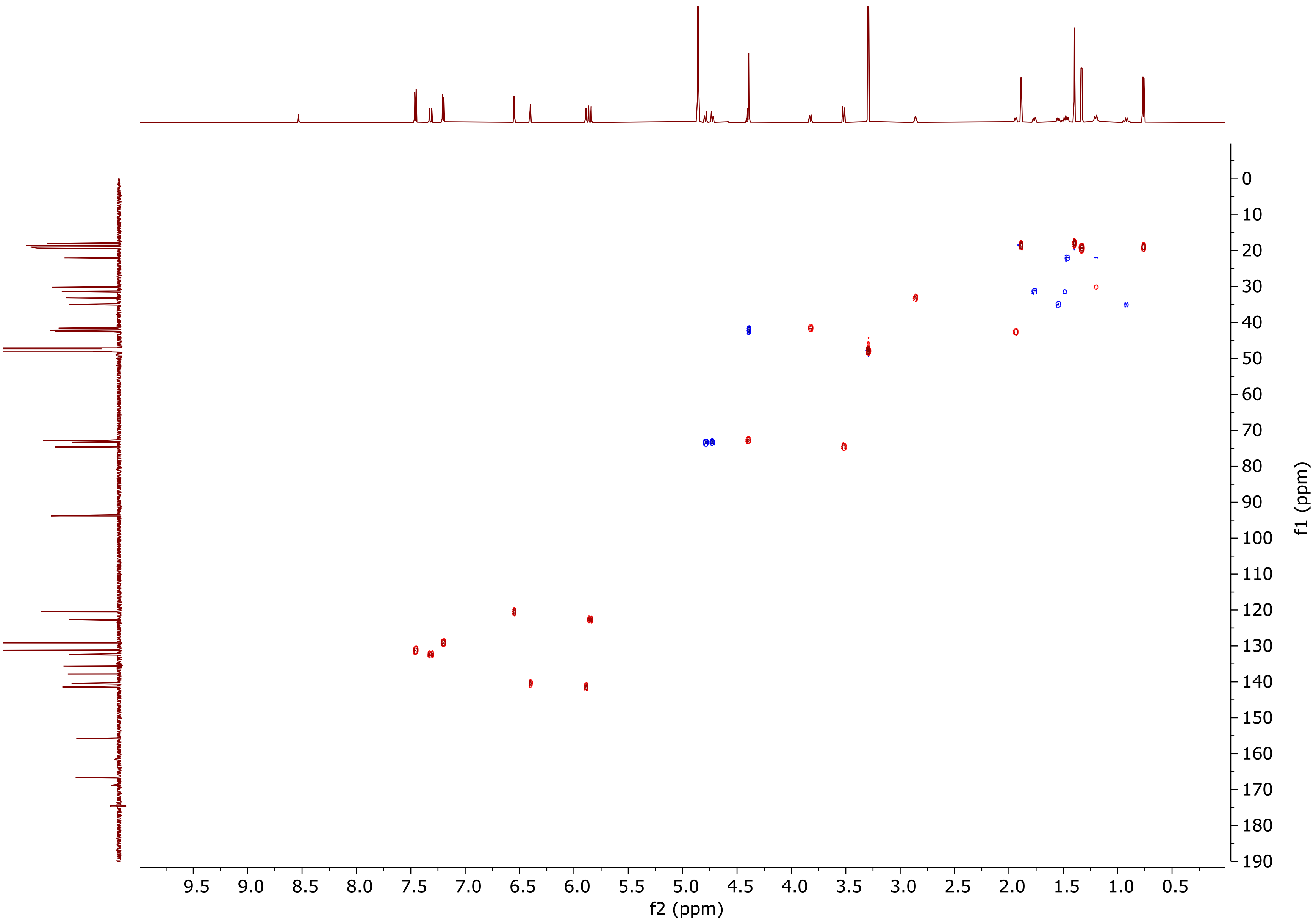

Figure S20. gHSQCAD spectrum of wheldone *p*-bromobenzylamide in CD_3OD.

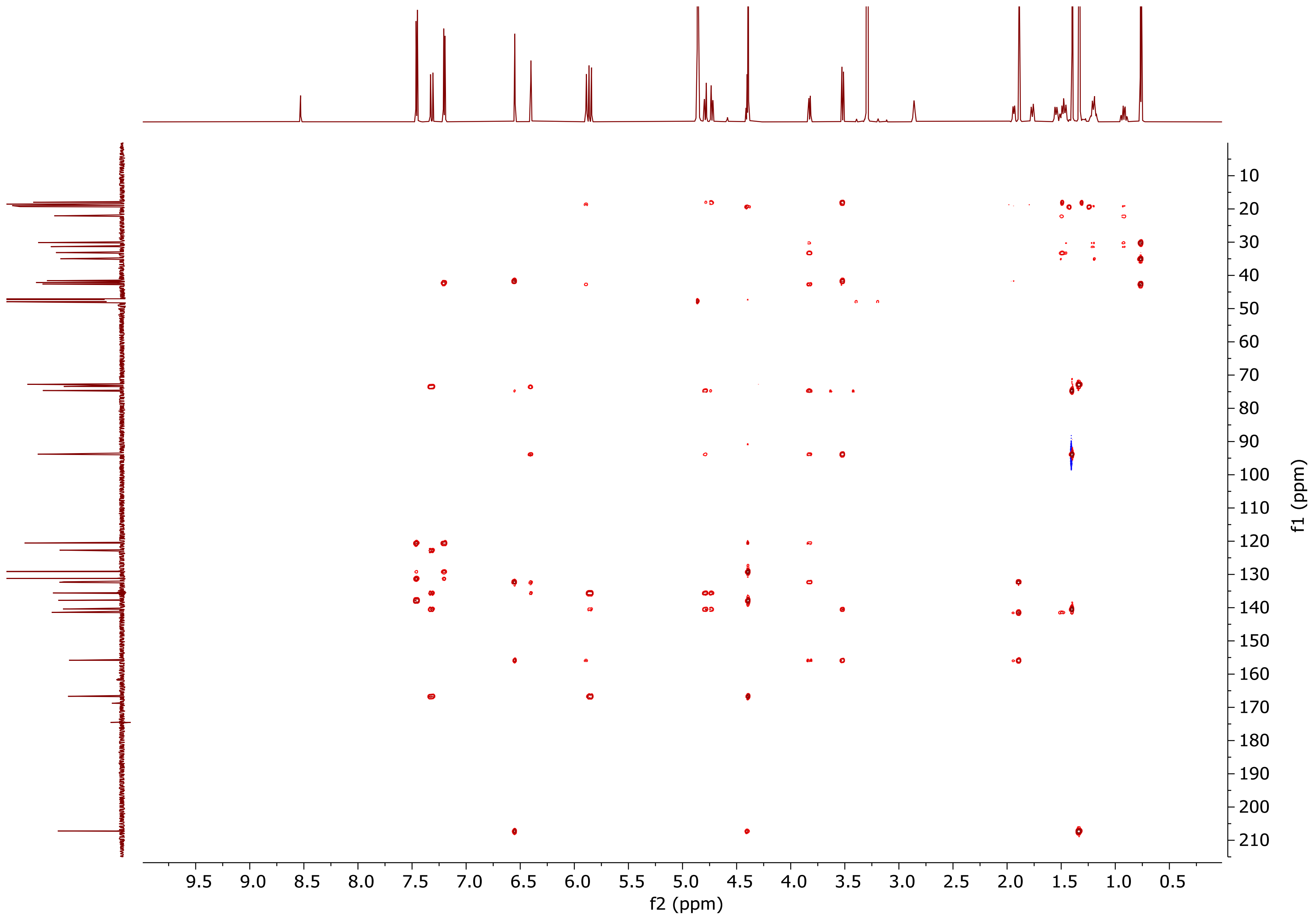

Figure S21. gHMBCAD spectra of wheldone *p*-bromobenzylamide in CD_3OD.

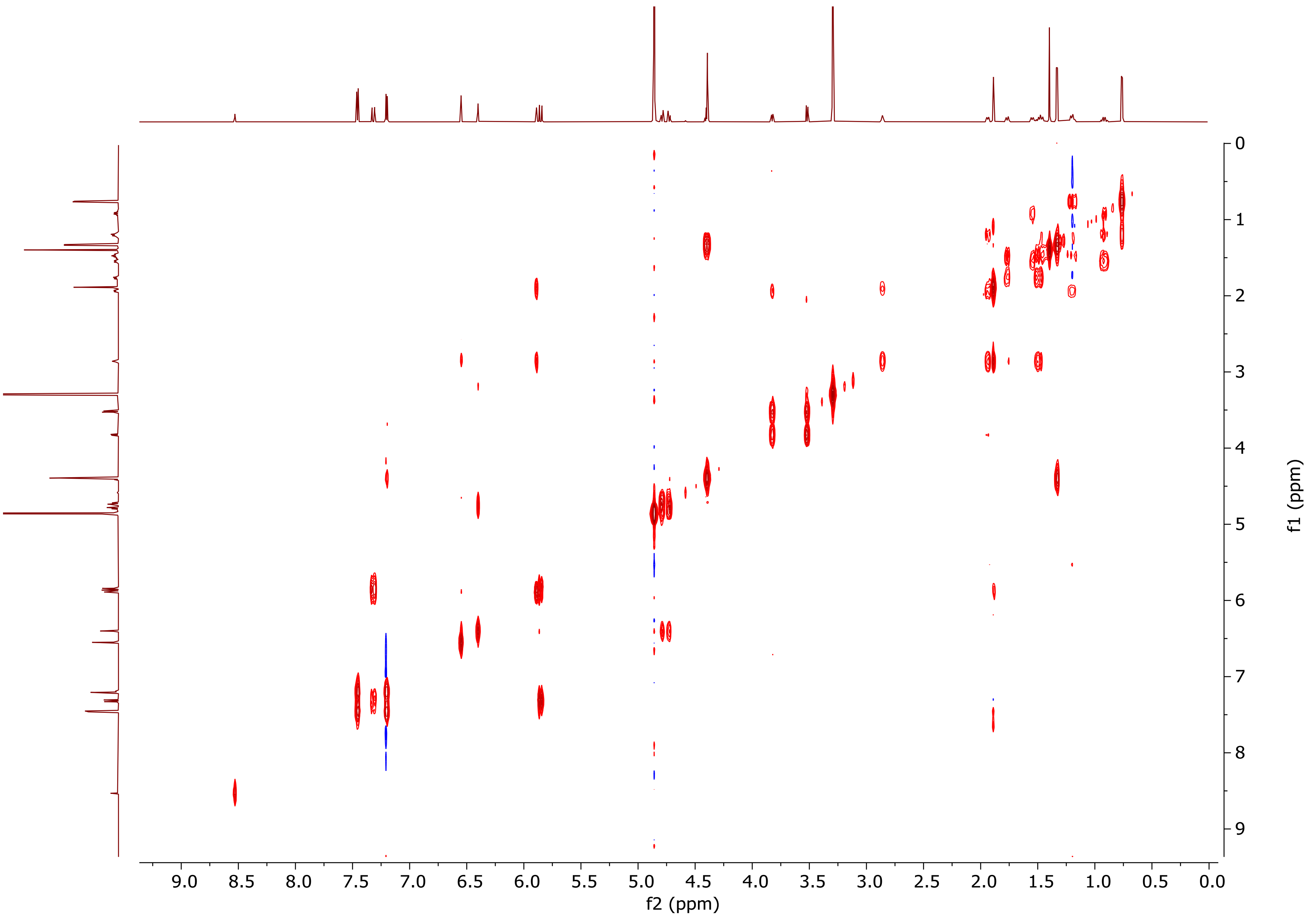

Figure S22. gCOSY spectra of wheldone *p*-bromobenzylamide in CD_3OD.

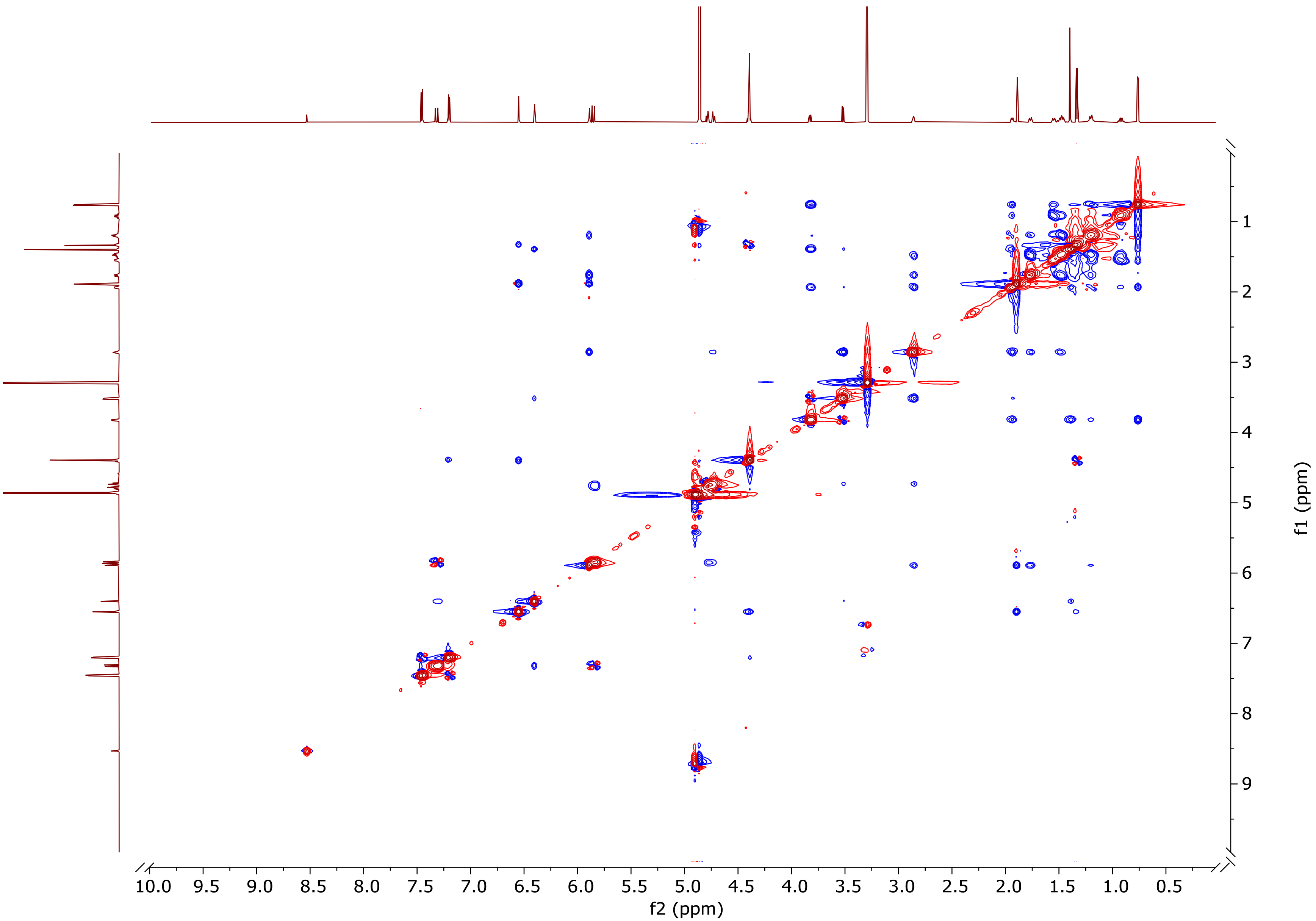

Figure S23. NOESY spectra of wheldone *p*-bromobenzylamide in CD_3OD.

References

(1) Tyler, A. R.; Ragbirsingh, R.; McMonagle, C. J.; Waddell, P. G.; Heaps, S. E.; Steed, J. W.; Thaw, P.; Hall, M. J.; Probert, M. R., Encapsulated nanodroplet crystallization of organic-soluble small molecules. *Chem* **2020,** *6*, 1755-1765.

(2) Palatinus, L., Ab initio determination of incommensurately modulated structures by charge flipping in superspace. *Acta Cryst. A* **2004,** *60*, 604-610.

(3) Palatinus, L.; Chapuis, G., SUPERFLIP - a computer program for the solution of crystal structures by charge flipping in arbitrary dimensions. *J. Appl. Cryst.* **2007,** *40*, 786-790.

(4) Sheldrick, G. M., SHELXT - Integrated space-group and crystal-structure determination. *Acta Cryst. A* **2015,** *71*, 3-8.

(5) Sheldrick, G. M., A short history of SHELX. *Acta Cryst. A* **2008,** *64*, 112-122.

(6) Dolomanov, O. V.; Bourhis, L. J.; Gildea, R. J.; Howard, J. A. K.; Puschmann, H., OLEX2: a complete structure solution, refinement and analysis program. *J. Appl. Cryst.* **2009,** *42*, 339-341.

(7) Knowles, S. L.; Raja, H. A.; Isawi, I. H.; Flores-Bocanegra, L.; Reggio, P. H.; Pearce, C. J.; Burdette, J. E.; Rokas, A.; Oberlies, N. H., Wheldone: Characterization of a unique scaffold from the coculture of *Aspergillus fischeri* and *Xylaria flabelliformis*. *Org. Lett.* **2020,** *22*, 1878-1882.

(8) Pierens, G. K., ^{1}H and ^{13}C NMR scaling factors for the calculation of chemical shifts in commonly used solvents using density functional theory. *J. Comput. Chem.* **2014,** *35*, 1388-1394.
